# Shared local brain dynamics in pediatric and adult non-rapid eye movement parasomnias

**DOI:** 10.1093/sleep/zsag123

**Published:** 2026-05-08

**Authors:** Julian Amacker, Marco Veneruso, Matteo Pereno, Simone Ulzega, Samuel Wehrli, Sven Hirsch, Lino Nobili, Silvia Miano, Mauro Manconi, Anna Castelnovo

**Affiliations:** Institute of Computational Life Sciences, Zurich University of Applied Sciences, Wädenswil, 8820, Switzerland; Department of Neurosciences, Rehabilitation, Ophthalmology, Genetics, Maternal and Child Health (DINOGMI), University of Genova, Genova, 16132, Italy; Sleep Medicine, Neurocenter of Southern Switzerland, Ospedale Civico, Lugano, 6900, Switzerland; Child Neuropsychiatry Unit, IRCCS Istituto Giannina Gaslini, Genova, 16147, Italy; Sleep Medicine, Neurocenter of Southern Switzerland, Ospedale Civico, Lugano, 6900, Switzerland; Institute of Computational Life Sciences, Zurich University of Applied Sciences, Wädenswil, 8820, Switzerland; Institute of Computational Life Sciences, Zurich University of Applied Sciences, Wädenswil, 8820, Switzerland; Institute of Computational Life Sciences, Zurich University of Applied Sciences, Wädenswil, 8820, Switzerland; Department of Neurosciences, Rehabilitation, Ophthalmology, Genetics, Maternal and Child Health (DINOGMI), University of Genova, Genova, 16132, Italy; Child Neuropsychiatry Unit, IRCCS Istituto Giannina Gaslini, Genova, 16147, Italy; Sleep Medicine, Neurocenter of Southern Switzerland, Ospedale Civico, Lugano, 6900, Switzerland; Faculty of Biomedical Sciences, University of Italian Switzerland, Lugano, 6900, Switzerland; Sleep Medicine, Neurocenter of Southern Switzerland, Ospedale Civico, Lugano, 6900, Switzerland; Faculty of Biomedical Sciences, University of Italian Switzerland, Lugano, 6900, Switzerland; Department of Neurology, University Hospital, Inselspital, Bern, 3010, Switzerland; Sleep Medicine, Neurocenter of Southern Switzerland, Ospedale Civico, Lugano, 6900, Switzerland; Faculty of Biomedical Sciences, University of Italian Switzerland, Lugano, 6900, Switzerland; University Hospital of Psychiatry and Psychotherapy, University of Bern, Bern, 3000, Switzerland

**Keywords:** confusional arousal, night terrors, somnambulism, sleepwalking, hdEEG, power, local sleep

## Abstract

**Study Objectives:**

To characterize source-level cortical oscillatory dynamics before and during disorders of arousal (DoA) compared with physiological motor arousals in children and adults.

**Materials and Methods:**

Nineteen children (10.9 ± 3.0 years) and 22 adults (30.3 ± 5.2 years) with DoA underwent 256-channel high-density EEG with video-polysomnography. Source-space spectral analysis in delta (0.5–4 Hz) and beta (18–30 Hz) bands was computed during stable slow-wave-sleep (-3 to -2 minutes before movement onset) and from -5 seconds to +15 seconds around movement onset for 84 DoA episodes and 146 physiological motor arousals. Linear mixed-effects models with permutation-based cluster correction were applied.

**Results:**

Four reproducible cortical hallmarks emerged across age groups: (1) a widespread pre-onset increase in beta and delta relative to slow-wave-sleep; (2) sustained frontal delta enhancement over anterior cingulate, dorsomedial and ventromedial prefrontal, frontopolar, and orbitofrontal cortices; (3) concurrent centro-parietal delta suppression spanning posterior cingulate, precuneus, superior parietal, sensorimotor, and supplementary motor regions; and (4) persistent bilateral beta enhancement. Compared with physiological arousals, DoA arose from a “sleepier” background (higher delta, lower beta) and displayed greater beta coexisting with frontal delta persistence. Children showed broader pre-onset delta effects and more diffuse post-onset persistence, whereas adults exhibited a more anteriorly confined pattern.

**Conclusion:**

DoA display a reproducible, age-invariant cortical signature, consistent with a spatially organized failure of arousal integration: posterior sensorimotor and awareness circuits partially reactivate, while anterior executive and emotion-regulatory regions remain sleep-like. These spatiotemporal fingerprints position hd-EEG source analysis as a translational tool for mechanistically informed diagnostic and therapeutic development.

## Teaser

Using high-density EEG, this study identifies a reproducible cortical signature of disorders of arousal (DoA) that is shared by children and adults and clearly distinct from physiological motor arousals. Episodes emerge from a globally sleep-like brain state and are marked by a stable dissociation between motor networks and regions supporting awareness and executive control. These findings provide direct evidence that DoA involve stable and spatially organized patterns of local brain dissociation, extending previous models with age-invariant and arousal-specific markers. Remaining gaps include how these signatures differ across parasomnia subtypes and whether pre-episode EEG patterns can be leveraged for prediction, prevention, or improved differential diagnosis with nocturnal epileptic events.

## Graphical Abstract

**Graphical Abstract:**
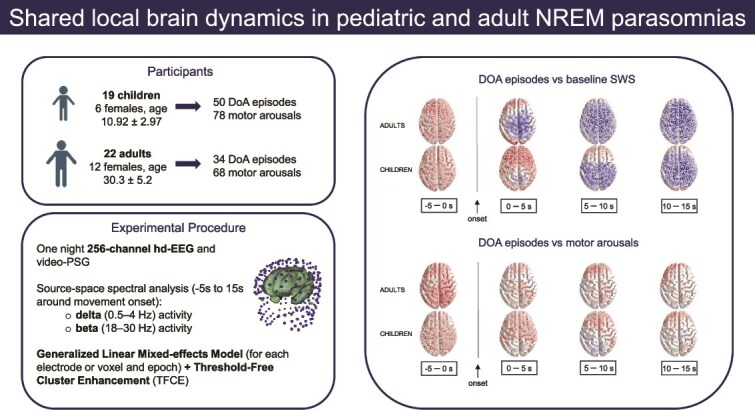

## Introduction

Sleepwalking, sleep terrors, and confusional arousals are non-rapid eye movement (NREM) sleep parasomnias [1], collectively known as Disorders of Arousal (DoA). These episodes arise from incomplete awakenings during slow-wave sleep (SWS) [2] and are characterized by complex behaviors, such as sitting up, walking, or speaking, reduced awareness and responsiveness to the environment, attenuated pain sensitivity, variable autonomic activation, and dream-like mentation [3]. Far from being rare curiosities, these phenomena blur the boundary between sleeping and waking, offering a striking glimpse into how consciousness, behavior, and emotion can come apart. Beyond their scientific interest, DoA can disrupt sleep, cause psychosocial distress [4], accidental injury, and, in rare cases, legal consequences [5]. Despite their prevalence, up to 13% in childhood and 2% in adults [6, 7], and their broader social impact, their underlying neurophysiology remains incompletely defined, limiting diagnostic precision and therapeutic options [8].

Sporadic single-photon emission computed tomography (SPECT) [9] and intracerebral EEG (SEEG) recordings during epilepsy surgery evaluations [10–13] have shown that DoA episodes often feature a mixture of slow-wave “sleep-like” activity and faster “wake-like” rhythms, consistent with local sleep–wake dissociation [2]. However, these techniques are invasive or impractical for repeated, systematic evaluation across multiple episodes. Scalp EEG [14, 15] offers greater accessibility but lacks the spatial resolution to fully map local cortical dynamics. Nearly all scalp EEG studies to date have focused on adults [14–25], leaving it unclear whether these features reflect universal mechanisms or age-specific processes. Furthermore, the extent to which DoA differ from physiological arousal processes, particularly in the seconds following movement onset, remains poorly understood, partly due to the difficulty of retaining clean EEG during movement-rich events. Only one study has examined the post-onset phase in adults [16], and none in children.

Here, we address three key gaps in DoA neurophysiology. First, we provide the first fine-grained, source-space mapping of cortical oscillatory dynamics before and after DoA onset in adults, leveraging high-density EEG, a technique enabling 3D source modeling of cortical activity with spatial resolution comparable to PET [26]. Second, we test whether these spatiotemporal patterns are conserved across age, indicating core mechanisms rather than age-specific phenomena, or whether they are specific to more severe cases that persist into adulthood. Third, we determine how DoA differ from physiological motor arousals in both children and adults, before and after movement onset, to clarify whether DoA follow an exaggerated, delayed, or qualitatively distinct arousal trajectory, irrespective of age.

## Materials and methods

This observational, intra-group, monocentric study was conducted at the Sleep Medicine Unit of the Neurocenter of Southern Switzerland at the Civic Hospital of Lugano between July 2018 and April 2023. All study procedures were reviewed and approved by the Local Ethics Committee (2017-01788 CE 3282) and conducted in accordance with the Declaration of Helsinki.

### Participants

Patients were recruited either from outpatients attending our sleep medicine center (during routine visits or retrospectively through the electronic hospital system) or from the general population via word of mouth. Inclusion criteria comprised a clinical diagnosis of DoA based on ICSD-3 criteria [1] and an age range of 5–17 years (children’s group) or 18–45 years (adult group). Exclusion criteria included major psychiatric or neurological conditions (e.g. epilepsy), use of psychotropic medications, and an apnea-hypopnea index (AHI) >5 events/hour for children or > 15 events/hour for adults. Following an in-person or telephone screening to assess eligibility, all patients, and, when available, their parents or bedpartners, underwent a comprehensive clinical evaluation by a physician board-certified in sleep medicine. Relevant clinical data, including age, sex, family history of parasomnia, age of onset, and episode frequency at the time of observation, were collected. Informed consent was obtained from all participants prior to study initiation.

### Experimental procedure

All enrolled patients underwent at least one nocturnal polysomnography (PSG) recording in our sleep laboratory (~09:00 p.m. to ~07:00 a.m.). Non-EEG channels were recorded using an Embla N7000 PSG System and included video, electro-oculogram (EOG), electromyogram (EMG) of the submentalis muscle, the right and left tibialis anterior muscles and extensor digitorum communis, electrocardiogram, oral and nasal airflow thermistors, nasal pressure cannula, and wearable piezo-electric bands for thoracic and abdominal movements. EEG was recorded using a high-density EEG system (256 channels; Electrical Geodesics Inc., Eugene, OR, vertex referencing, sampling rate of 500 Hz).

Technicians intervened in DoA episodes only if the episode lasted more than 20-30 seconds. In such cases, the experimenter called the patient’s name and asked simple questions, such as “Where are we?” and “Can you name this object?”

### Sleep scoring

Sleep staging and the scoring of associated sleep events was performed by a board-certified sleep physician (AC) according to standard AASM criteria [27] using EMBLA-RemLogic software. Sleep staging was based on 30-second epochs for 6 standard EEG derivations extracted from the high-density EEG system, with bipolar re-referencing (F3/M2, F4/M1, C3/M2, C4/M1, O1/M2, O2/M1), and synchronized with the other PSG channels.

### Scoring of parasomnia episodes and of normal arousals

DoA episodes were defined as sudden behaviors emerging against a sleep EEG background and characterized by eye opening and/or vocalizations, movements of the head or limbs, trunk flexion, exploratory actions, or abrupt expressions of fear. Typical motor arousals were identified as EEG arousals associated with physiological movements, such as changing body position, adjusting posture, scratching, stretching, and rearranging clothing or blankets.

To enhance the accuracy and consistency of evaluating behavioral episodes, three raters certified in sleep medicine (AC, SM, LN for children; AC, MV and LN for adults) independently viewed all the video-EEG recordings and classified the behavior as either a parasomnia episode or a typical motor arousal. Discordant cases were resolved by discussion. Events for which the three experts could not reach a consensus were excluded from the analysis. These cases typically involved brief episode duration, uncertain eye opening, and poor video quality, or situations in which the patient was not facing the camera or was partially covered by bedsheets. The beginning of a motor event (either a parasomnia episode or a typical motor arousal) was defined as the onset of movement detected on one of the five EMG channels available (submental chin, bilateral arms, bilateral legs), whichever occurred first. The end of a motor event was determined by the definite cessation of movement, the appearance of a clear wake EEG pattern, or the patient’s ability to correctly answer two consecutive questions in cases where the technician intervened. Events with brief interruptions of motor activity, lasting fewer than 5 seconds and followed by the resumption of movement, were considered a single episode.

### E‌EG data extraction

EEG data analysis was performed in MATLAB (The MathWorks Inc., Natick, MA, USA) using the EEGLAB v13 [28].

Initially, 15-minute EEG segments were extracted, comprising 10 minutes of sleep EEG preceding motor onset and 5 minutes following motor onset. These segments were then band-pass filtered between 0.5 and 45 Hz. To avoid edge distortion introduced by filtering, shorter 5-minute segments were subsequently retained, spanning from 3 minutes before to 2 minutes after motor onset.

### E‌EG artifact removal procedure

Channels affected by artifacts (either for the entire segment or for 5-second sub-epochs) were visually inspected and labeled as bad in Brainstorm [29]. Artifact Subspace Reconstruction (ASR) was then applied, after excluding identified bad channels. ASR adaptively filters out artifacts by modeling clean EEG segments, automatically selected based on deviations from the power distribution in each channel, and rejecting outliers in the PCA subspace. A moving window of 768 ms (1.5 × the number of channels) and a 35 standard deviation cutoff for rejection were used to avoid excluding sleep features, such as slow waves persisting during the arousal/episode. This threshold is consistent with the default value of the Dusk2Dawn automatic cleaning procedure [30].

To remove ECG and muscle artifacts, wavelet-enhanced ICA (wICA) was applied using a customized version of the EEGLAB “RELAX” plugin [31]. ICLabel classification was adapted for hd-EEG by labeling components as artifactual if classified as “bad” (excluding “brain” and “other”) with >70% probability. This selectively removed noise while preserving neural signals. The same wICA, ASR, and ICA procedures were used for both parasomnia episodes and typical motor arousals.

### Power spectral density decomposition

Power spectral density (PSD) was computed at the source level using Welch’s modified periodogram method, with 5-second Hamming windows and 50% overlap. The analysis focused on the average PSD magnitude within the delta (0.5–4 Hz) and beta (18–30 Hz) frequency bands. At the scalp level, bad channels were interpolated using spherical spline.

### Source modeling

Source modeling was performed with Brainstorm using an age-appropriate template [32], segmented using SPM12/CAT12 Matlab toolbox [33]. A symmetric Boundary Element Method (BEM) volume conduction model of the head having three realistic layers (scalp, inner skull, outer skull) [29] and a standard co-registered set of electrode positions were used to construct the forward model. The inverse matrix was computed using the sLORETA Minimum Norm [34] with sources constrained to be perpendicular to the cortical surface and retaining only diagonal elements of the noise covariance matrix. Source estimates were computed using sLORETA, which provides noise-normalized current density estimates by scaling the source activity at each location by its estimated variance, derived from the noise covariance and forward model.

Cortical regions were defined based on the Destrieux atlas [35] and subsequently aggregated into 22 functional macro-regions according to their anatomical and functional correspondence. Cingulate cortex was subdivided into anterior cingulate cortex (ACC), mid-cingulate cortex (MCC), and posterior cingulate cortex (PCC). Frontal regions were grouped into dorsolateral prefrontal cortex (DLPFC), ventrolateral prefrontal cortex (VLPFC), frontopolar cortex (FPC), orbitofrontal cortex (OFC), dorsomedial prefrontal cortex (dmPFC), and ventromedial prefrontal cortex (vmPFC). Parietal regions comprised superior parietal lobule (SPL) and temporoparietal junction (TPJ). Temporal regions were parsed into primary auditory cortex (A1), superior temporal gyrus (STG), and middle/inferior temporal gyrus (MTG/ITG). Occipital regions were subdivided into primary visual cortex (V1), extrastriate and lateral occipital cortex (V2/LOC), and ventral temporal/occipitotemporal cortex (VTC). Medial structures including the precuneus were grouped as core nodes of the default mode network. Motor and somatosensory regions were defined based on precentral (primary motor cortex, M1), postcentral (primary somatosensory cortex, S1), and medial frontal areas (supplementary motor area, SMA).

### Statistical analyses

Statistical comparisons between DoA episodes and baseline sleep were performed using a linear mixed-effects model (LME) for each electrode or voxel and epoch, using the lmeEEG Matlab routine [36]. This method reduces computational load by isolating marginal EEG data for standard regression.

Two primary comparisons were conducted: DoA/baseline and DoA/“physiological” motor movement. Dependent variables included EEG-derived measures such as scalp or source PSD. Episode onset (from sleep onset) and age were included as fixed effects, whereas adding sex yielded no substantive improvement in ΔAIC and ΔBIC. We therefore adopted the more parsimonious model excluding sex. Subjects were treated as a random factor to account for the fact that participants contributed multiple episodes. To create the null distribution, condition labels were shuffled within subjects: 2000 iterations for the DoA/baseline contrast and 3000 iterations for the DoA/motor contrast—the latter increased because *p*-values lay closer to the significance threshold and thus required a more robust estimate. For the DoA/motor model, lmeEEG was further adapted to implement the Freedman–Lane permutation scheme, ensuring valid permutations even for participants who contributed only one of the two conditions (DoA or motor) [37].

To directly compare spectral dynamics between age groups, we extended the primary LME model by adding a group × condition interaction term, where group referred to children vs. adults and condition to DoA vs. SWS or DoA vs. physiological motor arousals. Source-level PSD values were z-score normalized across all subjects prior to this analysis to ensure comparability between children and adults.

## Results

### Participants

High-quality hd-EEG recordings were obtained from 22 adults (mean age 30.3 ± 5.2 years, 12 females) and 19 children (mean age 10.9 ± 3.0 years, 6 females). After exclusion for sleep apnea or epileptiform activity, the final analyses included 20 adults and 17 children. Clinical and vPSG features are summarized in Tables S1–S4. None of the participants were taking psychotropic medications or drugs on the night of the study.

### Behavioral characterization of NREM parasomnia episodes and typical motor arousals

Of 157 SWS motor events in adults, 55 were excluded for lack of consensus among scorers, 34 were identified as parasomnia episodes and 68 as physiological motor arousals. From 154 SWS motor events in children, 26 were excluded for the same reason, leaving 50 DoA episodes and 78 physiological motor arousals.

Parasomnia behaviors most often consisted of brief confusional arousals with eye opening (observed in 91% of adults, in 82% of children), accompanied by sitting up, staring or perplexed gaze, and exploratory head movements. Sleep talking occurred occasionally (29% in children, 9% in adults), as did hallucinatory behaviors, such as imaginary conversations or pointing at nonexistent objects (12% in children, none in adults). Loud screaming was noted in (8%) of children’s events while a single pediatric episode involved an attempt to sleepwalk.

Physiological motor arousals typically involved turning over in bed, adjusting bedclothes, or scratching with eyes closed. See Table S5 for other details.

### Spectral power dynamics from SWS to NREM parasomnia episodes in adults

**Figure 1:**
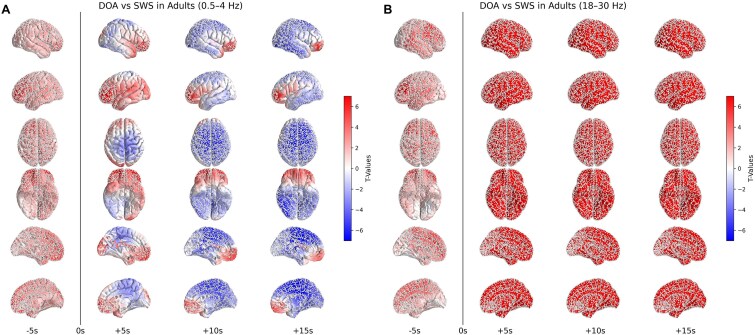
Spatiotemporal spectral dynamics around motor onset during DoA episodes in adults. (A) Source-level t-maps of delta power (0.5–4 Hz) show differences between DoA events and stable slow-wave sleep (SWS) baseline (−3 to −2 min), across four windows from −5 to +15 seconds relative to movement onset (0 s). Warmer colors (red) indicate increased power; cooler (blue) indicate decreased power during parasomnia. Notably, a widespread fronto-temporal/midline increase is seen at −5 s, followed by a central fronto-parietal suppression at +10 to +15 s. (B) Source-level t-maps of beta power (18–30 Hz) across the same windows show a symmetrical, widespread increase in activity prior and during DoA episodes. White dots indicate scouts with significant differences (pTFCE-corrected). All maps are thresholded by corrected t-values.

In the 5 s preceding movement onset, source-level analysis revealed a robust delta power increase relative to stable SWS baseline (−3 to −2 min). This increase involved a broad frontal–midline network including prefrontal, cingulate and sensorimotor cortices, specifically the DLPFC (extending into the inferior frontal gyrus), vmPFC, VLPFC, FPC, SMA, S1–M1, ACC, MCC, and subcallosal cortex. Temporal and parietal contributions (mainly the TPJ and SPL) were more limited and predominantly left-lateralized (Figure 1A, Table 1, Table S6).Across the first 15 s after onset, frontal delta enhancement persisted, most prominently in the FPC, OFC, and vmPFC regions, with the highest density of significant vertices located in the transverse frontopolar gyri and sulci and frontomarginal regions. In parallel, a centro-parietal delta suppression emerged within 5 s, primarily involving the PCC, SMA and precuneus, and extending to S1-M1 and the MCC. This suppression intensified over the 5–15 s interval, progressively spreading to lateral and ventral parieto-temporo-occipital cortices.

**Table 1:** Delta-band (0.5–4 Hz) source-space changes during DoA episodes relative to baseline SWS in children and adults

Brain region	Functional role	Phase	Children	Adults
ACC (anterior cingulate cortex, BA24/32)	*Salience / conflict monitoring*	*Pre-onset*	**↑↑↑**	**↑↑↑**
		*Post-onset*	**↑↑↑** | **↑↑↑** | **↑↑**	**↑↑** | **↑↑↑** | **↑↑↑**
PCC (posterior cingulate cortex, BA23/31)	*Default mode / self-referential*	*Pre-onset*	**↑↑**	**↑↑**
		*Post-onset*	**↑↑↑** | **↓↓↓** | **↓↓**	- | **↓↓↓** | **↓↓↓**
mPFC (medial prefrontal cortex, BA10/32)	*Default mode / self-regulation*	*Pre-onset*	**↑↑↑**	**↑↑↑**
		*Post-onset*	**↑↑↑** | **↑↑↑** | **↑↑↑**	**↑↑↑** | **↑↑↑** | **↑↑↑**
DLPFC (dorsolateral prefrontal cortex, BA9/46)	*Executive control*	*Pre-onset*	**↑↑**	**↑↑↑**
		*Post-onset*	**↑↑↑** | **↑↑↑** | **↑↑**	**↑↑** | **↑** | **↑↑**
FPC (frontopolar cortex, BA10)	*Prospective memory / integration*	*Pre-onset*	**↑↑↑**	**↑↑↑**
		*Post-onset*	**↑↑↑** | **↑↑↑** | **↑↑↑**	**↑↑↑** | **↑↑↑** | **↑↑**
OFC (orbitofrontal cortex, BA11/12)	*Reward / affective valuation*	*Pre-onset*	**↑↑↑**	**↑↑↑**
		*Post-onset*	**↑↑↑** | **↑↑↑** | **↑↑↑**	**↑↑↑** | **↑↑↑** | **↑↑↑**
Precuneus (precuneus, BA7/31)	*Default mode / episodic memory*	*Pre-onset*	**↑↑**	**↑↑↑**
		*Post-onset*	**↑↑** | **↓** | **↓↓**	- | **↓↓↓** | **↓↓↓**
SPL (superior parietal lobule, BA7)	*Visuospatial integration*	*Pre-onset*	**↑↑**	**↑↑↑**
		*Post-onset*	**↑↑** | **↓↓↓** | **↓↓↓**	- | **↓↓↓** | **↓↓↓**
TPJ-L (left temporoparietal junction, BA39/40/22)	*Social cognition / attention*	*Pre-onset*	**↑↑↑**	**↑↑↑**
		*Post-onset*	**↑↑↑** | **↓↓↓** | **↓↓**	**↑↑** | **↓** | **↓↓**
TPJ-R (right temporoparietal junction, BA39/40/22)	*Social cognition / attention*	*Pre-onset*	**↑↑**	**↑↑↑**
		*Post-onset*	**↑↑↑** | **↓↓↓** | **↓↓↓**	**↑** | **↓↓↓** | **↓↓↓**
M1 (primary motor cortex, BA4)	*Voluntary movement execution*	*Pre-onset*	**↑↑↑**	**↑↑↑**
		*Post-onset*	**↑↑↑** | **↓** | **↓↓**	**↑** | **↓↓** | **↓↓↓**
SMA (SMA / premotor cortex, BA6)	*Motor planning / initiation*	*Pre-onset*	**↑↑↑**	**↑↑↑**
		*Post-onset*	**↑↑↑** | **↓↓↓** | **↓↓↓**	**↑** | **↓↓↓** | **↓↓↓**
S1 (primary somatosensory cortex, BA1–3)	*Tactile / proprioceptive processing*	*Pre-onset*	**↑↑↑**	**↑↑**
		*Post-onset*	**↑↑↑** | **↓** | **↓↓↓**	- | **↓↓↓** | **↓↓↓**
Insula (insula, BA13)	*Interoception / homeostatic integration*	*Pre-onset*	**↑↑↑**	**↑↑↑**
		*Post-onset*	**↑↑↑** | **↑↑↑** | **↑↑↑**	**↑** | **↑** | **↑↑**
LT (lateral temporal cortex (STG/MTG), BA21/22)	*Auditory / language processing*	*Pre-onset*	**↑↑**	**↑↑**
		*Post-onset*	**↑↑↑** | **↑↑↑** | **↑↑**	**↑** | **↑↑** | **↑↑↑**
Occ (occipital visual cortex (V1–V3), BA17–19)	*Visual processing*	*Pre-onset*	**↑**	↑↑*
		*Post-onset*	**↑↑↑** | **↓↓↓** | **↓↓↓**	- | **↓↓↓** | **↓↓↓**

Arrows indicate the proportion of significant cortical vertices within each region: one arrow, weak effect (<50% of vertices); two arrows, moderate effect (50%–80%); three arrows, strong effect (>80%). Upward arrows denote increases and downward arrows denote decreases. For post-onset analyses, symbols refer to three consecutive 5-s windows after movement onset (0–5 s, 5–10 s, and 10–15 s). A dash (–) indicates no significant effect or unavailable data

In the beta band, bilateral power increases were already present before onset, involving the DLPFC, VLPFC, and dmPFC, and to a lesser extent, M1, S1, and the paracentral gyri, as well as the SMA, temporopolar cortex, and superior temporal regions. In contrast, the occipital pole and inferior occipital, temporal and frontal surfaces (Figure 1B, Table 2, Table S6) were largely spared. Beta activity remained significantly elevated throughout the 15 s post-onset window, with a stable, widespread bilateral distribution.

**Table 2:** Beta-band (18–30 Hz) source-space changes during DoA episodes relative to baseline SWS in children and adults

Brain region	Functional role	Phase	Children	Adults
ACC (anterior cingulate cortex, BA24/32)	*Salience / conflict monitoring*	*Pre-onset*	**↑↑**	**↑↑↑**
		*Post-onset*	**↑↑↑** | **↑↑↑** | **↑↑↑**	**↑↑↑** | **↑↑↑** | **↑↑↑**
PCC (posterior cingulate cortex, BA23/31)	*Default mode / self-referential*	*Pre-onset*	**↑↑↑**	**↑↑↑**
		*Post-onset*	**↑↑↑** | **↑↑↑** | **↑↑↑**	**↑↑↑** | **↑↑↑** | **↑↑↑**
mPFC (medial prefrontal cortex, BA10/32)	*Default mode / self-regulation*	*Pre-onset*	**↑↑↑**	**↑↑↑**
		*Post-onset*	**↑↑↑** | **↑↑↑** | **↑↑↑**	**↑↑↑** | **↑↑↑** | **↑↑↑**
DLPFC (dorsolateral prefrontal cortex, BA9/46)	*Executive control*	*Pre-onset*	**↑↑**	**↑↑↑**
		*Post-onset*	**↑↑↑** | **↑↑↑** | **↑↑↑**	**↑↑↑** | **↑↑↑** | **↑↑↑**
FPC (frontopolar cortex, BA10)	*Prospective memory / integration*	*Pre-onset*	**↑↑↑**	**↑↑↑**
		*Post-onset*	**↑↑↑** | **↑↑↑** | **↑↑↑**	**↑↑↑** | **↑↑↑** | **↑↑↑**
OFC (orbitofrontal cortex, BA11/12)	*Reward / affective valuation*	*Pre-onset*	**↑↑↑**	**↑↑↑**
		*Post-onset*	**↑↑↑** | **↑↑↑** | **↑↑↑**	**↑↑↑** | **↑↑↑** | **↑↑↑**
Precuneus (precuneus, BA7/31)	*Default mode / episodic memory*	*Pre-onset*	**↑↑↑**	**↑↑↑**
		*Post-onset*	**↑↑↑** | **↑↑↑** | **↑↑↑**	**↑↑↑** | **↑↑↑** | **↑↑↑**
SPL (superior parietal lobule, BA7)	*Visuospatial integration*	*Pre-onset*	**↑↑**	**↑↑↑**
		*Post-onset*	**↑↑↑** | **↑↑↑** | **↑↑↑**	**↑↑↑** | **↑↑↑** | **↑↑↑**
TPJ-L (left temporoparietal junction, BA39/40/22)	*Social cognition / attention*	*Pre-onset*	**↑**	**↑↑↑**
		*Post-onset*	**↑↑↑** | **↑↑↑** | **↑↑↑**	**↑↑↑** | **↑↑↑** | **↑↑↑**
TPJ-R (right temporoparietal junction, BA39/40/22)	*Social cognition / attention*	*Pre-onset*	**↑↑↑**	**↑↑↑**
		*Post-onset*	**↑↑↑** | **↑↑↑** | **↑↑↑**	**↑↑↑** | **↑↑↑** | **↑↑↑**
M1 (primary motor cortex, BA4)	*Voluntary movement execution*	*Pre-onset*	**↑↑**	**↑↑↑**
		*Post-onset*	**↑↑↑** | **↑↑↑** | **↑↑↑**	**↑↑↑** | **↑↑↑** | **↑↑↑**
SMA (SMA / premotor cortex, BA6)	*Motor planning / initiation*	*Pre-onset*	**↑↑**	**↑↑↑**
		*Post-onset*	**↑↑↑** | **↑↑↑** | **↑↑↑**	**↑↑↑** | **↑↑↑** | **↑↑↑**
S1 (primary somatosensory cortex, BA1–3)	*Tactile / proprioceptive processing*	*Pre-onset*	**↑↑**	**↑↑↑**
		*Post-onset*	**↑↑↑** | **↑↑↑** | **↑↑↑**	**↑↑↑** | **↑↑↑** | **↑↑↑**
Insula (insula, BA13)	*Interoception / homeostatic integration*	*Pre-onset*	**↑↑↑**	**↑↑↑**
		*Post-onset*	**↑↑↑** | **↑↑↑** | **↑↑↑**	**↑↑↑** | **↑↑↑** | **↑↑↑**
LT (lateral temporal cortex (STG/MTG), BA21/22)	*Auditory / language processing*	*Pre-onset*	**↑↑↑**	**↑↑↑**
		*Post-onset*	**↑↑↑** | **↑↑↑** | **↑↑↑**	**↑↑↑** | **↑↑↑** | **↑↑↑**
Occ (occipital visual cortex (V1–V3), BA17–19)	*Primary/secondary visual processing*	*Pre-onset*	**↑↑↑**	**↑**
		*Post-onset*	**↑↑↑** | **↑↑↑** | **↑↑↑**	**↑↑↑** | **↑↑↑** | **↑↑↑**

Arrows indicate the proportion of significant cortical vertices within each region: one arrow, weak effect (<50% of vertices); two arrows, moderate effect (50%–80%); three arrows, strong effect (>80%). Upward arrows denote increases and downward arrows denote decreases. For post-onset analyses, symbols refer to three consecutive 5-s windows after movement onset (0–5 s, 5–10 s, and 10–15 s). A dash (–) indicates no significant effect or unavailable data

### Spectral power spatial dynamics from SWS to NREM parasomnia episodes in children

**Figure 2:**
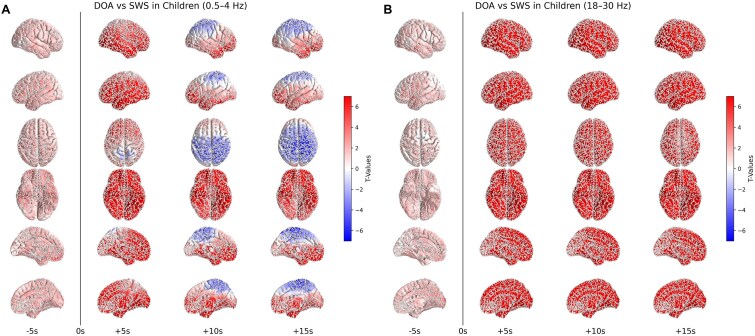
Spatiotemporal spectral dynamics around motor onset during DoA episodes in children. (A) Source-level t-maps of delta power (0.5–4 Hz) show differences between DoA events and stable slow-wave sleep (SWS) baseline (−3 to −2 min), across four windows from −5 to +15 seconds relative to movement onset (0 s). Warmer colors (red) indicate increased power; cooler (blue) indicate decreased power during parasomnia. In children, early widespread delta increase is observed at −5 s, followed by a fronto-parietal suppression from +10 s onward. Compared to adults (Figure 1A), the delta increase appears more diffuse and sustained, while the later suppression is more spatially confined. (B) Source-level t-maps of beta power (18–30 Hz) reveal sustained and bilateral increases in activity throughout the same windows, relative to SWS baseline. Compared to adults (Figure 1B), children show a similarly widespread and persistent beta enhancement across all time points. White dots indicate scouts with statistically significant differences (pTFCE-corrected). All maps are thresholded by corrected t-values.

In children, source-level analysis showed a robust, spatially organized delta increase relative to SWS with left-hemispheric dominance. As in adults, the right posterior superior temporal cortex and the occipital regions did not reach significance (Figure 2A, Table 1, Table S7). In the first 5 s after onset, the delta build-up was broader than in adults, engaging frontal, temporal, occipital, cingulate and insular cortices, with particularly strong bilateral activity in OFC and FPC, ACC/mid-anterior cingulate, inferior/middle frontal gyri, insula and subcallosal areas. By contrast, a midline–sensorimotor cluster centered on the precuneus, SPL and para-central gyri, appeared relatively spared immediately post-onset (negative but non-significant). This cluster became significantly suppressed between 5–15 s post-onset, with additional effects particularly in the MCC/PCC and sensorimotor regions (S1–M1, SMA).

In the −5 to 0 s window, beta increases were less extensive than in adults, peaking in M1 (extending mainly into the left subcentral area) and spreading to the DLPFC (particularly the left inferior frontal gyrus), as well as bilateral OFC regions, the temporopolar cortex and parts of the lateral temporal surface (left > right) (Figure 2B, Table 2, Table S7). Post-onset, as in adults, beta activity was substantial and widespread throughout 0–15 s.The group × condition interaction analysis confirmed that the spatial extent of delta enhancement during DoA episodes was significantly greater in children than in adults.

### Spectral power spatial differences surrounding NREM parasomnia episodes versus cortical motor arousals in children and adults

**Figure 3:**
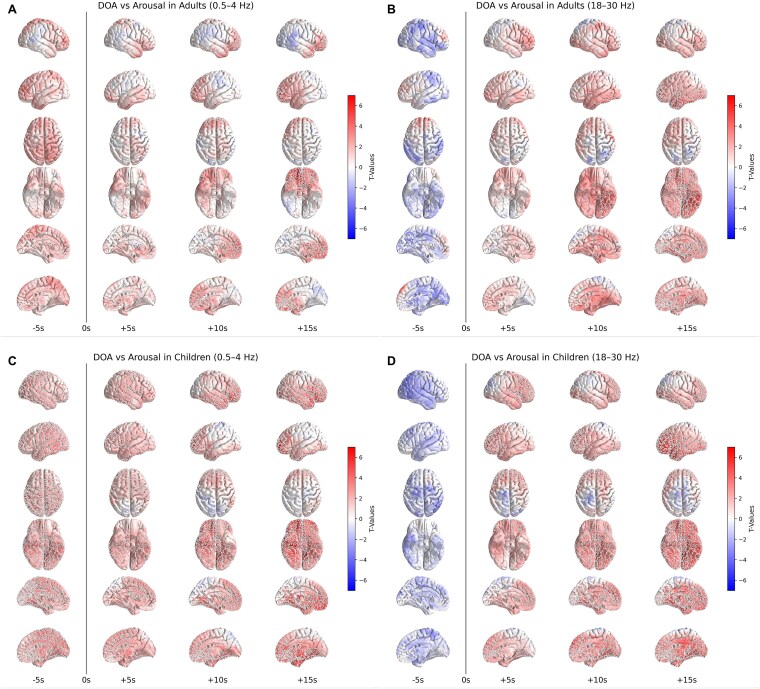
Comparison of spectral power between DoA events and typical motor arousals in adults and children. Source-level t-maps display spectral power differences between DoA episodes and typical motor arousals during the −5 to +15 second window relative to movement onset (0 s). Warmer colors (red) indicate higher power in parasomnia events compared to typical arousals; cooler colors (blue) indicate lower power. White dots mark vertices within scouts showing statistically significant differences (pTFCE-corrected t-values). (A) Delta power (0.5–4 Hz) in adults. Before movement onset, DoA events show higher delta activity, particularly in frontal and midline parietal regions. From 0 to +5 seconds, this difference becomes statistically significant over fronto-polar and fronto-basal surfaces. (B) Beta power (18–30 Hz) in adults. In the −5 to 0 second window, beta activity is lower during DoA events, especially over centro-posterior regions, but increases after motor onset, becoming significant between +5 and + 10 seconds over ventral brain regions. (C) Delta power (0.5–4 Hz) in children. As in adults, DoA events in children are preceded by increased delta activity, which is widespread during the −5 to 0 second window and becomes confined to anterior brain regions post-onset, mainly the frontal cortex, anterior cingulate, and temporopolar areas. (D) Beta power (18–30 Hz) in children. Like adults, children show reduced beta activity during DoA events before onset and significantly higher beta during episodes, especially over ventral, frontal, and temporopolar brain regions.

In both children and adults, delta power in the −5 to 0 s window was higher during DoA than physiological motor arousals, reaching statistical significance only in children, with the strongest effects over posterior cortices (Figure 3A and C; Table 3; Tables S8–S9). Beta power in the same window tended to be lower in DoA across similar regions, but differences were not significant after automatic cleaning (Figure 3B and D; Table 4; Tables S8–S9).

**Table 3:** Delta-band (0.5–4 Hz) source-space changes during DoA episodes relative to physiological motor arousals in children and adults

Brain region	Functional role	Phase	Children	Adults
ACC (anterior cingulate cortex, BA24/32)	*Salience / conflict monitoring*	*Pre-onset*	**↑↑↑**	-
		*Post-onset*	**↑↑↑** | **↑↑↑** | **↑↑↑**	- | **↑↑↑** | **↑↑**
PCC (posterior cingulate cortex, BA23/31)	*Default mode / self-referential*	*Pre-onset*	**↑↑↑**	-
		*Post-onset*	**↑** | **↑↑** | **↑↑**	- | - | -
mPFC (medial prefrontal cortex, BA10/32)	*Default mode / self-regulation*	*Pre-onset*	**↑↑**	-
		*Post-onset*	**↑↑** | **↑↑** | **↑↑↑**	- | **↑↑** | **↑↑↑**
DLPFC (dorsolateral prefrontal cortex, BA9/46)	*Executive control*	*Pre-onset*	**↑↑**	-
		*Post-onset*	**↑↑** | **↑↑↑** | **↑↑↑**	- | **↑↑** | **↑↑**
FPC (frontopolar cortex, BA10)	*Prospective memory / integration*	*Pre-onset*	**↑↑**	-
		*Post-onset*	**↑** | **↑↑↑** | **↑↑↑**	- | **↑↑** | **↑↑↑**
OFC (orbitofrontal cortex, BA11/12)	*Reward / affective valuation*	*Pre-onset*	**↑↑↑**	-
		*Post-onset*	**↑** | **↑↑↑** | **↑↑↑**	- | **↑↑** | **↑↑↑**
Precuneus (precuneus, BA7/31)	*Default mode / episodic memory*	*Pre-onset*	**↑↑↑**	-
		*Post-onset*	- | - | **↑**	- | - | -
SPL (superior parietal lobule, BA7)	*Visuospatial integration*	*Pre-onset*	**↑↑↑**	-
		*Post-onset*	**↑** | - | -	- | - | -
TPJ-L (left temporoparietal junction, BA39/40/22)	*Social cognition / attention*	*Pre-onset*	**↑↑↑**	-
		*Post-onset*	**↑↑** | **↑** | **↑↑**	- | - | -
TPJ-R (right temporoparietal junction, BA39/40/22)	*Social cognition / attention*	*Pre-onset*	**↑↑↑**	-
		*Post-onset*	**↑↑↑** | **↑↑↑** | **↑↑↑**	- | **↑** | **↑↑**
M1 (primary motor cortex, BA4)	*Voluntary movement execution*	*Pre-onset*	**↑↑**	-
		*Post-onset*	**↑** | **↑** | **↑**	- | - | -
SMA (SMA / premotor cortex, BA6)	*Motor planning / initiation*	*Pre-onset*	**↑↑↑**	-
		*Post-onset*	**↑** | **↑** | **↑**	- | - | **↑**
S1 (primary somatosensory cortex, BA1–3)	*Tactile / proprioceptive processing*	*Pre-onset*	**↑↑↑**	-
		*Post-onset*	**↑↑** | - | **↑**	- | - | -
Insula (insula, BA13)	*Interoception / homeostatic integration*	*Pre-onset*	**↑↑↑**	-
		*Post-onset*	**↑↑** | **↑↑↑** | **↑↑↑**	- | **↑** | **↑↑**
LT (lateral temporal cortex (STG/MTG), BA21/22)	*Auditory / language processing*	*Pre-onset*	**↑↑↑**	-
		*Post-onset*	**↑↑** | **↑↑** | **↑↑↑**	- | **↑** | **↑**
Occ (occipital visual cortex (V1–V3), BA17–19)	*Primary/secondary visual processing*	*Pre-onset*	**↑↑↑**	-
		*Post-onset*	**↑** | **↑↑** | **↑↑↑**	- | - | -

Arrows indicate the proportion of significant cortical vertices within each region: one arrow, weak effect (<50% of vertices); two arrows, moderate effect (50%–80%); three arrows, strong effect (>80%). Upward arrows denote increases and downward arrows denote decreases. For post-onset analyses, symbols refer to three consecutive 5-s windows after movement onset (0–5 s, 5–10 s, and 10–15 s). A dash (–) indicates no significant effect or unavailable data

**Table 4:** Beta-band (18–30 Hz) source-space changes during DoA episodes relative to physiological motor arousals in children and adults

Brain region	Functional role	Phase	Children	Adults
ACC (anterior cingulate cortex, BA24/32)	*Salience / conflict monitoring*	*Post-onset*	**↑↑↑** | **↑↑↑** | **↑↑↑**	- | - | **↑↑↑**
PCC (posterior cingulate cortex, BA23/31)	*Default mode / self-referential*	*Post-onset*	- | **↑↑↑** | **↑↑↑**	- | - | **↑↑↑**
mPFC (medial prefrontal cortex, BA10/32)	*Default mode / self-regulation*	*Post-onset*	**↑↑↑** | **↑↑↑** | **↑↑↑**	- | - | **↑↑↑**
DLPFC (dorsolateral prefrontal cortex, BA9/46)	*Executive control*	*Post-onset*	**↑↑↑** | **↑↑↑** | **↑↑↑**	- | - | **↑↑**
FPC (frontopolar cortex, BA10)	*Prospective memory / integration*	*Post-onset*	**↑↑↑** | **↑↑↑** | **↑↑↑**	- | - | **↑↑↑**
OFC (orbitofrontal cortex, BA11/12)	*Reward / affective valuation*	*Post-onset*	**↑↑↑** | **↑↑↑** | **↑↑↑**	- | - | **↑↑↑**
Precuneus (precuneus, BA7/31)	*Default mode / episodic memory*	*Post-onset*	- | **↑↑** | **↑**	- | - | **↑**
SPL (superior parietal lobule, BA7)	*Visuospatial integration*	*Post-onset*	- | **↑** | **↑**	- | - | -
TPJ-L (left temporoparietal junction, BA39/40/22)	*Social cognition / attention*	*Post-onset*	**↑** | **↑↑** | **↑↑↑**	- | - | **↑↑↑**
TPJ-R (right temporoparietal junction, BA39/40/22)	*Social cognition / attention*	*Post-onset*	**↑** | **↑** | **↑↑**	- | - | **↑↑**
M1 (primary motor cortex, BA4)	*Voluntary movement execution*	*Post-onset*	**↑** | **↑** | **↑**	- | - | **↑**
SMA (SMA / premotor cortex, BA6)	*Motor planning / initiation*	*Post-onset*	**↑↑↑** | **↑↑** | **↑↑↑**	- | - | **↑**
S1 (primary somatosensory cortex, BA1–3)	*Tactile / proprioceptive processing*	*Post-onset*	**↑** | - | **↑**	- | - | **↑**
Insula (insula, BA13)	*Interoception / homeostatic integration*	*Post-onset*	**↑↑↑** | **↑↑↑** | **↑↑↑**	- | - | **↑↑↑**
LT (lateral temporal cortex (STG/MTG), BA21/22)	*Auditory / language processing*	*Post-onset*	**↑** | **↑↑** | **↑↑↑**	- | **↑** | **↑↑↑**
Occ (occipital visual cortex (V1–V3), BA17–19)	*Primary/secondary visual processing*	*Post-onset*	- | **↑↑↑** | **↑↑↑**	- | **↑** | **↑↑↑**

Arrows indicate the proportion of significant cortical vertices within each region: one arrow, weak effect (<50% of vertices); two arrows, moderate effect (50%–80%); three arrows, strong effect (>80%). Upward arrows denote increases and downward arrows denote decreases. For post-onset analyses, symbols refer to three consecutive 5-s windows after movement onset (0–5 s, 5–10 s, and 10–15 s). A dash (–) indicates no significant effect or unavailable data

Following onset, delta power remained elevated in DoA. In children, this effect was widespread, with positive clusters in the insula, cingulate (particularly the ACC), frontal regions (including the dmPFC/vmPFC, FPC, and OFC), and ventral cortical areas, while sparing the centro-posterior zone described above. Adults showed a similar but more anteriorly focused pattern (ACC, OFC, vmPFC), with the strongest effects observed between 5–15 s post-onset (Figure 3A and C; Table 3, Tables S8–S9).

Post-onset beta power was significantly higher in DoA than physiological motor arousals, with an early diffuse rise followed by more focal frontal anterior and ventral enhancements. This effect was robust in children, especially over PFC, OFC, ACC, temporopolar, insular, ventrotemporal, and occipital regions. In adults, significant clusters were fewer and appeared mainly between 5–15 s post-onset (Figure 3B and D; Table 4, Tables S8–S9).

No significant group × condition interaction was observed when comparing DoA with physiological motor arousals, indicating that the two age groups share a comparable pattern of spectral differentiation from physiological events.

## Discussion

Using high-density EEG source modeling, we identified four reproducible cortical hallmarks of DoA, providing the most spatially and temporally detailed characterization to date of cortical oscillatory dynamics during DoA, resolved in 5-second windows, spanning 5 seconds before to 15 seconds after movement onset. By studying both children and adults with DoA and directly contrasting episodes against stable SWS and physiological motor arousals, we establish a quantitative, anatomically precise framework for mechanistic interpretation.

First, in the seconds immediately preceding a DoA episode, we observed a pronounced delta increase relative to stable SWS in both children and adults, mainly encompassing FPC, OFC, ACC, MCC, temporo-polar, left TPJ, and M1-S1. Concurrent beta increases engaged similar motor-associative networks. These changes expand on earlier low-resolution EEG studies [15, 18–24] by providing a detailed topographical characterization and revealing qualitative similarities with physiological arousals [38], which suggest a common initiation arousal mechanism. However, when directly compared with physiological motor arousals, DoA episodes tended to arise from a globally more “sleep-like” background, with higher delta and lower beta power. This pattern was more pronounced in children and is consistent with previous adult findings [16, 39].

Second, a defining feature of the post-onset period in both age groups was the sustained delta power increase over frontal and prefrontal cortices, most prominently in areas involved in prospective memory and high-level goal integration (FPC), reward and affective valuation (OFC), and the integration of emotional and value-based information for decision-making (vmPFC). Critically, this frontal delta persistence represents a failure of these higher-order regions to transition towards wake-like activity despite ongoing motor behavior. When compared to physiological arousals, the same regions showed significantly greater delta in DoA during the post-onset period in both groups, confirming the specificity of this frontal delta “lock” to the parasomnia state. This frontal configuration is inconsistent with the normal arousal trajectory during physiological awakenings and provides a neurophysiological substrate for the impaired environmental responsiveness, attenuated executive function, and disoriented behavior characteristic of DoA.

Third, in parallel with frontal delta persistence, DoA episodes were marked by a progressive delta power suppression over a distinct centro-parietal cortical cluster, encompassing a set of regions supporting default-mode self-referential processing, visuospatial integration [40], episodic memory and conscious experiences during dreaming [41] (PCC, precuneus, SPL), somatomotor execution and proprioceptive processing (M1, SMA, S1). This suppression emerged within 5 seconds of movement onset, intensified over the 5–15 second post-onset window, and was present in both children and adults.

The simultaneous occurrence of delta suppression (wake-like reactivation) in this cluster and delta persistence (sleep-like state) in frontal regions constitutes the core cortical dissociation defining DoA: sensorimotor and arousal/consciousness related networks partially reactivate and support the motor behavioral output, while prefrontal and cingulate networks remain in a sleep-like or mixed state and fail to support environmental integration and behavioral regulation. Quantitative age differences in the magnitude and timing of this suppression were observed: adults showed significantly stronger precuneus and parieto-occipital delta suppression relative to children. Children exhibited a delayed trajectory, with some parietal regions showing initial delta increases before transitioning to suppression in the 5–15 second window. This attenuation of parietal default-mode deactivation in children is consistent with, albeit on limited evidence [3, 42], the view that conscious mental activity is less frequent in pediatric DoA than adult episodes.

Of note, the described cluster of delta suppression closely resembles that reported by Cataldi et al. [17] when contrasting episodes with and without conscious experiences, consistent with their finding that the former are more frequent in adults. However, our regional delta dynamics partially diverge from Cataldi et al. [16], who found a global delta reduction during adult DoA episodes and less consistent, less localized post-onset effects. This discrepancy likely reflects methodological differences: we analyzed only baseline-night arousals, applied more conservative artifact rejection (manual channel/epoch cleaning plus milder automatic procedures), and performed source-space analyses on normalized data using mixed-effects modeling. The fourth and last hallmark is a sustained, bilateral widespread beta power increase that was present across the entire peri-onset period in both children and adults. This increase was widespread, encompassingp refrontal, cingulate, temporal, parietal, insular, and occipital cortices, with most regions showing increases throughout all time windows and high spatial comparability across age groups. Compared to physiological motor arousals, post-onset beta power was significantly higher in children, whereas in adults a more temporally restricted increase, primarily involving frontotemporal and anterior cingulate regions, emerged mainly within the 10–15 second window.

A central finding of this study is that the four hallmarks described above are largely conserved across age groups. The consistency of our patterns across children and adults, and their close replication of previous findings from a pediatric case report [43] and SEEG studies (see Tables S810), supports the robustness and reproducibility of these spatiotemporal dynamics. This cross-age continuity also provides strong evidence for a shared, stable core neurophysiological mechanism, rather than age-specific or developmentally distinct mechanisms. This cross-age concordance extends prior models of DoA that were almost exclusively derived from adult neurophysiological data and supports the view that the neurophysiological machinery underlying DoA matures early or is robustly expressed across developmental stages. The persistence of episodes into adulthood may reflect a greater genetic predisposition and/or, as shown by several studies, the continued presence or emergence of predisposing or triggering factors, such as stress or psychopathology [4].

Taken together, our findings confirm that, while local sleep–wake dissociation is an adaptive NREM feature, allowing brief motor adjustments without full awakening, it persists abnormally during DoA. Concurrent increases in low- and high-frequency across distinct, though partially overlapping, cortical regions [10], mirror SEEG findings, in which wake-like motor cortex activation was preceded by and accompanied by frontal delta bursts during both subtle and overt movements [44]. This functional dissociation between motor-control and executive networks is a core defining feature of DoA that enables complex motor acts with diminished environmental responsiveness.

More importantly, our results support a revised mechanistic model in which DoA represent a failure not of arousal initiation per se but of arousal integration. Before motor onset, differences between DoA and physiological motor arousals are primarily quantitative and global rather than qualitative and regional, supporting the view that DoA often occur during predisposing time windows when cortical activation is insufficient to support full wakefulness. Depending on cortical state (e.g. a “sleepier” background shaped by NREM depth), the strength and pattern of subcortical arousal activation, and individual predisposition, arousal may resolve spontaneously, transition to full wakefulness, or escalate into a DoA episode. What distinguishes DoA from physiological arousals is not the initiation of arousal, but the failure of this arousal signal to propagate in a spatially coordinated manner across the full cortical hierarchy and to engage key regions. Instead of achieving the broad, sensorimotor and subsequent frontal-to-posterior arousal gradient that characterizes normal awakening, DoA are marked by an arrested propagation: sensorimotor and parietal networks partially reactivate while prefrontal and anterior cingulate regions, needed for full awakening and behavioral regulation, remain in a sleep-like state. Thus, DoA may represent a breakdown in arousal synchronization across cortical subsystems, producing partial awakenings in which sensorimotor circuits reactivate while higher-order frontal networks remain offline.

We speculate that this cortical-level dissociation may arise from altered activity in the wake-promoting noradrenergic system [45], whose modular organization could differentially support motor and prefrontal circuits [46, 47]. A plausible account is that during DoA, Locus Coeruleus (LC) firing patterns preferentially engage motor cortex projections while failing to adequately activate prefrontal targets, producing the spatial dissociation observed in the oscillatory data. Whether this reflects a constitutive difference in LC output organization, a state-dependent modulation of noradrenergic tone, or a downstream consequence of impaired thalamocortical propagation cannot be determined from the current data and represents a testable hypothesis for future work. Similarly, the impaired progression of thalamic arousal [48] from early centro-median nuclei (such as the ventral intermediate nucleus active during DoA [13]), supporting core arousal [49], to later integrative nuclei (such as the Pulvinar, relatively inactive in DoA [10]) may represent a thalamic-level correlate of the arrested cortical propagation observed here. These latter nuclei mediate integrative motor, cognitive, and sensory functions [50], and their recruitment depends on synchronized activation of associative cortical areas, typically occurring only after diffuse arousal nuclei and frontal regions are engaged [51]. In DoA, the process may stall before this stage, due to an impaired frontal activation, preventing full awakening of late thalamic hubs and of higher-order cortical networks.

In addition to improving our understanding of the underlying mechanisms of DoA, the identification of age-invariant, anatomically precise cortical hallmarks of DoA have relevant clinical implications, positioning hd-EEG as a translational tool for neurology and sleep medicine. Such EEG signatures may assist in the differential diagnosis with sleep-related hypermotor epilepsy (SHE) and other nocturnal events that often present with overlapping semiology but require very different management strategies [2, 8]. These markers could in future also help stratify patients at higher risk of injuries, psychosocial distress, or forensic consequences [5]. Moreover, the pre-onset spectral changes identified here in the 5 seconds preceding motor onset opens avenues for prospective investigation with larger samples and real-time EEG classification approaches. If this effecy is sufficiently specific and temporally extended to be exploited for predictive purposes requires, closed-loop stimulation strategies could be used in the future to detect fragile EEG windows in which DoA episodes can be experimentally induced for diagnostic purposes or interrupted before full episode expression.

Limitations of this study include grouping multiple parasomnia subtypes (most events were confusional arousals), and using template MRIs for source modeling, which may slightly reduce spatial precision. Moreover, as all participants were recruited from a specialized sleep medicine center, the sample may be biased toward more severe or treatment-seeking cases, potentially limiting applicability to milder presentations. However, the high reproducibility across ages and convergence with SEEG case reports support the robustness of our patterns.

Future studies with larger, prospectively recruited samples, including patients with multiple DoA subtypes should clarify whether cortical dynamics differ among confusional arousals, sleepwalking, and night terrors to strengthen generalizability. The comparison with patients with SHE is needed to determine whether the hallmarks identified here are specific to DoA or shared with epileptic nocturnal motor events. The use of individual MRI data will improve source localization precision, particularly for regions implicated in the core dissociation. Integrating hd-EEG with concurrent phenomenological assessments in children will enable direct and precise examination of the relationship between centro-parietal suppression dynamics and the presence or absence of conscious mental activity during episodes.

## Conclusions

To conclude, our source mapping analysis uncovered four reproducible cortical hallmarks that define DoA across the lifespan: (1) a globally sleep-like pre-episode brain state, (2) persistent post-onset frontal delta enhancement as a marker of failed prefrontal engagement, (3) concurrent centro-parietal delta suppression reflecting partial motor-sensorimotor reactivation, and (4) bilateral beta enhancement throughout the peri-onset period. Critically, these cortical signatures are broadly conserved between children and adults, suggesting a shared core pathophysiology rather than age-specific mechanisms. Together, these hallmarks support a model in which DoA arise from a breakdown in the spatial integration of arousal across cortical subsystems, producing dissociated sleep-like and wake-like activity in anatomically and functionally distinct networks. The growing recognition of the modularity of arousal systems, at both modulatory neurotransmitter and thalamocortical levels, could guide the development of new, testable hypotheses based on the precise topographical patterns identified here, advancing the understanding of DoA pathophysiology and informing novel pharmacotherapeutic approaches. Furthermore, the identification of EEG changes that precede movement onset may enable future efforts to predict or modulate episodes through spectral EEG dynamics, support early intervention or preventive strategies in high-risk individuals, and improve differential diagnosis with SHE when only minor episodes are captured in the laboratory.

## Supplementary Material

supplementary_materials_Z2_zsag123

## Data Availability

The datasets generated during and/or analyzed during the current study are available from the corresponding author on reasonable request. Additional videos can be accessed upon direct request to the corresponding author, in accordance with patient consent. Templates of the custom-made codes in Matlab are available at https://github.com/julama/hdEEG_DOA.
