# Supplementary material for: Shared local brain dynamics in pediatric and adult non-rapid eye movement parasomnias
Source: Sleep. 2026 May 8;49(7):zsag123. doi: 10.1093/sleep/zsag123 (PMC13357507; doi:10.1093/sleep/zsag123)

**Shared local brain dynamics in pediatric and adult NREM parasomnias**

Julian Amacker^1†^, Marco Veneruso^2,3,4†^, Matteo Pereno^3^, Simone Ulzega^1^, Samuel Wehrli^1^, Sven Hirsch^1^, Lino Nobili^2,4^, Silvia Miano^3,5^, Mauro Manconi^3,5,6^, Anna Castelnovo^3,5,7^

**^†^These authors contributed equally to this work.**

**Author affiliations:**

1 Institute of Computational Life Sciences, Zurich University of Applied Sciences, 8820 Wädenswil, Switzerland

2 Department of Neurosciences, Rehabilitation, Ophthalmology, Genetics, Maternal and Child Health (DINOGMI), University of Genova, 16132 Genova, Italy

3 Sleep Medicine, Neurocenter of Southern Switzerland, Ospedale Civico, 6900 Lugano, Switzerland

4 Child Neuropsychiatry Unit, IRCCS Istituto Giannina Gaslini, 16147 Genova, Italy

5 Faculty of Biomedical Sciences, University of Italian Switzerland, 6900 Lugano, Switzerland

6 Department of Neurology, University Hospital, Inselspital, 3010 Bern, Switzerland

7 University Hospital of Psychiatry and Psychotherapy, University of Bern, 3000 Bern, Switzerland.

**Correspondence to**: Anna Castelnovo

Via Tesserete 46, 6900 Lugano

[anna.castelnovo@usi.ch](mailto:anna.castelnovo@usi.ch)

| **ID** | **DoA Subtype** | **Family history** | **Frequency** | **Medications** | **Sleep comorbidities** | **Other major comorbidities** |
| --- | --- | --- | --- | --- | --- | --- |
| **A1** | CA | Yes | >1/week | None | Mild chronic sleep deprivation | None |
| **A2** | SW | No | >1/week | None | Mild OSA | None |
| **A3** | SW, SRSD | No | 1/month | None | Sleeptalking, bruxism | GERD, Crohn's disease |
| **A4** | SW ST, SRSD | Yes | >1/week | None | Sleeptalking, hypnagogic hallucinations | None |
| **A5** | SW, ST | Yes | >1/month | None | Sleeptalking, hypnagogic hallucinations | None |
| **A6** | SW, ST | Yes | 1/month | Estro-Progestinic | Sleeptalking, hypnagogic hallucinations | Headache |
| **A7** | SW | Unknown | >1/week | Bisoprolol 2.5 mg (**) | Moderate OSA, sleeptalking, hypnagogic hallucinations | Hypertension |
| **A8** | CA | Yes | >1/year | None | None | None |
| **A9** | SW, CA | No | clusters | None | None | Migraine |
| **A10** | SW, ST | Yes | >1/week | L-Thyroxine 100 mcg, Estro-Progestinic | None | Hypotyroidism (thyroid agenesis) |
| **A11** | SW | Yes | >1/week | Zolpidem 2.5 mg as needed -on average 1/month (***) | Mild insomnia | None |
| **A12** | CA | Yes | >1/week | Estro-Progestinic | Mild insomnia | None |
| **A13** | SW | Yes | >1/month | None | Bruxism | None |
| **A14** | SW | Yes | 1/month | None | None | GERD |
| **A15** | ST | Yes | >1/week | Estro-Progestinic | None | Migraine |
| **A16** | CA | Unknown | clusters | L-Thyroxine 125 mcg, Estro-Progestinic | None | Hypotyroidism, |
| **A17** | CA | Yes | >1/week | L-Thyroxine 150/175 mcg | Bruxism, mild OSA | Hypotyroidism |
| **A18** | SW, ST | No | 1/month | None | Moderate OSA | None |
| **A19** | SW, CA | Yes | everyday | None | None | None |
| **A20** | CA | No | >1/month | None | Mild OSA | None |
| **A21** | SW | No | >1/week | Erenumab 70 mg (**) | None | Migraine |
| **A22** | SW, ST, CA | Yes | >1/week | None | Mild insomnia | None |

**Table S1. Clinical and Demographic Characteristics of Adult DoA Participants**

ID – Participant code; DoA: Disorders of Arousal, Subtype – CA = Confusional arousals, SW = Sleepwalking, ST = Sleep terrors, SRSD = Sleep-related sexual disorder; Family history – Presence of the disorder in family (Yes, No, Unknown); Frequency – Episode occurrence rate; Medications – Current treatments at the time of enrollment; Sleep comorbidities – Other diagnosed sleep disorders (OSA = obstructive sleep apnea); Other major comorbidities – Other physical health conditions (GERD = gastroesophageal reflux disease). (*) Uncertain episodes during childhood; (**) Medication withheld on the day of recording; discontinued three days prior; (***) One-month washout period. Note: A7 and A18 were excluded due to the finding of moderate OSA.

| **ID** | **DoA Subtype** | **Family history** | **Frequency** | **Medications** | **Sleep comorbidities** | **Other major comorbidities** |
| --- | --- | --- | --- | --- | --- | --- |
| **C1** | SW | Yes | 1/week | Melatonin 1 mg (*) | Mild insomnia | Separation anxiety |
| **C2** | SW, NT | Yes | >1/week | None | Sleep hyperhydrosis | None |
| **C3** | SW | Yes | >1/month | Salbutamol spray as needed (*) | None | Orchiopexy, amelanogenesis imperfecta, separation anxiety |
| **C4** | CA | Yes | >1/month | None | Sleeptalking, bruxism, mild insomnia | Talaxemia minor, tics, needle phobia |
| **C5** | SW, CA, NT | Yes | >1/week | None | None | None |
| **C6** | SW | No | Everyday | None | None | None |
| **C7** | SW | Yes | >1/week | None | Mild insomnia, sleeptalking, sleep hallucinations | Asthma, mild compulsive symptoms |
| **C8** | SW | Yes | >1/week | None | None | Asthma, epileptic abnormalities |
| **C9** | NT, SW | Yes | >1/month | None | None | None |
| **C10** | NT, CA | Yes | Everyday | None | None | None |
| **C11** | SW, CA | No | >1/week | None | Severe OSA, bruxism, bleeptalking | None |
| **C12** | SW, NT | Yes | Everyday | None | Flow-limitation | None |
| **C13** | SW, CA | No | >1/week | Laxatives as needed | Mild insomnia, sleep hyperhydrosis | Patent foramen ovale, autism spectrum disorder – level 1 |
| **C14** | SW | No | >1/week | None | Bruxism | Primary exercise headache |
| **C15** | SW | Yes | Everyday | Metylphenidate 10 mg (*) | Sleep hyperhydrosis | ADHD, Rolandic epilepsy (past) |
| **C16** | NT, SW | No | >1/week | None | None | None |
| **C17** | NT, CA, SW | No | Everyday | L-OH-Tryptophane 50 mg (*) | None | None |
| **C18** | SW | Yes | >1/month | None | Mild insomnia, flow-limitation | None |
| **C19** | NT, CA, SW | No | >1/week | Isotretinoinum 20 mg (*) | None | Acne |

**Table S2. Clinical and Demographic Characteristics of Children DoA Participants**

ID – Participant code; DoA: Disorders of Arousal, Subtype – CA = Confusional arousals, SW = Sleepwalking, ST = Sleep terrors, Family history – Presence of the disorder in family (Yes, No, Unknown); Frequency – Episode occurrence rate; Medications – Current treatments at the time of enrollment; Sleep comorbidities – Other diagnosed sleep disorders (OSA = obstructive sleep apnea); Other major comorbidities – Other physical health conditions or neuro-psychiatric comorbidities – (ADHD = attention deficit hyperactivity disorder). (*) Medication withheld on the day of recording; discontinued three days prior. Note: C11 was excluded after the recording due to severe OSA for pediatric age and C8 due to the detection of isolated frontal sharp waves, more prominent on the right side, visible during wake and all sleep stages, without clinical correlates. C12 and C18 exhibited flow-limitation but had an AHI <3 and were therefore retained for analysis.

| **ID** | **LMSI (n/h)** | **PLMSI (n/h)** | **AHI (n/h)** | **TIB (min)** | **TST (min)** | **SL (min)** | **REML (min)** | **WASO (min)** | **SE (%)** | **N1 (min)** | **N1 (%)** | **N2 (min)** | **N2 (%)** | **N3 (min)** | **N3 (%)** | **REM (min)** | **REM (%)** | **AI (n/h)** | **NREM-AI (n/h)** | **REM-AI (n/h)** |
| --- | --- | --- | --- | --- | --- | --- | --- | --- | --- | --- | --- | --- | --- | --- | --- | --- | --- | --- | --- | --- |
| **A1** | 6.0 | 0.0 | 3.6 | 242.1 | 224.1 | 4.0 | 99.0 | 14.0 | 92.5 | 15.1 | 6.7 | 130.5 | 58.2 | 58.0 | 25.9 | 20.5 | 9.2 | 8.8 | 14.7 | 11.7 |
| **A2** | 18.4 | 4.8 | 7.5 | 464.0 | 411.6 | 9.5 | 180.5 | 42.9 | 88.7 | 47.5 | 11.5 | 255.6 | 62.1 | 28.9 | 7.0 | 79.6 | 19.3 | 16.5 | 29.6 | 12.1 |
| **A3** | 24.2 | 3.7 | 8.9 | 486.5 | 382.0 | 14.0 | 97.5 | 26.5 | 78.5 | 27.5 | 7.2 | 162.0 | 42.4 | 73.5 | 19.2 | 119.0 | 31.1 | 15.6 | 31.9 | 8.6 |
| **A4** | 31.9 | 21.0 | 5.1 | 405.0 | 373.3 | 8.5 | 144.5 | 23.2 | 92.2 | 47.2 | 12.6 | 171.0 | 45.8 | 64.7 | 17.3 | 90.5 | 24.2 | 15.8 | 29.3 | 8.6 |
| **A5** | 27.8 | 11.4 | 0.1 | 443.0 | 387.7 | 6.0 | 143.5 | 49.4 | 87.5 | 43.8 | 11.3 | 200.5 | 51.7 | 59.5 | 15.3 | 83.8 | 21.6 | 22.3 | 33.6 | 23.6 |
| **A6** | 7.2 | 0.6 | 0.5 | 436.3 | 377.8 | 8.8 | 126.2 | 49.6 | 86.6 | 29.5 | 7.8 | 201.2 | 53.2 | 82.2 | 21.8 | 65.0 | 17.2 | 14.3 | 26.1 | 11.1 |
| **A7** | 9.8 | 0.7 | 23.5 | 417.6 | 332.4 | 3.5 | 93.0 | 81.7 | 79.6 | 38.1 | 11.5 | 125.9 | 37.9 | 74.0 | 22.3 | 94.4 | 28.4 | 18.9 | 36.3 | 5.7 |
| **A8** | 14.4 | 2.3 | 1.2 | 436.0 | 363.0 | 5.0 | 137.5 | 73.0 | 83.3 | 18.0 | 5.0 | 145.5 | 40.1 | 115.5 | 31.8 | 84.0 | 23.1 | 18.8 | 43.4 | 5.7 |
| **A9** | 10.7 | 2.0 | 2.2 | 443.4 | 403.4 | 9.0 | 85.5 | 28.5 | 91.0 | 15.4 | 3.8 | 138.0 | 34.2 | 157.5 | 39.0 | 92.5 | 22.9 | 15.5 | 26.2 | 13.6 |
| **A10** | 16.8 | 6.4 | 1.3 | 401.4 | 358.4 | 10.0 | 196.0 | 33.0 | 89.3 | 35.1 | 9.8 | 165.4 | 46.2 | 72.9 | 20.3 | 84.9 | 23.7 | 19.6 | 36.0 | 13.4 |
| **A11** | 20.5 | 12.5 | 0.0 | 408.0 | 361.6 | 2.0 | 87.0 | 44.4 | 88.6 | 19.9 | 5.5 | 159.5 | 44.1 | 79.6 | 22.0 | 102.6 | 28.4 | 11.3 | 12.1 | 13.4 |
| **A12** | 17.0 | 2.5 | 0.0 | 397.4 | 228.2 | 19.5 | 232.5 | 149.8 | 57.4 | 46.0 | 20.2 | 79.7 | 34.9 | 66.0 | 28.9 | 36.5 | 16.0 | 23.9 | 40.1 | 8.2 |
| **A13** | 7.2 | 1.2 | 2.6 | 388.0 | 342.5 | 8.0 | 106.0 | 37.5 | 88.3 | 25.0 | 7.3 | 135.5 | 39.6 | 90.5 | 26.4 | 91.5 | 26.7 | 13.5 | 30.6 | 5.9 |
| **A14** | 11.9 | 4.4 | 0.0 | 371.0 | 268.8 | 3.5 | 114.5 | 98.7 | 72.5 | 17.7 | 6.6 | 151.1 | 56.2 | 52.9 | 19.7 | 47.1 | 17.5 | 17.9 | 29.8 | 19.1 |
| **A15** | 15.5 | 1.4 | 0.0 | 404.6 | 324.1 | 9.5 | 152.5 | 71.0 | 80.1 | 51.1 | 15.8 | 178.0 | 54.9 | 21.0 | 6.5 | 74.0 | 22.8 | 23.9 | 44.1 | 16.2 |
| **A16** | 15.3 | 4.7 | 3.3 | 374.7 | 340.6 | 12.5 | 162.0 | 21.6 | 90.9 | 13.3 | 3.9 | 165.9 | 48.7 | 87.8 | 25.8 | 73.5 | 21.6 | 16.6 | 23.4 | 25.3 |
| **A17** | 10.4 | 2.4 | 6.9 | 458.2 | 417.8 | 9.5 | 47.0 | 30.8 | 91.2 | 41.0 | 9.8 | 191.7 | 45.9 | 72.0 | 17.2 | 113.2 | 27.1 | 23.1 | 50.0 | 11.1 |
| **A18** | 31.4 | 16.8 | 24.2 | 470.2 | 379.4 | 19.5 | 102.5 | 71.3 | 80.7 | 30.5 | 8.0 | 186.7 | 49.2 | 81.8 | 21.6 | 80.4 | 21.2 | 28.5 | 63.8 | 3.7 |
| **A19** | 6.0 | 0.0 | 0.5 | 389.4 | 334.9 | 29.0 | 98.5 | 25.5 | 86.0 | 23.7 | 7.1 | 190.7 | 56.9 | 67.3 | 20.1 | 53.2 | 15.9 | 13.6 | 25.1 | 4.5 |
| **A20** | 10.3 | 0.7 | 9.0 | 462.8 | 377.9 | 25.5 | 94.0 | 59.4 | 81.7 | 25.7 | 6.8 | 178.9 | 47.3 | 79.0 | 20.9 | 94.3 | 25.0 | 16.4 | 25.4 | 18.4 |
| **A21** | 18.7 | 2.5 | 0.2 | 498.0 | 338.0 | 20.5(*) | 77.0 | 125.0 | 67.9 | 43.3 | 12.8 | 178.7 | 52.9 | 64.5 | 19.1 | 51.5 | 15.2 | 21.8 | 41.0 | 4.7 |
| **A22** | 5.1 | 0.0 | 0.0 | 369.7 | 215.2 | 16.0 | 386.5 | 138.5 | 58.2 | 54.0 | 25.1 | 98.7 | 45.9 | 14.0 | 6.5 | 48.5 | 22.5 | 56.3 | 129.5 | 5.0 |

**Table S3. Sleep Study Summary Table of Adult DoA Participants**

ID = Subject identifier; LMSI (n/h) = Limb Movement Sleep Index (number/hour); PLMSI (n/h) = Periodic Limb Movement Sleep Index (number/hour); AHI (n/h) = Apnea–Hypopnea Index (number/hour); TIB (min) = Time in Bed; TST (min) = Total Sleep Time; SL (min) = Sleep Latency; REML (min) = REM Latency; WASO (min) = Wake After Sleep Onset; SE (%) = Sleep Efficiency; N1, N2, N3, REM (min) = minutes spent in each sleep stage; N1, N2, N3, REM (%) = percentage of TST in each sleep stage; AI (n/h) = Arousal Index; NREM AI (n/h) = NREM Arousal Index; REM AI (n/h) = REM Arousal Index.

(*) – Sleep latency was underestimated due to technical issues occurring between light-off and sleep onset.

| **ID** | **LMSI (n/h)** | **PLMSI (n/h)** | **AHI (n/h)** | **TIB (min)** | **TST (min)** | **SL (min)** | **REML (min)** | **WASO (min)** | **SE (%)** | **N1 (min)** | **N1 (%)** | **N2 (min)** | **N2 (%)** | **N3 (min)** | **N3 (%)** | **REM (min)** | **REM (%)** | **AI (n/h)** | **NREM AI (n/h)** | **REM AI (n/h)** |
| --- | --- | --- | --- | --- | --- | --- | --- | --- | --- | --- | --- | --- | --- | --- | --- | --- | --- | --- | --- | --- |
| **C1** | 9.8 | 4.0 | 1.9 | 418.4 | 355.5 | 4.5 | 142.0 | 58.4 | 85.0 | 32.0 | 9.0 | 117.3 | 33.0 | 147.4 | 41.5 | 58.8 | 16.5 | 16.2 | 29.5 | 11.2 |
| **C2** | 11.8 | 0.6 | 0.3 | 448.6 | 421.0 | 8.0 | 141.0 | 19.6 | 93.9 | 20.0 | 4.8 | 160.5 | 38.1 | 149.5 | 35.5 | 91.0 | 21.6 | 10.1 | 12.7 | 16.5 |
| **C3** | 16.8 | 4.5 | 0.3 | 483.4 | 391.4 | 62.0 | 227.5 | 30.0 | 81.0 | 20.6 | 5.3 | 170.6 | 43.6 | 135.4 | 34.6 | 64.9 | 16.6 | 11.3 | 22.8 | 4.6 |
| **C4** | 15.8 | 3.2 | 1.6 | 445.4 | 417.8 | 6.7 | 121.8 | 20.9 | 93.8 | 32.5 | 7.8 | 148.5 | 35.5 | 151.8 | 36.3 | 85.0 | 20.3 | 14.2 | 26.3 | 9.2 |
| **C5** | 15.0 | 6.0 | 0.2 | 491.2 | 348.0 | 10.0 | 244.0 | 142.2 | 70.8 | 22.6 | 6.5 | 152.4 | 43.8 | 104.0 | 29.9 | 69.0 | 19.8 | 12.8 | 24.5 | 2.6 |
| **C6** | 14.4 | 3.3 | 1.3 | 543.2 | 493.9 | 18.5 | 103.5 | 30.8 | 90.9 | 39.0 | 7.9 | 169.2 | 34.3 | 166.0 | 33.6 | 119.7 | 24.2 | 10.6 | 17.6 | 12.0 |
| **C7** | 14.7 | 2.1 | 0.6 | 507.0 | 419.0 | 41.5 | 78.0 | 46.5 | 82.6 | 29.5 | 7.0 | 141.0 | 33.7 | 132.0 | 31.5 | 116.5 | 27.8 | 14.6 | 19.8 | 17.0 |
| **C8** | 22.4 | 9.8 | 2.1 | 517.4 | 458.9 | 30.5 | 169.5 | 28.0 | 88.7 | 25.2 | 5.5 | 172.3 | 37.5 | 139.2 | 30.3 | 122.3 | 26.6 | 12.8 | 25.0 | 7.9 |
| **C9** | 17.8 | 10.0 | 0.6 | 428.0 | 379.3 | 7.7 | 85.5 | 41.0 | 88.6 | 37.6 | 9.9 | 157.5 | 41.5 | 115.2 | 30.4 | 69.0 | 18.2 | 11.1 | 13.1 | 12.2 |
| **C10** | 22.8 | 4.8 | 0.2 | 434.5 | 373.0 | (*) | 137.5 | 61.5 | 85.8 | 26.0 | 7.0 | 124.0 | 33.2 | 143.5 | 38.5 | 79.5 | 21.3 | 11.7 | 18.0 | 13.6 |
| **C11** | 25.9 | 4.9 | 13.8 | 461.1 | 388.6 | 11.5 | 135.0 | 61.0 | 84.3 | 39.1 | 10.1 | 146.6 | 37.7 | 115.0 | 29.6 | 88.0 | 22.6 | 13.1 | 23.9 | 7.5 |
| **C12** | 35.9 | 19.4 | 1.9 | 465.9 | 403.5 | 9.5 | 152.0 | 52.9 | 86.6 | 35.7 | 8.8 | 121.0 | 30.0 | 141.3 | 35.0 | 105.5 | 26.1 | 19.3 | 34.2 | 25.6 |
| **C13** | 11.2 | 2.6 | 2.5 | 499.6 | 438.0 | 39.5 | 72.5 | 22.1 | 87.7 | 39.4 | 9.0 | 163.3 | 37.3 | 129.5 | 29.6 | 105.9 | 24.2 | 11.5 | 24.9 | 7.4 |
| **C14** | 8.4 | 0.0 | 0.4 | 476.4 | 404.4 | 57.0 | 153.0 | 15.0 | 84.9 | 13.9 | 3.4 | 195.0 | 48.2 | 98.5 | 24.4 | 97.0 | 24.0 | 14.2 | 21.1 | 17.9 |
| **C15** | 9.0 | 0.9 | 0.6 | 474.3 | 420.7 | 37.0 | 196.0 | 16.5 | 88.7 | 12.2 | 2.9 | 185.9 | 44.2 | 150.8 | 35.9 | 71.8 | 17.1 | 9.6 | 13.4 | 16.7 |
| **C16** | 7.0 | 0.0 | 2.2 | 481.7 | 437.0 | 3.5 | 123.5 | 41.2 | 90.7 | 37.7 | 8.6 | 154.0 | 35.2 | 120.3 | 27.5 | 125.0 | 28.6 | 14.8 | 29.2 | 12.5 |
| **C17** | 12.1 | 4.9 | 0.8 | 487.9 | 429.5 | 15.0 | 44.5 | 21.9 | 88.0 | 18.0 | 4.2 | 193.6 | 45.1 | 118.5 | 27.6 | 99.4 | 23.1 | 10.6 | 16.7 | 9.1 |
| **C18** | 5.8 | 0.0 | 0.0 | 412.9 | 397.0 | 4.5 | 229.0 | 11.4 | 96.1 | 7.6 | 1.9 | 195.0 | 49.1 | 116.6 | 29.4 | 77.8 | 19.6 | 6.3 | 9.4 | 8.5 |
| **C19** | 1.7 | 0.0 | 0.1 | 478.3 | 431.8 | 4.0 | 75.5 | 37.5 | 90.3 | 22.5 | 5.2 | 182.3 | 42.2 | 118.5 | 27.4 | 108.5 | 25.1 | 10.8 | 23.4 | 6.1 |

**Table S4. Sleep Study Summary Table of Children DoA Participants**

ID = Subject identifier; LMSI (n/h) = Limb Movement Sleep Index (number/hour); PLMSI (n/h) = Periodic Limb Movement Sleep Index (number/hour); AHI (n/h) = Apnea–Hypopnea Index (number/hour); TIB (min) = Time in Bed; TST (min) = Total Sleep Time; SL (min) = Sleep Latency; REML (min) = REM Latency; WASO (min) = Wake After Sleep Onset; SE (%) = Sleep Efficiency; N1, N2, N3, REM (min) = minutes spent in each sleep stage; N1, N2, N3, REM (%) = percentage of TST in each sleep stage; AI (n/h) = Arousal Index; NREM AI (n/h) = NREM Arousal Index; REM AI (n/h) = REM Arousal Index.

(*) – Recording commenced after the participant had already fallen asleep, owing to technical issues.

|  |  | **DOA episodes** | **Physiological Motor arousals** |
| --- | --- | --- | --- |
| **Adult group** | | | |
|  | Subjects with episodes (n) | 12 (60%) | 18 (90%) |
|  | Number of episodes (n) | 34 | 68 |
|  | Episodes per subject (mean ± SD, n) | 1.7 ± 2.0 | 3.4 ± 2.5 |
|  | Duration (mean ± SD, s) | 16.0 ± 8.4 | 12.6 ± 10.1 |
|  | Latency from onset (mean ± SD, min) | 115.7 ± 96.8 | 143.3 ± 105.3 |
| **Children group** | | | |
|  | Subjects with episodes (n) | 15 (82%) | 16 (94%) |
|  | Number of episodes (n) | 50 | 78 |
|  | Episodes per subject (mean ± SD, n) | 2.9 ± 2.5 | 4.6 ± 2.9 |
|  | Duration (mean ± SD, s) | 31.0 ± 16.2 | 14.8 ± 9.5 |
|  | Latency from onset (mean ± SD, min) | 144.1 ± 87.1 | 136.2 ± 93.1 |

**Table S5. DoA Episodes and Physiological Motor Arousals in Adult and Children Participants**

Comparison between DoA episodes and physiological motor arousals in adults and children. Values are reported as counts (n), percentages (%), or means ± standard deviations (SD). “Subjects with episodes” refers to participants presenting at least one recorded episode. “Number of episodes” is the total count observed. “Episodes per subject” represents the mean number of episodes per participant. “Duration” is the mean length of episodes in seconds. “Latency from onset” indicates the mean time from sleep onset to the first recorded episode, expressed in minutes.

| Delta |  | Cluster ID | Cluster Size Scout | Cluster Size Vertices | Max t | Max Scout | Mean t |
| --- | --- | --- | --- | --- | --- | --- | --- |
|  | -5s - 0s | 1 | 142 | 11108 | 8.02 | Transverse Frontopolar Gyrus and Sulcus, Left | 3.07 |
|  | 0s - 5s | 1 | 2 | 8 | 4.82 | Middle Frontal Gyrus, Left | 4.22 |
|  |  | 2 | 54 | 1971 | 10.86 | Transverse Frontopolar Gyrus and Sulcus, Left | 4.87 |
|  | 5s - 10s | 1 | 13 | 169 | -2.32 | Middle Temporal Gyrus, Left | -3.64 |
|  |  | 2 | 13 | 379 | 8.42 | Transverse Frontopolar Gyrus and Sulcus, Left | 4.95 |
|  |  | 3 | 6 | 20 | -3.02 | Medial Occipito-Temporal (Lingual) Gyrus, Right | -3.92 |
|  |  | 4 | 5 | 12 | -3.07 | Posterior Lateral Fissure, Right | -3.39 |
|  |  | 5 | 4 | 12 | 5.96 | Inferior Frontal Gyrus (Triangular Part), Right | 5.30 |
|  |  | 6 | 15 | 337 | 9.27 | Transverse Frontopolar Gyrus and Sulcus, Right | 4.90 |
|  |  | 7 | 2 | 5 | 6.17 | Transverse Frontopolar Gyrus and Sulcus, Right | 5.32 |
|  |  | 8 | 101 | 6798 | -2.13 | Lateral Occipito-Temporal (Fusiform) Gyrus, Left | -4.02 |
|  | 10s - 15s | 1 | 12 | 226 | 8.53 | Orbital Gyrus, Left | 5.09 |
|  |  | 2 | 117 | 9707 | -1.96 | Inferior Parietal Gyrus (Supramarginal), Left | -4.21 |
|  |  | 3 | 3 | 28 | 6.39 | Orbital Gyrus, Right | 5.29 |
|  |  | 4 | 2 | 18 | 5.61 | Frontomarginal Gyrus and Sulcus, Right | 4.87 |
| Beta | -5s - 0s | 1 | 134 | 11285 | 8.68 | Inferior Frontal Sulcus, Left | 3.50 |
|  | 0s - 5s | 1 | 148 | 14986 | 43.94 | Superior Temporal Gyrus (Planum Temporale), Right | 25.69 |
|  | 5s - 10s | 1 | 148 | 14986 | 36.89 | Superior Temporal Gyrus (Planum Temporale), Right | 22.43 |
|  | 10s - 15s | 1 | 148 | 14986 | 30.89 | Temporal Pole, Left | 20.33 |

**Table S6. DoA episodes vs baseline in adults**

Significant EEG clusters in Delta and Beta bands in the 20 seconds surrounding DoA episode onset (0s), compared to baseline slow wave activity recorded 2–3 minutes before the event. Results are organized in 5-second time bins. Legend: Time Windows: EEG data were analyzed in five consecutive 5-second bins: −5s to 0s, 0s to +5s, +5s to +10s, +10s to +15s relative to episode onset; Cluster Size (Scout): Number of anatomical scouts (brain regions) involved; Cluster Size (Vertices): Number of cortical vertices in the cluster.; Max Scout: Region with the peak statistical effect; Max/Mean t: Maximum and average t-statistics within each cluster.

| Delta |  | Cluster ID | Cluster Size Scout | Cluster Size Vertices | Max t | Max Scout | Mean t |
| --- | --- | --- | --- | --- | --- | --- | --- |
|  | -5s - 0s | 1 | 134 | 7642 | 6.79 | Superior Temporal Gyrus (Lateral Part), Left | 2.59 |
|  | 0s - 5s | 1 | 148 | 13903 | 17.82 | Superior Temporal Gyrus (Planum Polare), Left | 6.61 |
|  | 5s - 10s | 1 | 108 | 7374 | 15.30 | Rectus Gyrus, Left | 5.63 |
|  |  | 2 | 29 | 1069 | -2.91 | Central Sulcus, Right | -3.99 |
|  | 10s - 15s | 1 | 99 | 5711 | 15.52 | Rectus Gyrus, Left | 5.61 |
|  |  | 2 | 0 | 12 | 5.13 |  | 4.61 |
|  |  | 3 | 40 | 2227 | -2.64 | Intraparietal Sulcus and Transverse Parietal Sulcus, Right | -4.17 |
| Beta | -5s - 0s | 1 | 141 | 6574 | 4.46 | Subcentral Gyrus and Sulcus, Left | 2.28 |
|  | 0s - 5s | 1 | 148 | 14986 | 35.72 | Superior Temporal Gyrus (Lateral Part), Right | 19.16 |
|  | 5s - 10s | 1 | 148 | 14986 | 32.14 | Inferior Circular Sulcus of the Insula, Right | 18.59 |
|  | 10s - 15s | 1 | 148 | 14985 | 32.43 | Inferior Frontal Gyrus (Triangular Part), Left | 17.21 |

**Table S7. DoA episodes vs Baseline in children**

Significant EEG clusters in Delta and Beta bands in the 20 seconds surrounding DoA episode onset (0s), compared to baseline slow wave activity recorded 2–3 minutes before the event. Results are organized in 5-second time bins. Legend: Time Windows: EEG data were analyzed in five consecutive 5-second bins: −5s to 0s, 0s to +5s, +5s to +10s, +10s to +15s relative to episode onset; Cluster Size (Scout): Number of anatomical scouts (brain regions) involved; Cluster Size (Vertices): Number of cortical vertices in the cluster.; Max Scout: Region with the peak statistical effect; Max/Mean t: Maximum and average t-statistics within each cluster.

|  |  | Cluster ID | Cluster Size Scout | Cluster Size Vertices | Max t | Max Scout | Mean t |
| --- | --- | --- | --- | --- | --- | --- | --- |
| Delta | -5s - 0s | - | - | - | - | - | - |
|  | 0s - 5s | - | - | - | - | - | - |
|  | 5s - 10s | 1 | 41 | 1446 | 5.13 | Middle Frontal Gyrus, Left | 3.24 |
|  | 10s - 15s | 1 | 59 | 2402 | 5.25 | Orbital Gyrus, Left | 3.46 |
| Beta | -5s - 0s | - | - | - | - | - | - |
|  | 0s - 5s | - | - | - | - | - | - |
|  | 5s - 10s | 1 | 12 | 608 | 5.62 | - | 3.68 |
|  | 10s - 15s | 1 | 111 | 6340 | 5.59 | Lateral Occipito-Temporal Sulcus, Left | 3.09 |

**Table S8. DoA episodes vs typical motor arousal in adults**

Significant EEG clusters in Delta and Beta bands in the 20 seconds surrounding DoA episode onset (0s), compared to a similar EEG window surrounding physiological motor arousals. Results are organized in 5-second time bins. Legend: Time Windows: EEG data were analyzed in five consecutive 5-second bins: −5s to 0s, 0s to +5s, +5s to +10s, +10s to +15s relative to episode onset; Cluster Size (Scout): Number of anatomical scouts (brain regions) involved; Cluster Size (Vertices): Number of cortical vertices in the cluster; Max Scout: Region with the peak statistical effect; Max/Mean t: Maximum and average t-statistics within each cluster.

| Delta |  | Cluster ID | Cluster Size Scout | Cluster Size Vertices | Max t | Max Scout | Mean t |
| --- | --- | --- | --- | --- | --- | --- | --- |
|  | -5s - 0s | 1 | 1 | 3 | 2.87 | Subcallosal Gyrus, Left | 2.80 |
|  |  | 2 | 1 | 5 | 3.29 | Superior Frontal Sulcus, Left | 2.92 |
|  |  | 3 | 2 | 4 | 2.66 | Subcallosal Gyrus, Left | 2.52 |
|  |  | 4 | 4 | 71 | 3.26 | Superior Frontal Gyrus, Left | 2.30 |
|  |  | 5 | 3 | 7 | 2.67 | Inferior Temporal Sulcus, Right | 2.55 |
|  |  | 6 | 2 | 5 | 3.20 | Temporal Pole, Right | 2.59 |
|  |  | 7 | 148 | 11370 | 4.93 | Orbital Gyrus, Left | 2.72 |
|  |  | 8 | 2 | 3 | 3.50 |  | 3.22 |
|  |  | 9 | 3 | 13 | 3.55 | Pericallosal Sulcus, Right | 2.61 |
|  | 0s - 5s | 1 | 1 | 6 | 3.54 | Inferior Frontal Sulcus, Right | 3.08 |
|  |  | 2 | 3 | 14 | 3.24 | Anterior Vertical Ramus of Lateral Fissure, Right | 2.79 |
|  |  | 3 | 3 | 7 | 3.35 | Inferior Frontal Gyrus (Triangular Part), Right | 2.72 |
|  |  | 4 | 2 | 5 | 2.98 | Inferior Frontal Sulcus, Right | 2.72 |
|  |  | 5 | 103 | 4456 | 4.20 | Anterior Transverse Collateral Sulcus, Right | 2.49 |
|  | 5s - 10s | 1 | 94 | 5398 | 5.57 | Middle Frontal Sulcus, Right | 3.06 |
|  | 10 - 15s | 1 | 108 | 6916 | 6.63 | Middle Frontal Gyrus, Left | 3.52 |
| Beta | -5s - 0s | - | - | - | - |  | - |
|  | 0s - 5s | 1 | 75 | 3683 | 5.68 | Middle Frontal Gyrus, Right | 3.00 |
|  | 5s - 10s | 1 | 1 | 6 | 3.82 | Posterior Lateral Fissure, Left | 3.58 |
|  |  | 2 | 108 | 6873 | 5.32 | Middle Frontal Sulcus, Right | 3.08 |
|  | 10s - 15s | 1 | 126 | 8873 | 6.02 | Middle Frontal Sulcus, Left | 3.48 |

**Table S9. DoA episodes vs typical motor arousal in children**

Significant EEG clusters in Delta and Beta bands in the 20 seconds surrounding DoA episode onset (0s), compared to a similar EEG window surrounding physiological motor arousals. Results are organized in 5-second time bins. Legend: Time Windows: EEG data were analyzed in five consecutive 5-second bins: −5s to 0s, 0s to +5s, +5s to +10s, +10s to +15s relative to episode onset; Cluster Size (Scout): Number of anatomical scouts (brain regions) involved; Cluster Size (Vertices): Number of cortical vertices in the cluster; Max Scout: Region with the peak statistical effect; Max/Mean t: Maximum and average t-statistics within each cluster.

| **BRAIN LOBE** | **SPECIFIC BRAIN REGION** | **PREONSET** | | **POSTONSET** | | **AUTHOR/DATE** | **TECHNIQUE** |
| --- | --- | --- | --- | --- | --- | --- | --- |
| **FRONTAL** | **Dorsolateral Prefrontal Cortex (Superior and Middle frontal gyri)** |  | ↑ (low) delta |  | ↓ (low) delta, ↑ 4-30 Hz, no HSDA | Flamand et al., 2018 | sEEG |
|  | **Superior Frontal Gyrus** |  |  |  | Slow waves (left) | Terzaghi et al., 2009 | sEEG |
|  | **Middle Frontal Gyrus** |  |  |  | Slow waves (right) | Terzaghi et al., 2012 | sEEG |
|  | **Inferior Frontal Gyrus** |  |  |  | Slow waves (left) | Terzaghi et al., 2009 | sEEG |
|  |  |  |  |  | HSDA | Flamand et al., 2018 | sEEG |
|  | **Frontal associative cortex** |  |  |  | Regional cerebral blood flow < wake | Bassetti et al., 2000 | SPECT |
|  | **Orbitofrontal Cortex** |  |  |  | ↑ 4–10 Hz, HSDA | Flamand et al., 2018 | sEEG |
|  | **Gyrus Rectus** |  |  |  | No HSDA | Flamand et al., 2018 | sEEG |
|  | **Dorsolateral Premotor Cortex** |  | ↑ delta |  | ↓ (low) delta, ↑ 5-27 Hz,no HSDA | Flamand et al., 2018 | sEEG |
|  | **Supplementary Motor Area** |  | ↑ delta |  | ↓ (low) delta, no HSDA | Flamand et al., 2018 | sEEG |
|  |  |  |  |  | Fast activity (right) | Terzaghi et al., 2012 | sEEG |
|  | **Precentral Gyrus** |  |  |  | Fast activity ~25 Hz (left) | Terzaghi et al., 2009 | sEEG |
|  |  |  | ↑ (low) delta |  | ↓ (low) delta, ↑ 17-30 Hz, no HSDA | Flamand et al., 2018 | sEEG |
|  | **Precentral Operculum** |  | ↑ delta |  | No HSDA | Flamand et al., 2018 | sEEG |
|  | **Anterior Cingulate Cortex** |  | ↑ delta |  | ↓ (low) delta, HSDA, no high-frequency | Flamand et al., 2018 | sEEG |
|  |  |  |  |  | Fast activity (right) | Terzaghi et al., 2012 | sEEG |
|  | **Midcingulate Cortex** |  | ↑ delta (left) |  | Fast activity (~25 Hz) (left) | Terzaghi et al., 2009 | sEEG |
|  |  |  |  |  | ↓ (low) delta, no HSDA | Flamand et al., 2018 | sEEG |
| **PARIETAL** | **Posterior Cingulate Cortex** |  |  |  | ↓ (low) delta, occasional HSDA | Flamand et al., 2018 | sEEG |
|  |  |  |  |  | ↑ blood flow (compared to SWS) | Bassetti et al., 2000 | SPECT |
|  | **Superior Parietal Lobule** |  |  |  | Slow waves (left) | Terzaghi et al., 2009 | sEEG |
|  |  |  |  |  | ↓ (low) delta, ↑ 7-12 Hz, HSDA | Flamand et al., 2018 | sEEG |
|  | **Inferior Parietal Lobule** |  |  |  | Slow waves (left) | Terzaghi et al., 2009 | sEEG |
|  |  |  |  |  | ↓ (low) delta, HSDA | Flamand et al., 2018 | sEEG |
|  |  |  |  |  | ↑ delta | Sarasso et al., 2014 | sEEG |
|  | **Parietal associative cortex** |  |  |  | ↓ blood flow (compared to wake) | Bassetti et al., 2000 | SPECT |
|  | **Precuneus** |  |  |  | ↓ (low) delta, ↑ 4-7 Hz, HSDA | Flamand et al., 2018 | sEEG |
|  | **Postcentral Gyrus** |  |  |  | ↓ (low) delta, ↑ 13-30 Hz, occasional HSDA | Flamand et al., 2018 | sEEG |
|  | **Supramarginal Gyrus** |  |  |  | HDSA | Flamand et al., 2018 | sEEG |
|  | **Postcentral Operculum** |  | ↑ (low) delta |  | ↓ (low) delta, no HDSA | Flamand et al., 2018 | sEEG |
| **TEMPORAL** | **Amygdala** |  | No changes |  | Fast activity (right) | Terzaghi et al., 2012 | sEEG |
|  |  |  |  |  | No HDSA | Flamand et al., 2018 | sEEG |
|  | **Temporal Pole** |  | Slow wave bursts |  | Fast activity | Terzaghi et al., 2012 | sEEG |
|  | **Superior Temporal Gyrus** |  | ↑ delta |  | ↓ (low) delta, ↑ 13-30 Hz, rare HSDA | Flamand et al., 2018 | sEEG |
|  | **Middle Temporal Gyrus** |  | ↑ delta |  | ↓ (low) delta, ↑ 4-7 Hz, occasional HSDA | Flamand et al., 2018 | sEEG |
|  | **Inferior Temporal Gyrus** |  | ↑ delta |  | ↓ (low) delta, no HSDA | Flamand et al., 2018 | sEEG |
|  | **Hippocampus** |  | ↑ (low) delta |  | ↓ (low) delta, HSDA (50% cases), spindles (25% cases) | Flamand et al., 2018 | sEEG |
|  |  |  |  |  | Spindles (right) | Terzaghi et al., 2012 | sEEG |
|  | **Insula** |  | ↑ (low) delta |  | ↓ (low) delta, no HSDA | Flamand et al., 2018 | sEEG |
|  |  |  |  |  | Fast activity (right) | Terzaghi et al., 2012 | sEEG |
| **OCCIPITAL** | **Occipital Cortex** |  | No changes |  | No changes, no HSDA | Flamand et al., 2018 | sEEG |
| **THALAMUS** | **Ventral Intermediate Nucleus** |  |  |  | ↑ Beta, ↓Delta | Sarasso et al., 2014 | sEEG |
|  | **Pulvinar** |  |  |  | Occasional delayed HSDA | Flamand et al., 2018 | sEEG |
| **CEREBELLUM** | **Anterior Cerebellum** |  |  |  | ↑ blood flow (> 25% compared to SWS) | Bassetti et al., 2000 | SPECT |

**Table S10. Summary Of Stereo-EEG And Spect Findings By Brain Region**

Brain lobe – major brain lobe involved (e.g., frontal, temporal, parietal, occipital), specific brain region – anatomical sub-region within the lobe (e.g., dorsolateral prefrontal cortex, superior frontal gyrus), preonset – findings recorded before DoA motor onset, postonset – findings recorded after DoA onset, author/date – citation of the study reporting the findings, technique – method used to obtain the data (seeg = stereo-electroencephalography, spect = single-photon emission computed tomography). Blue = sleep-like activity; Red = wake-like activity; Violet = mixed activity


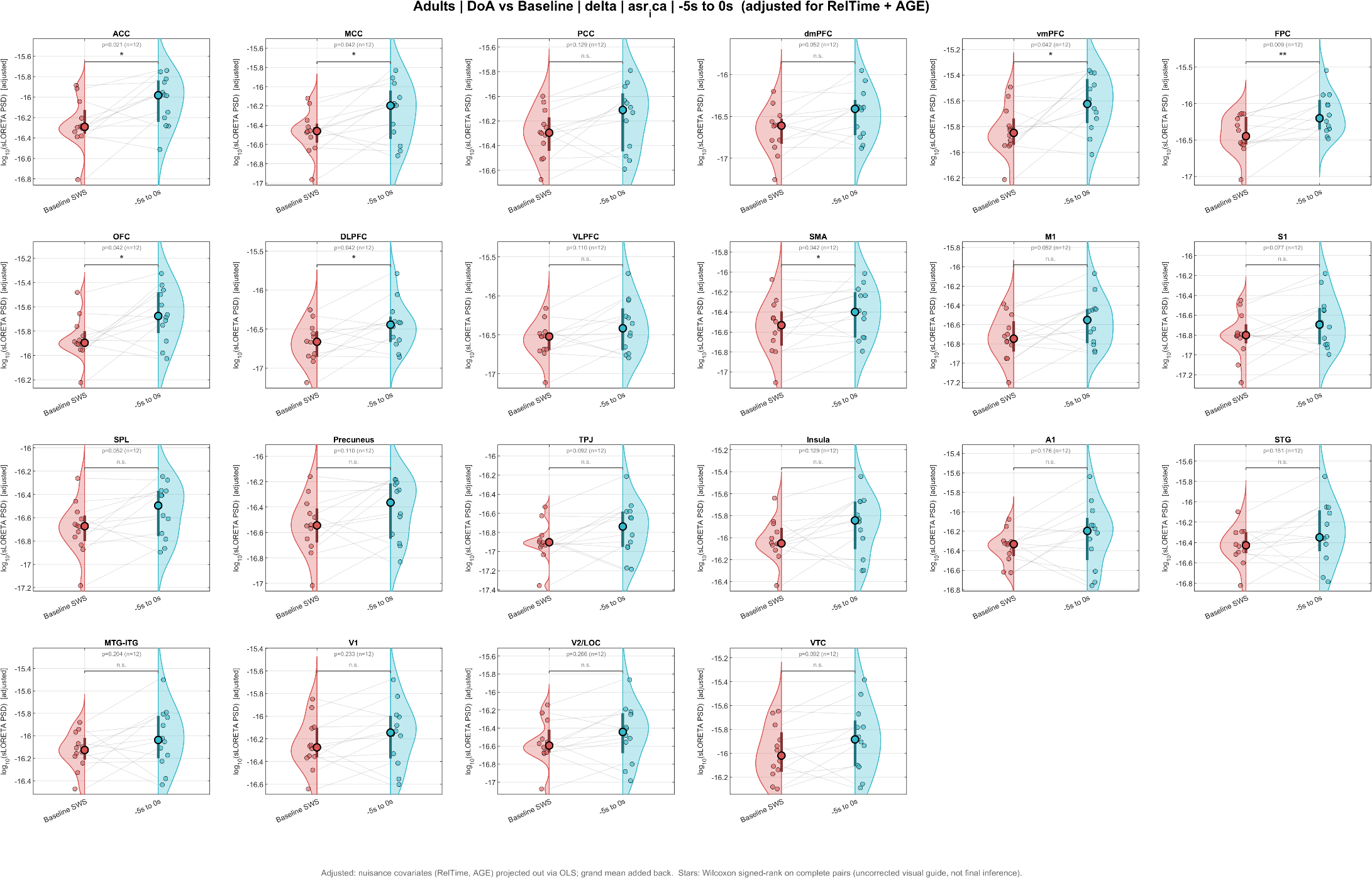


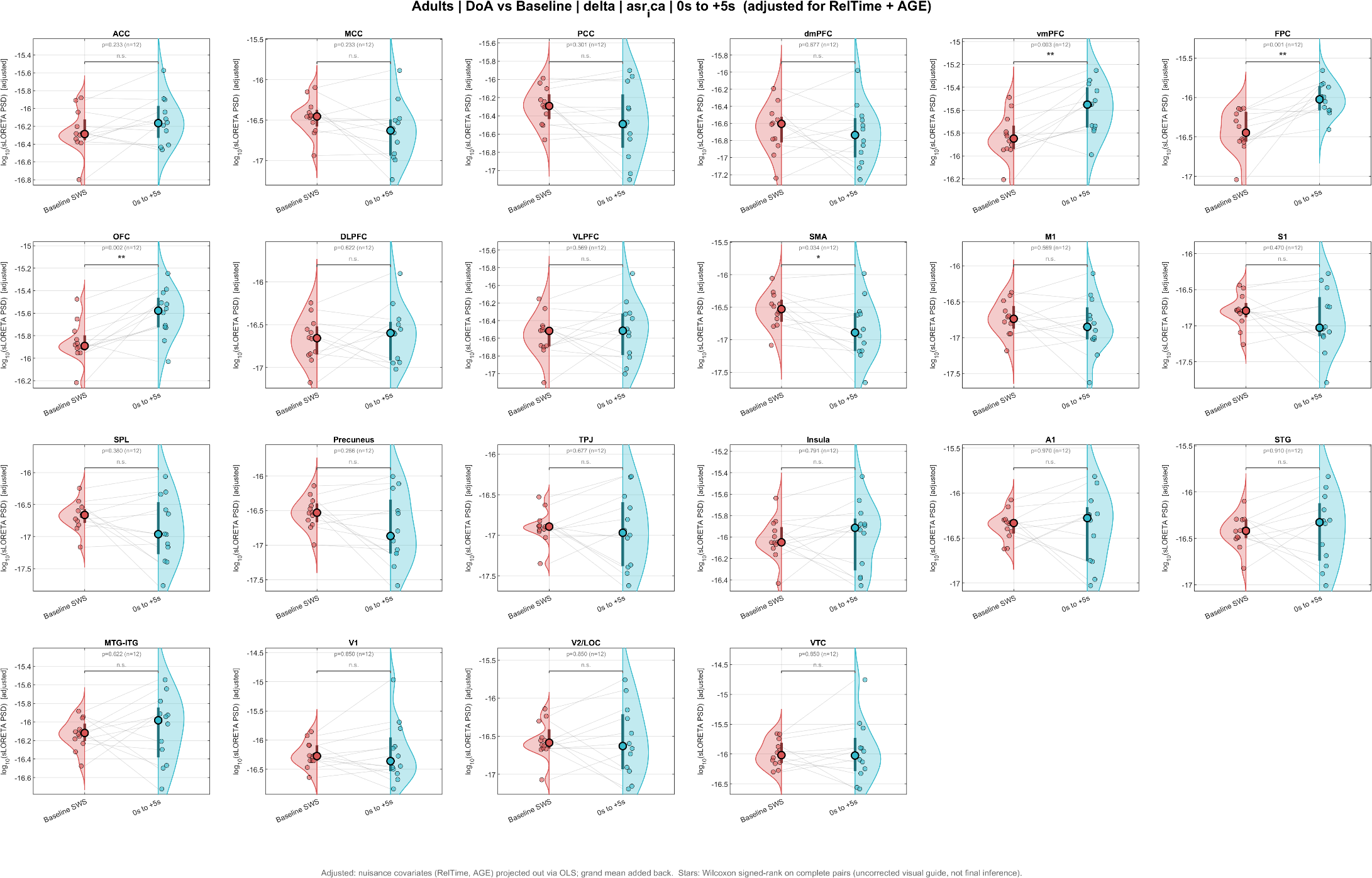


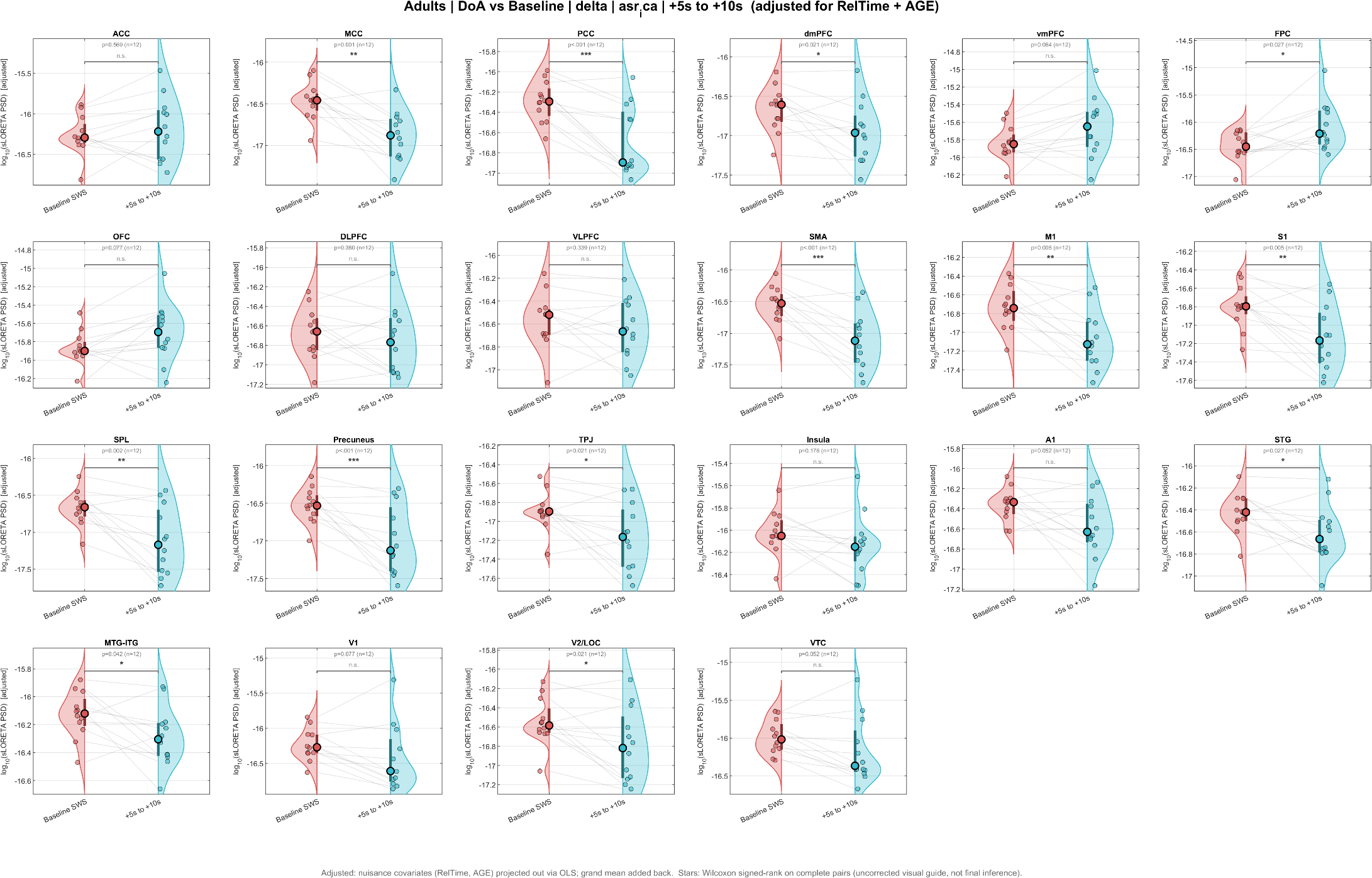


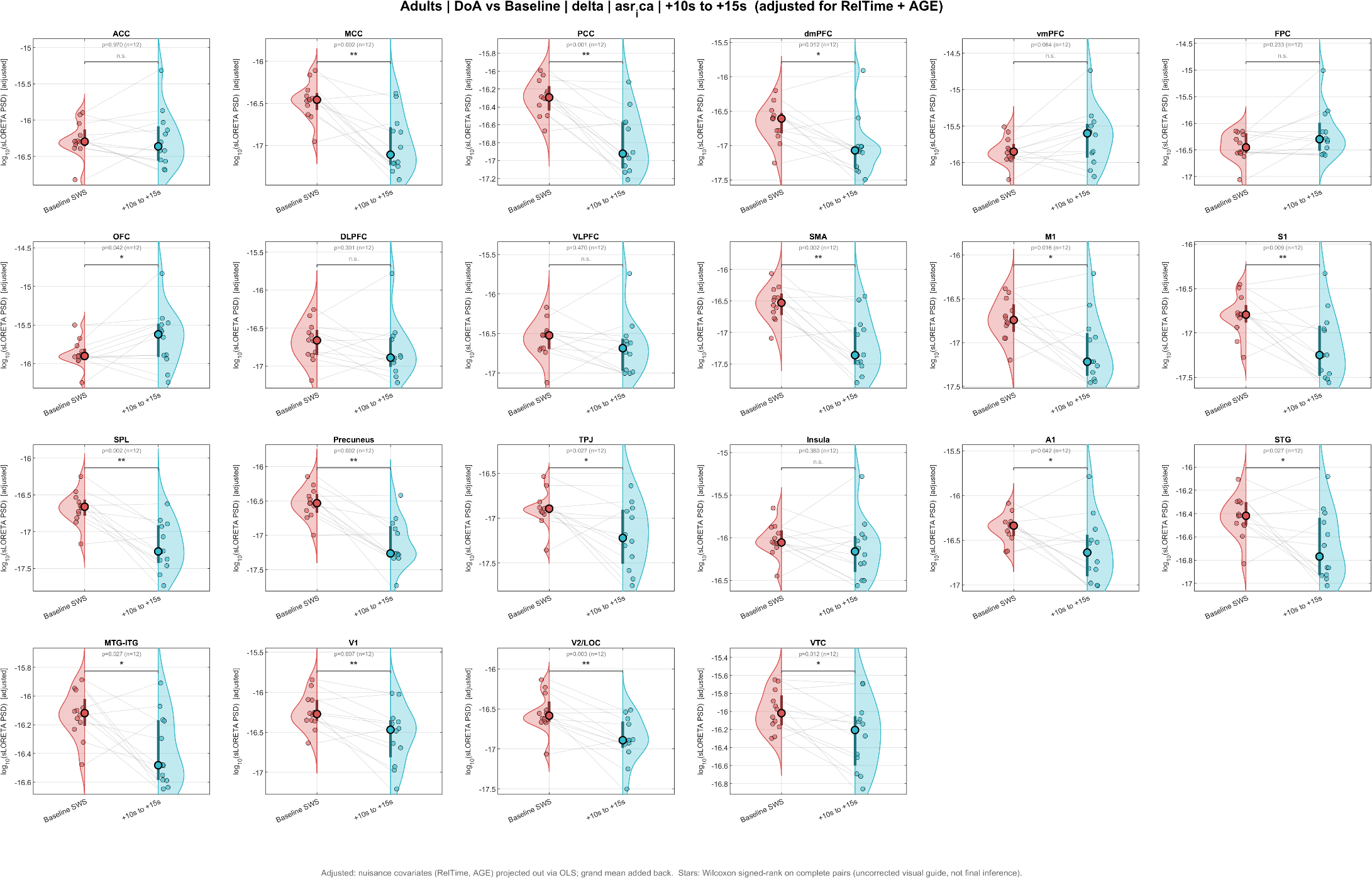


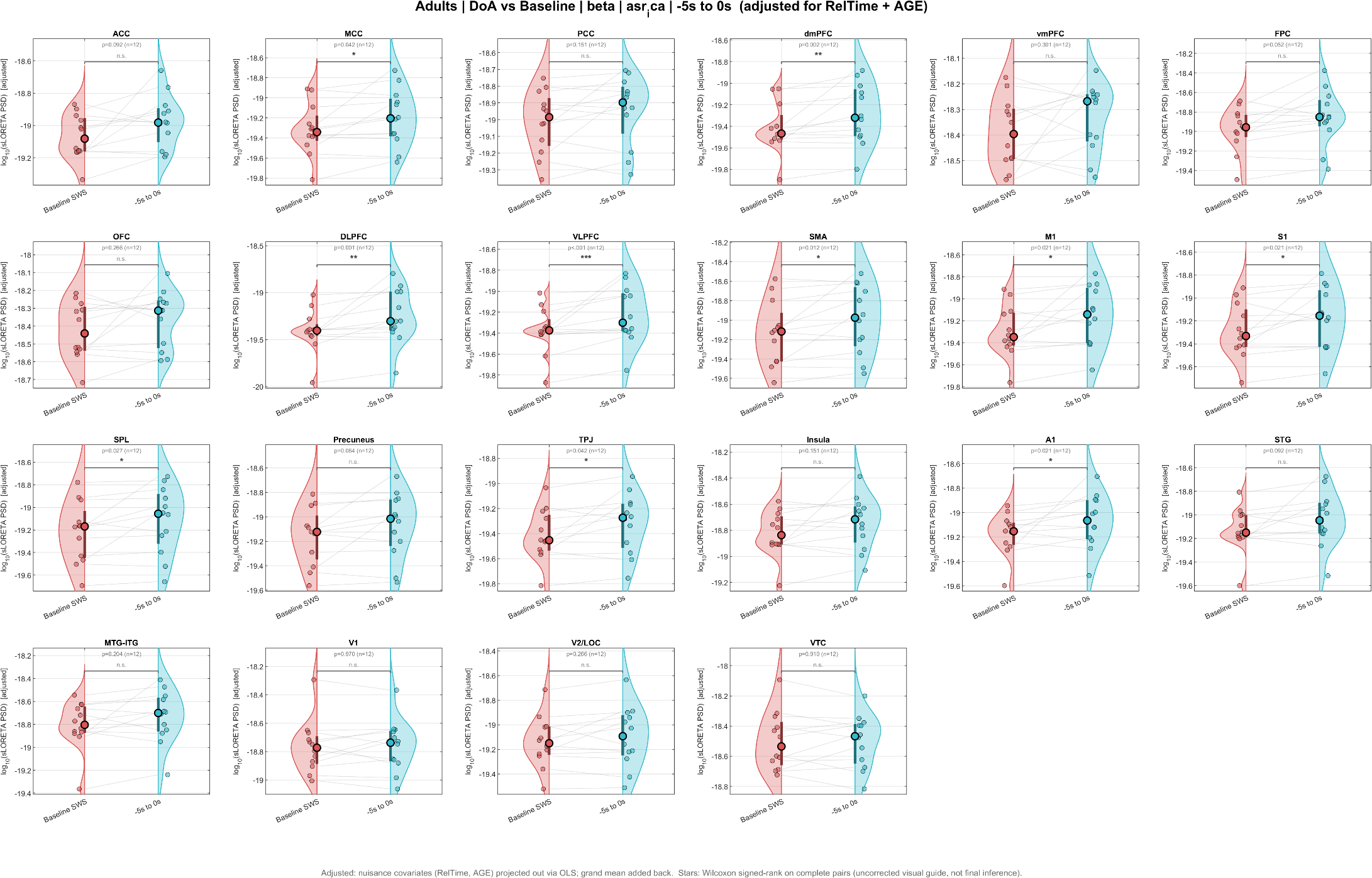


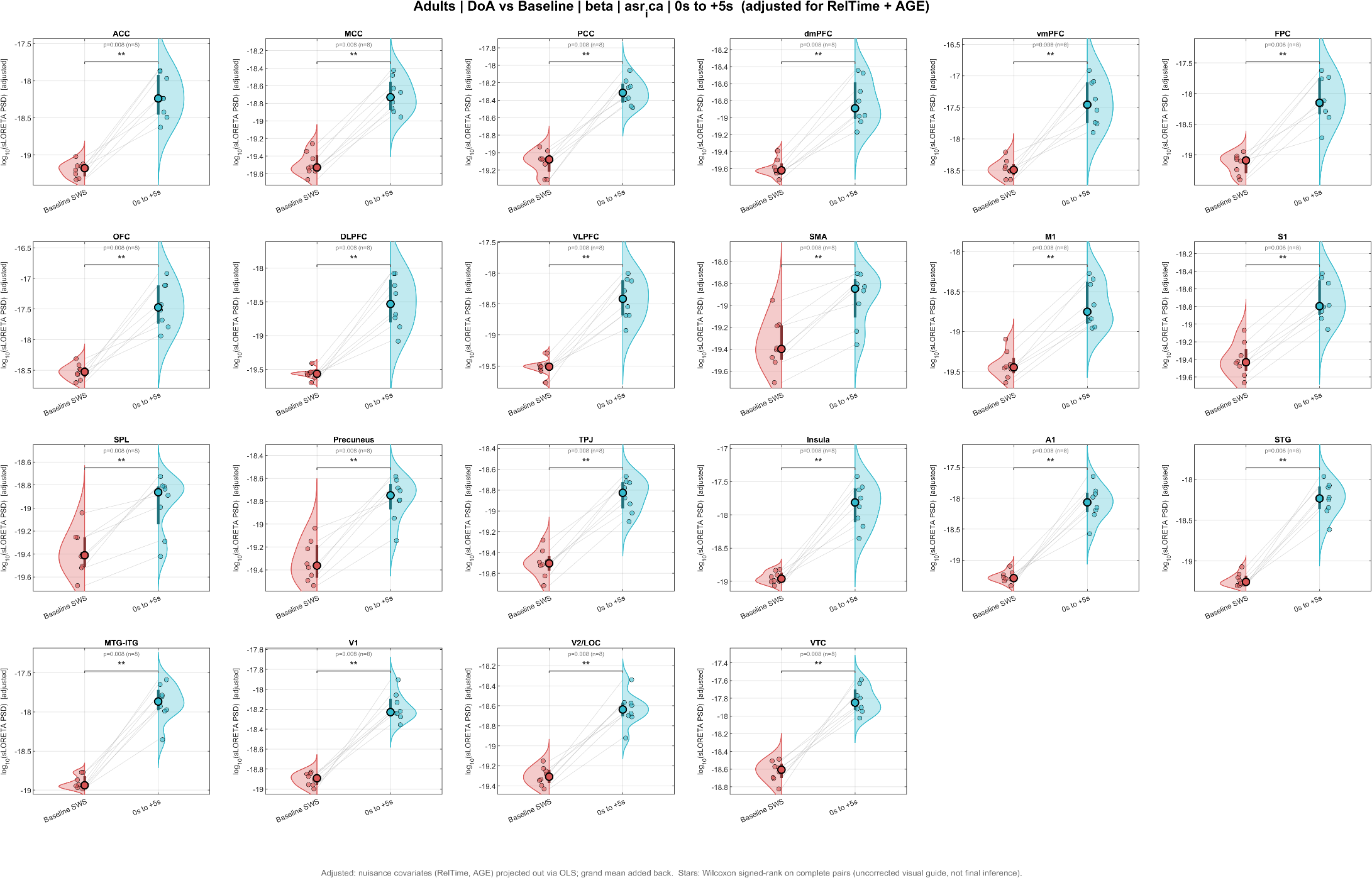


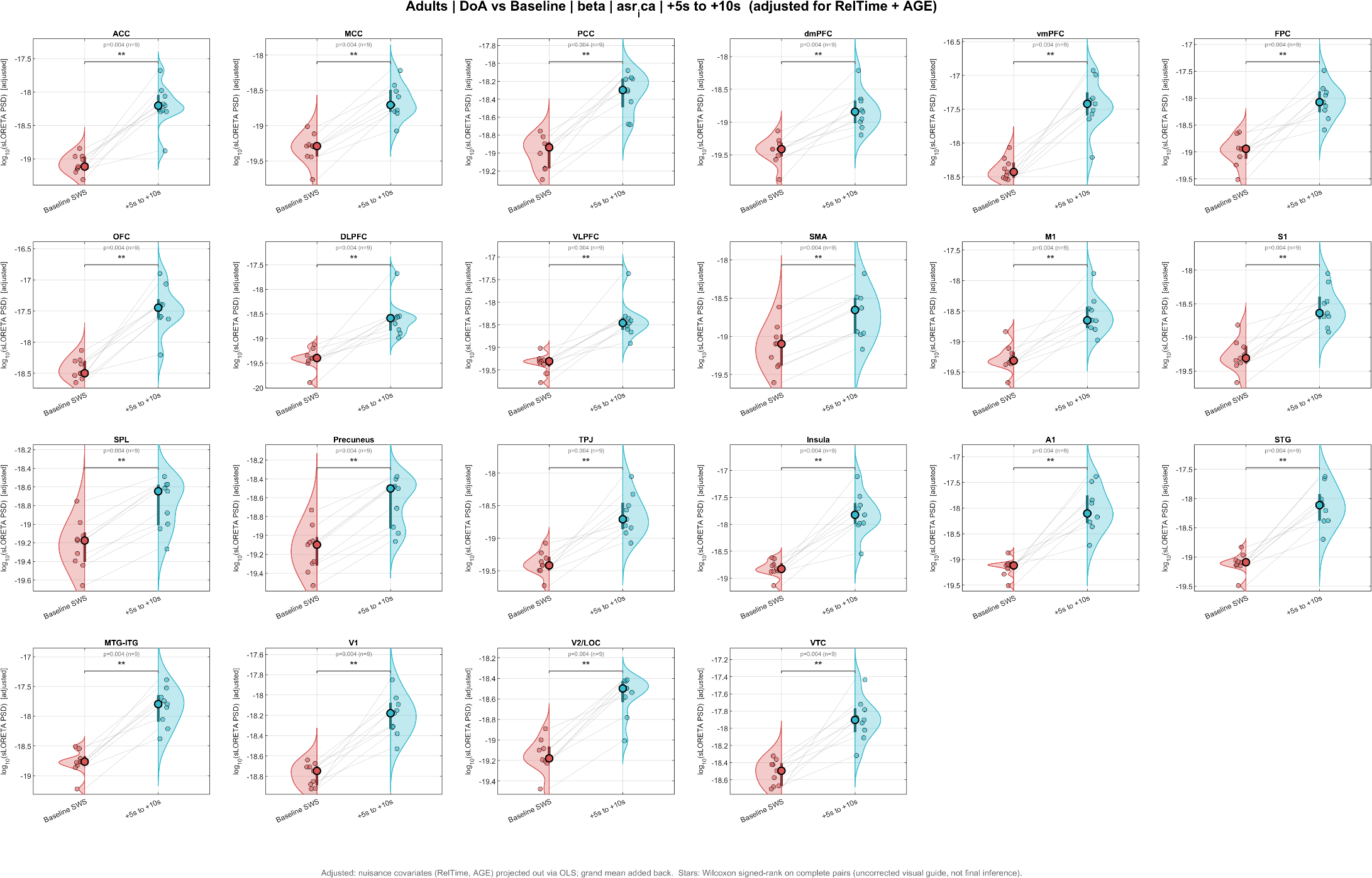


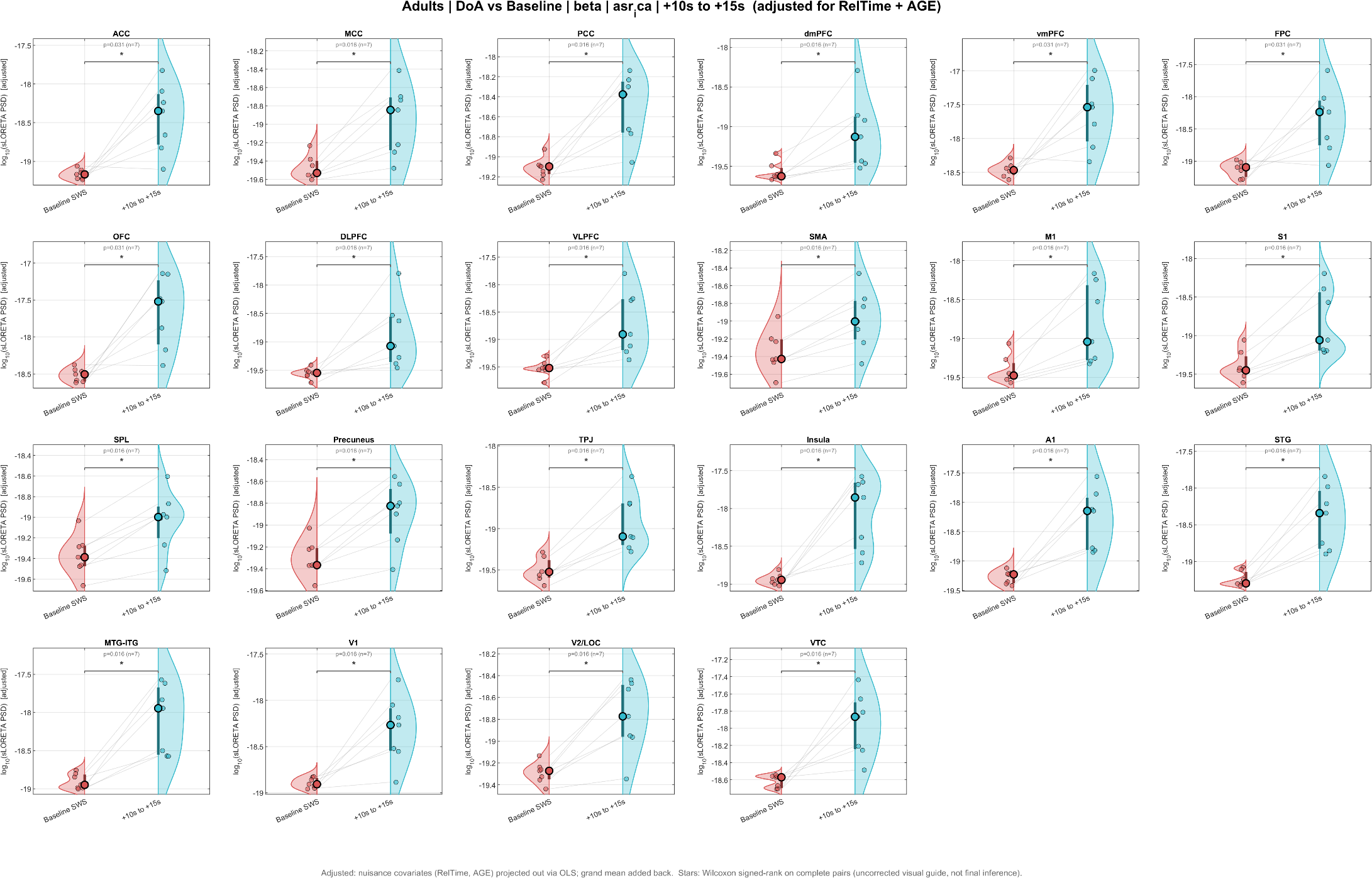


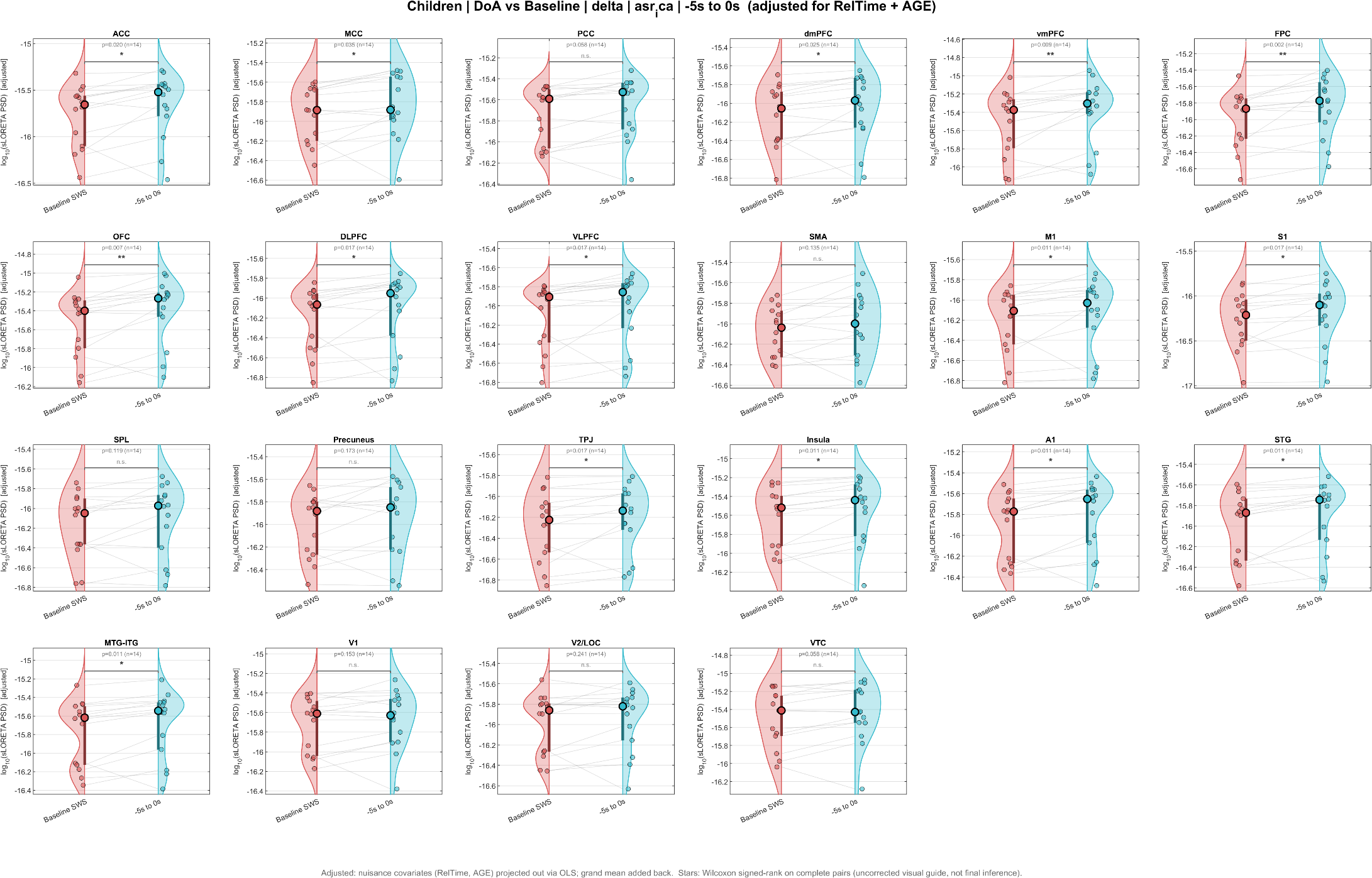


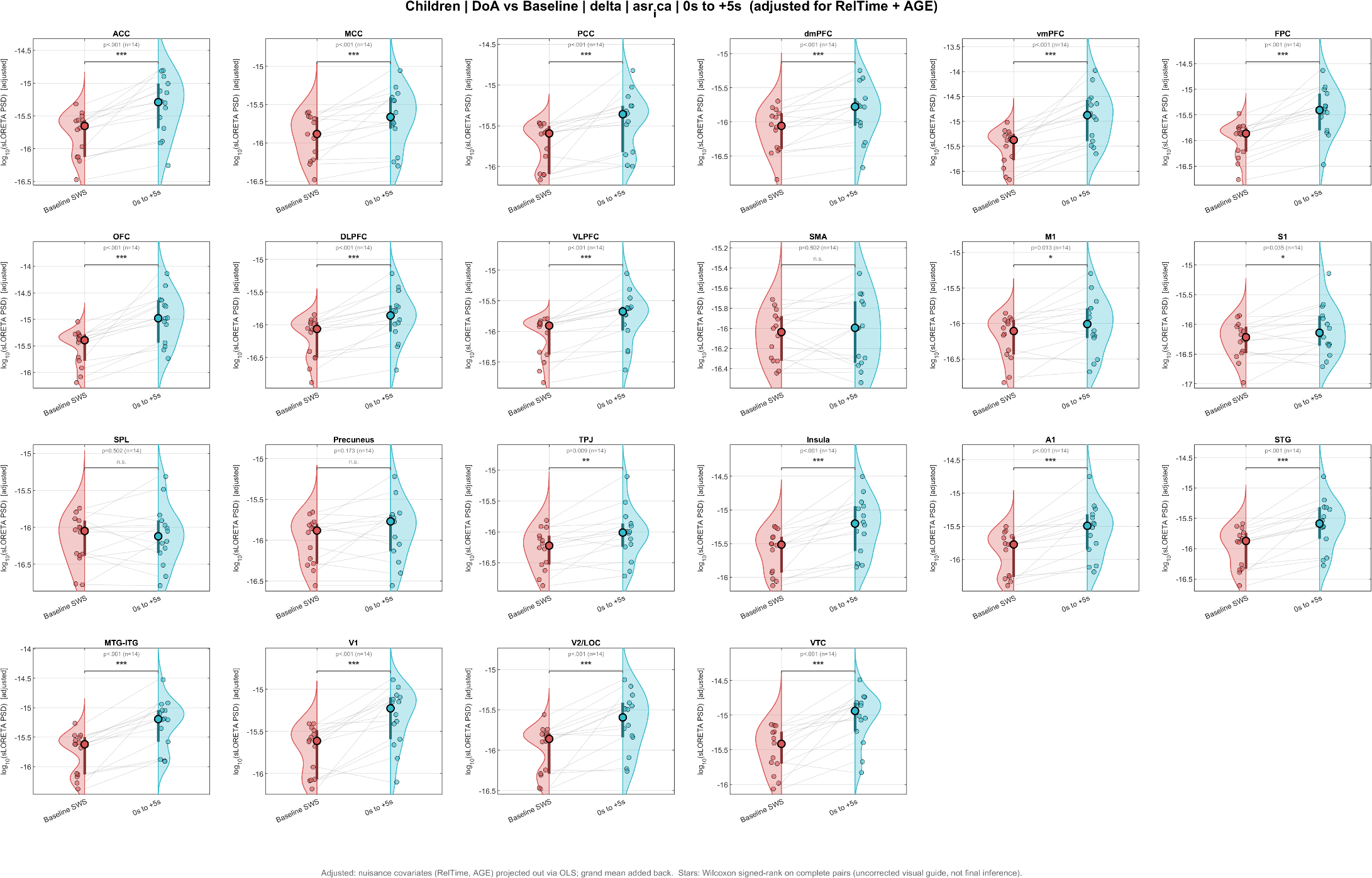


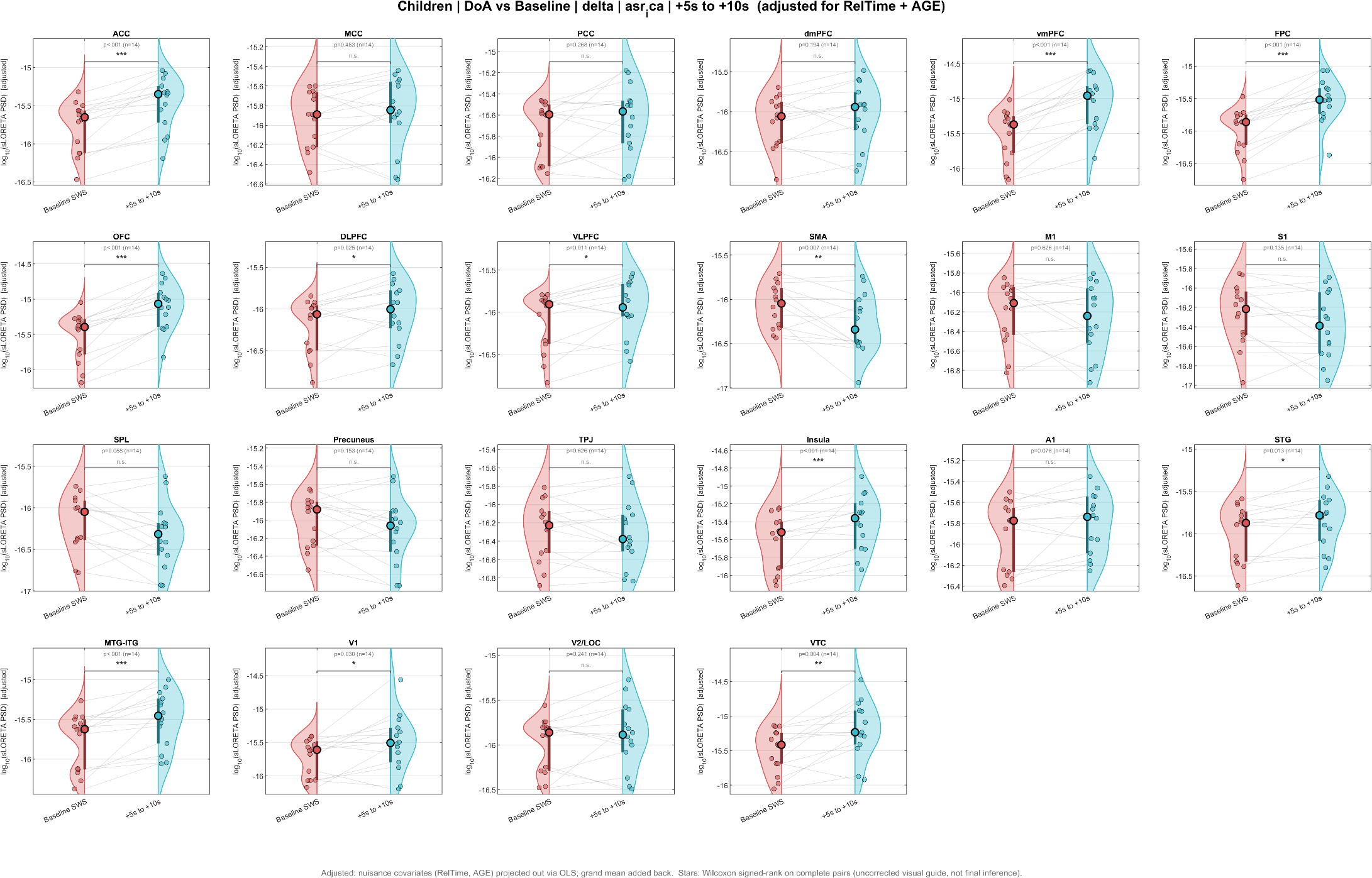


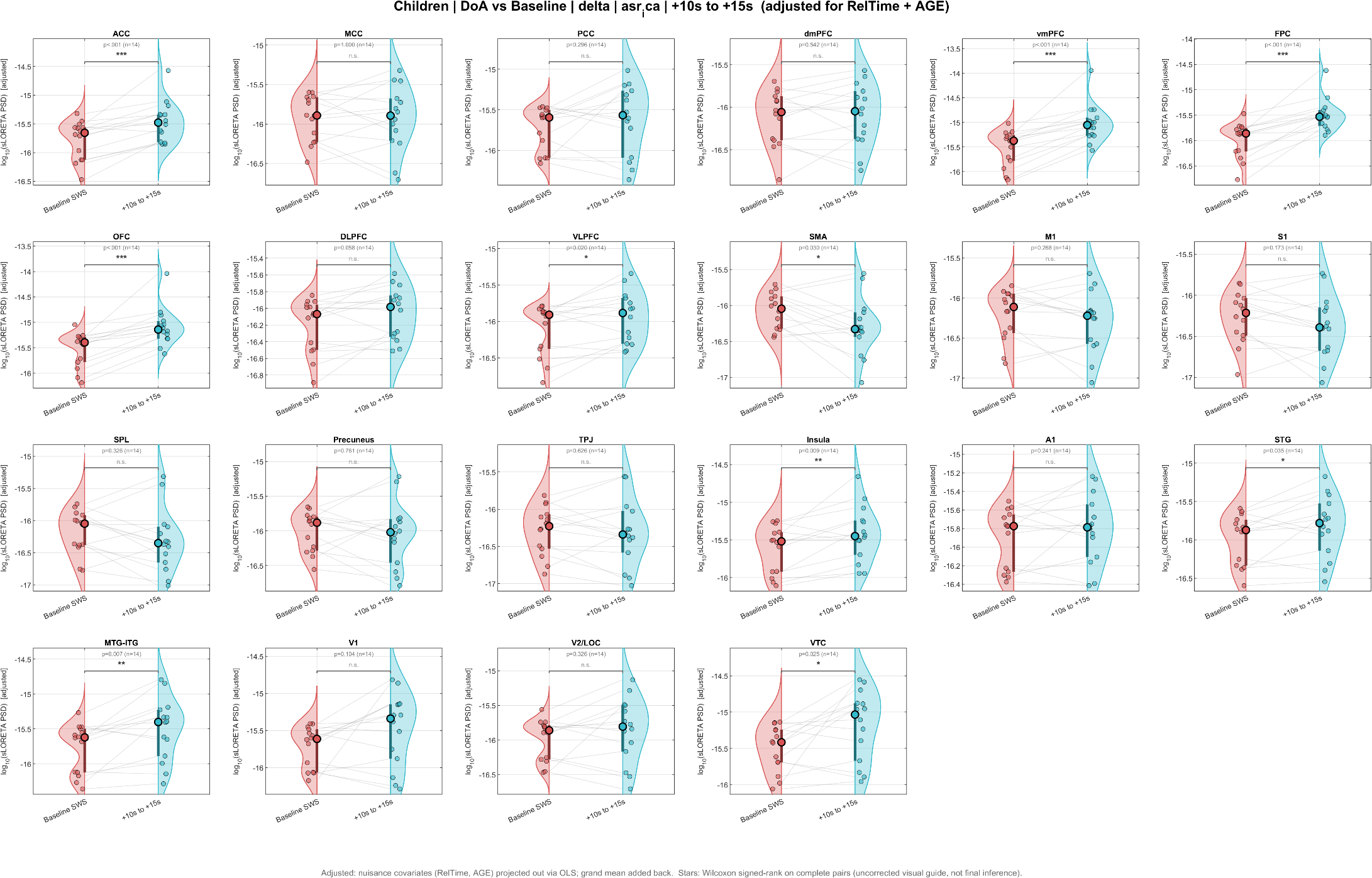


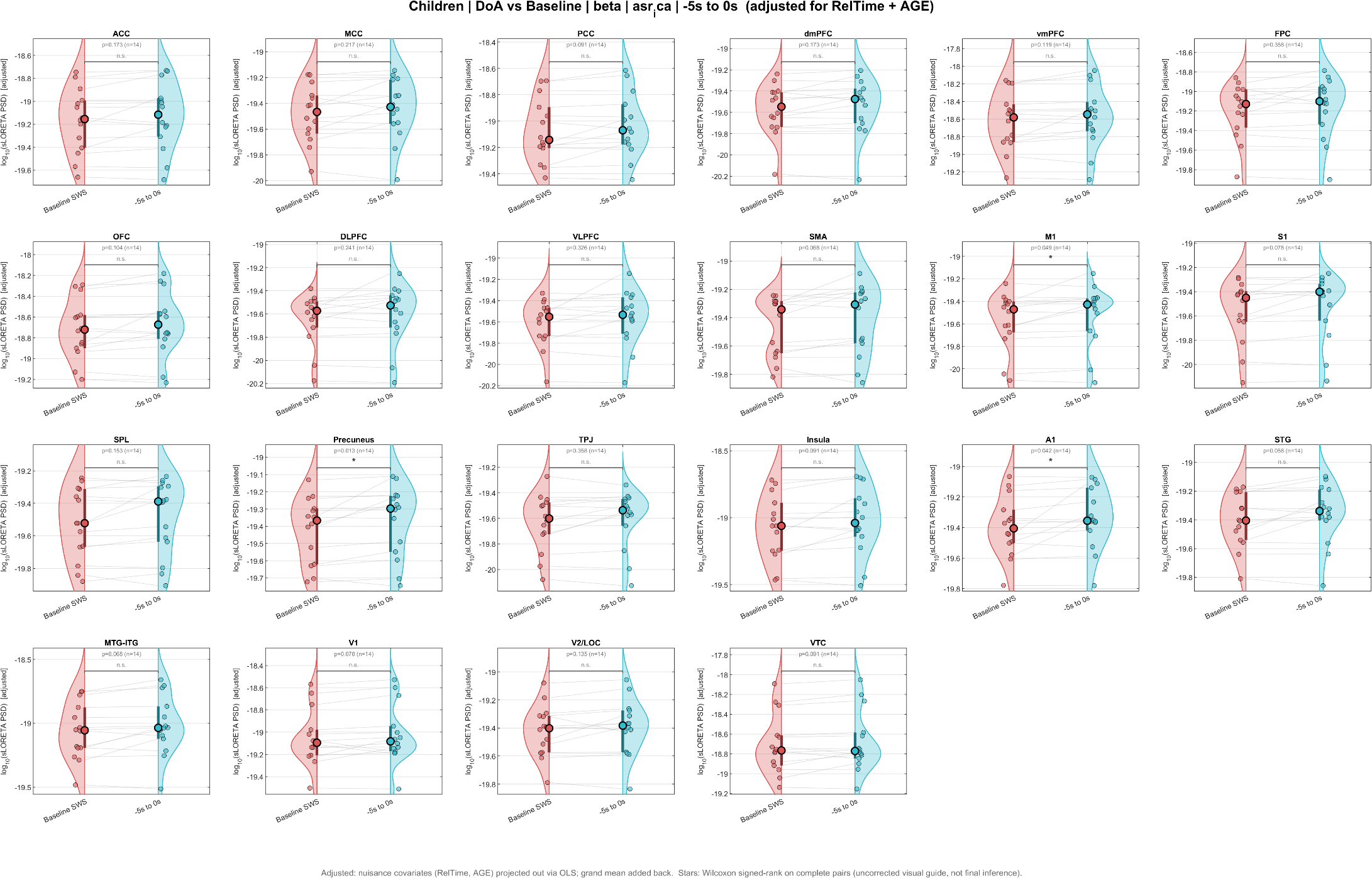


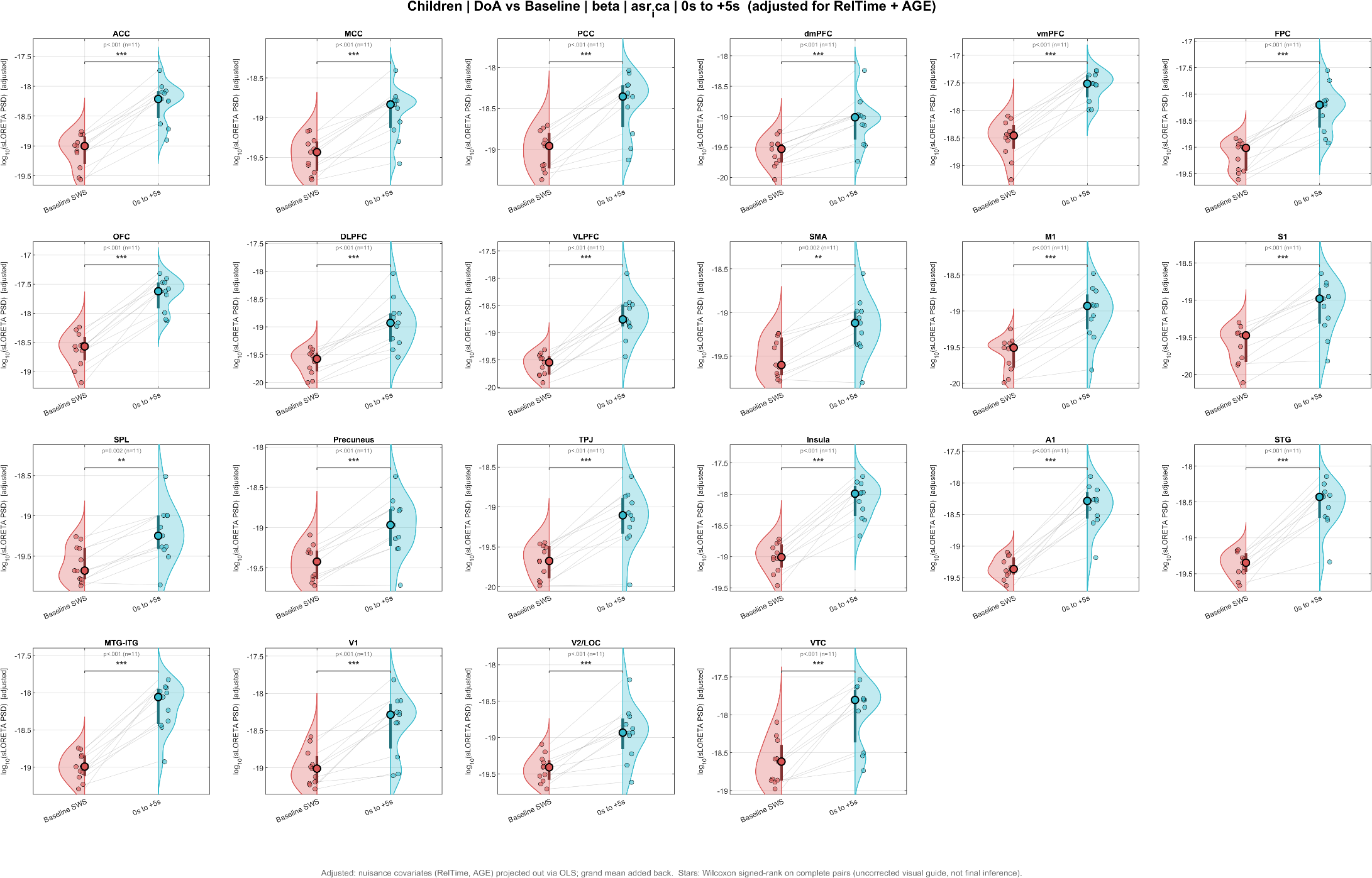


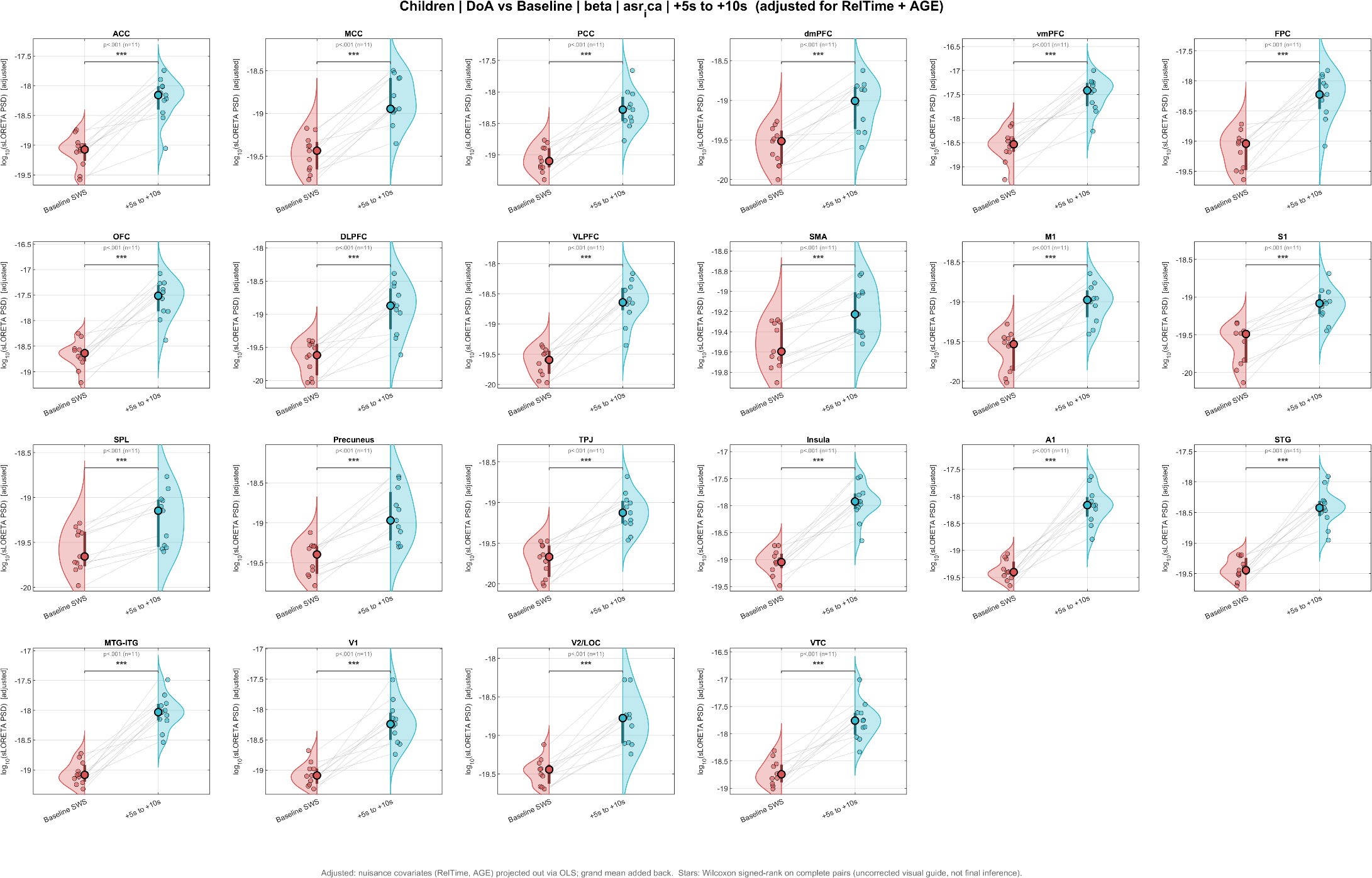


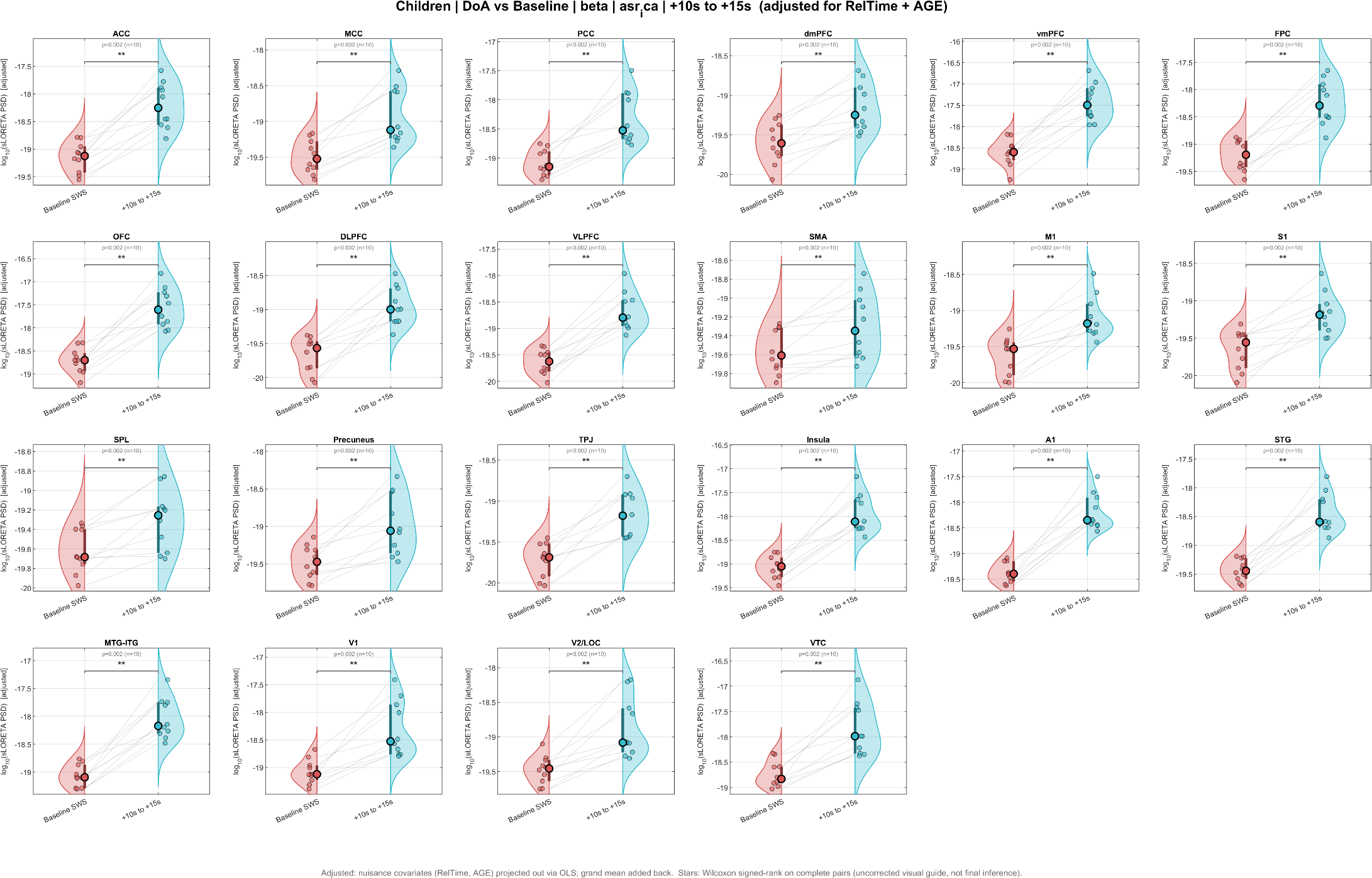


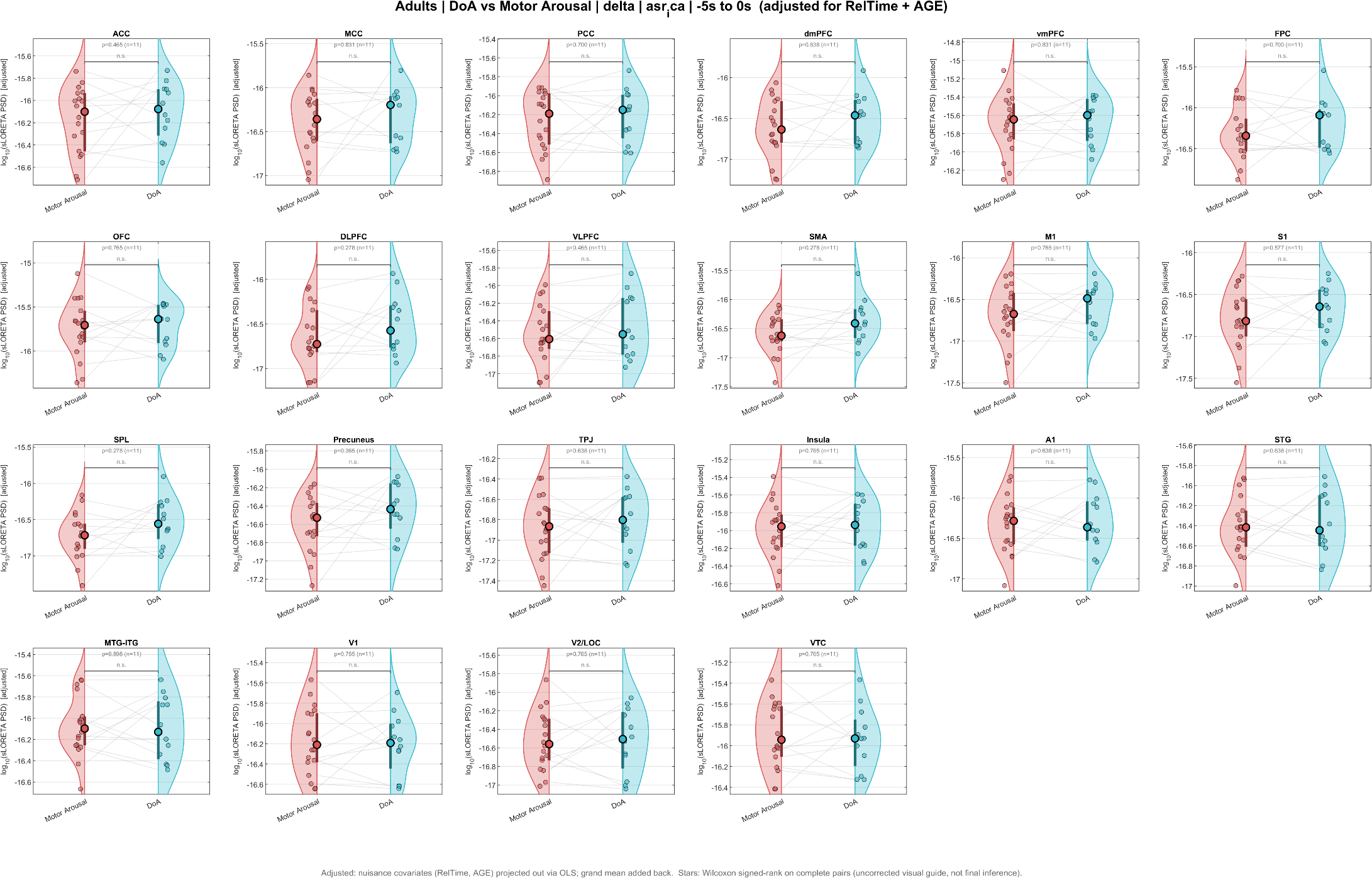


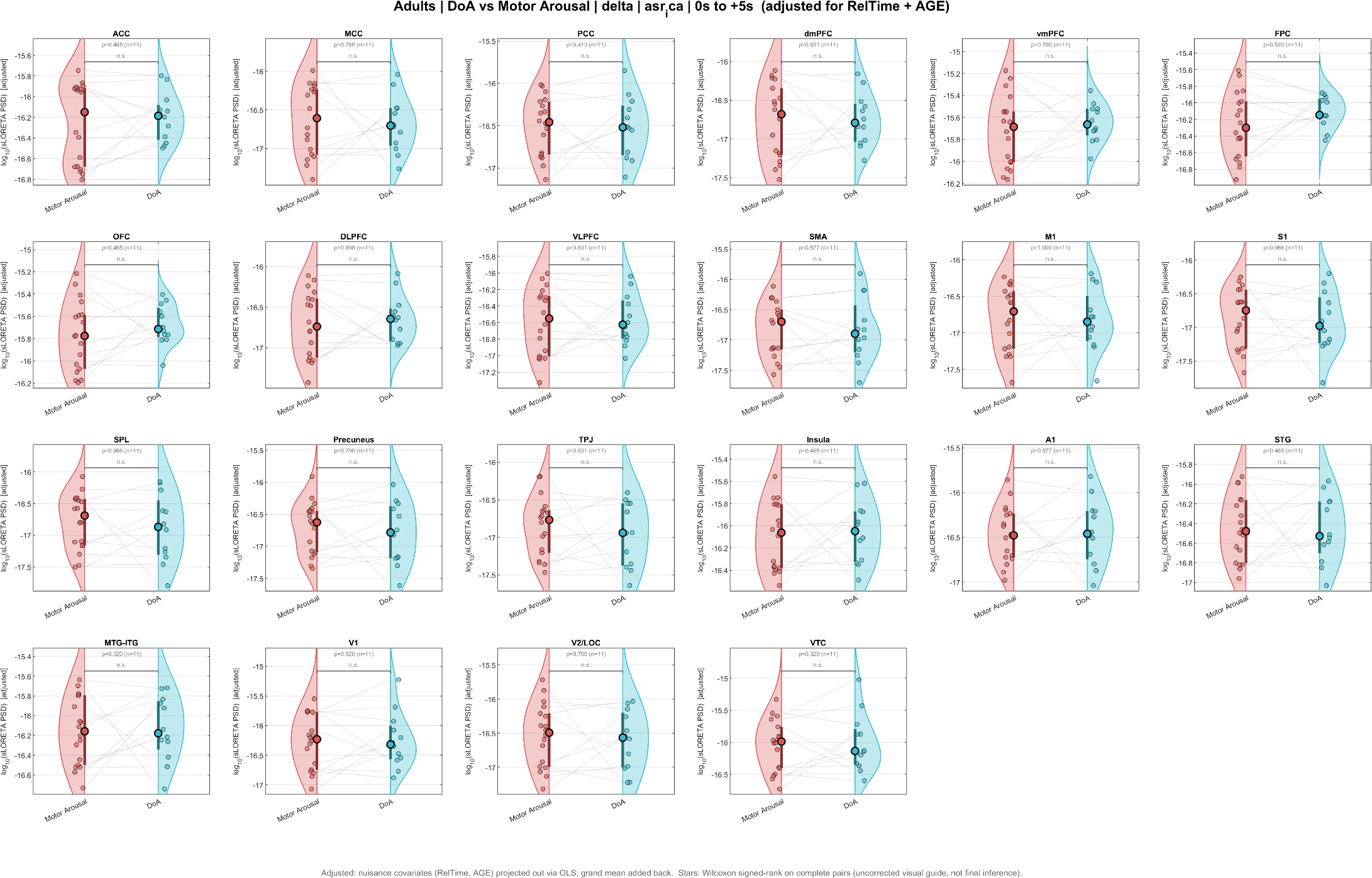


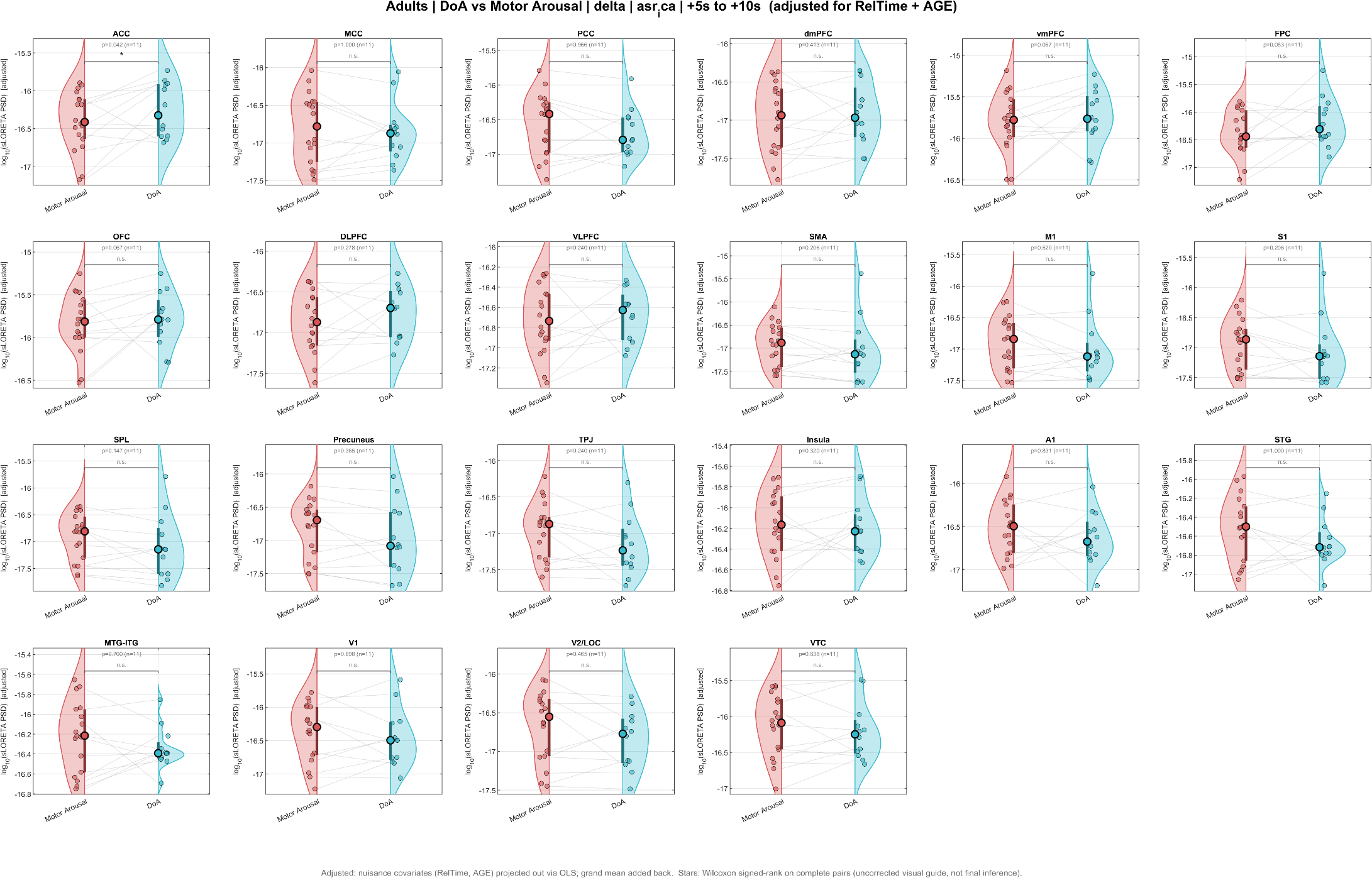


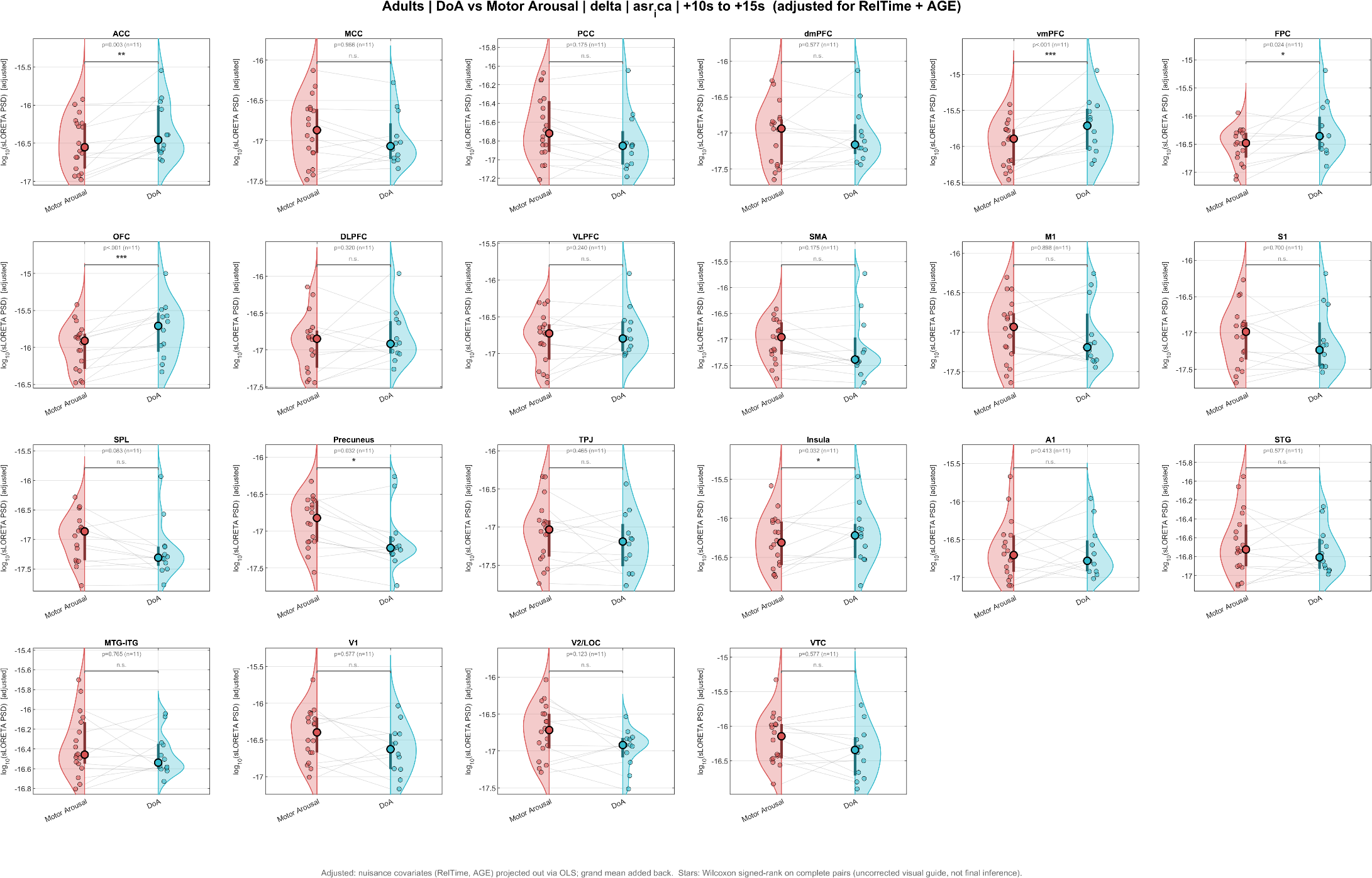


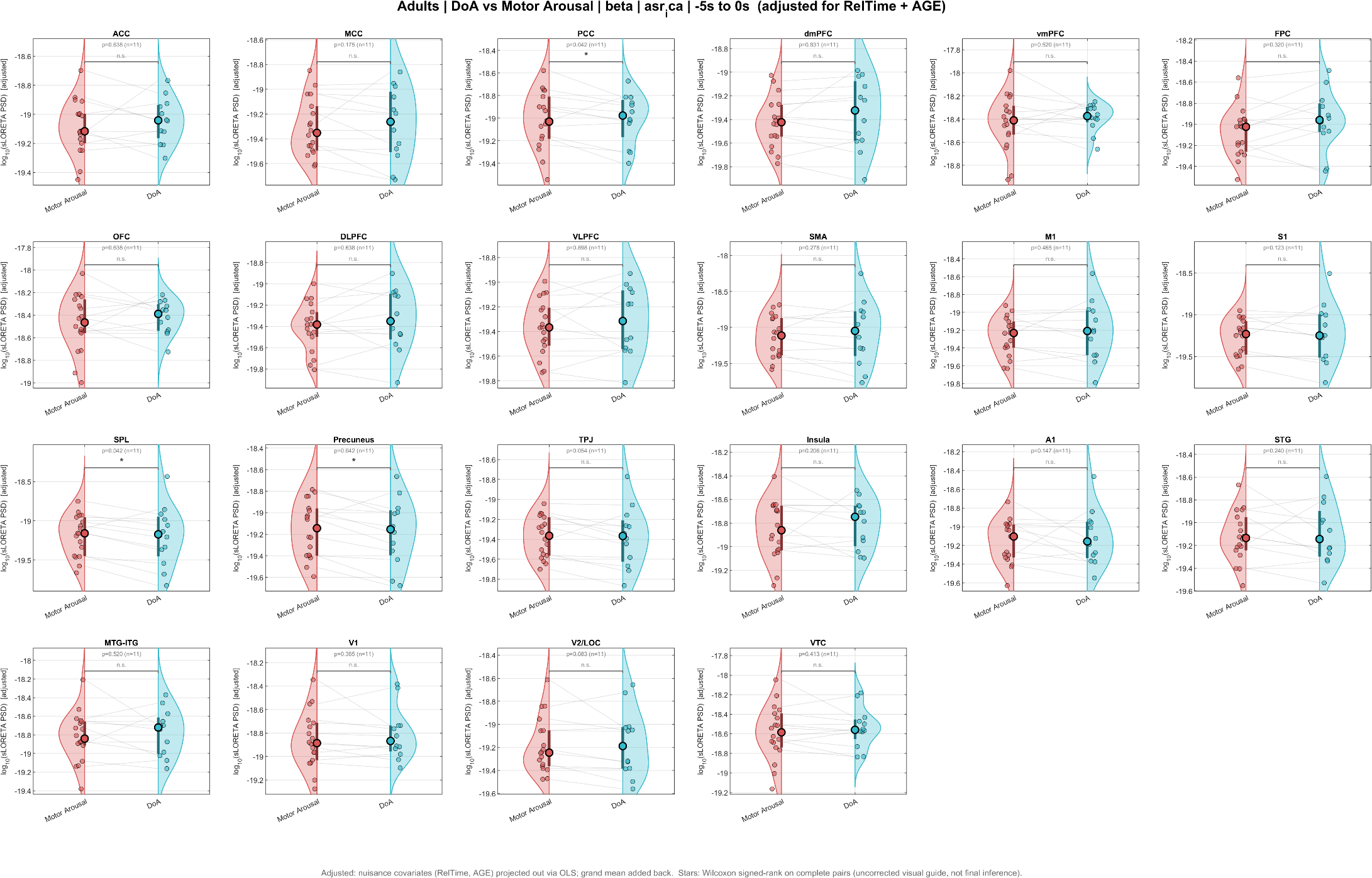


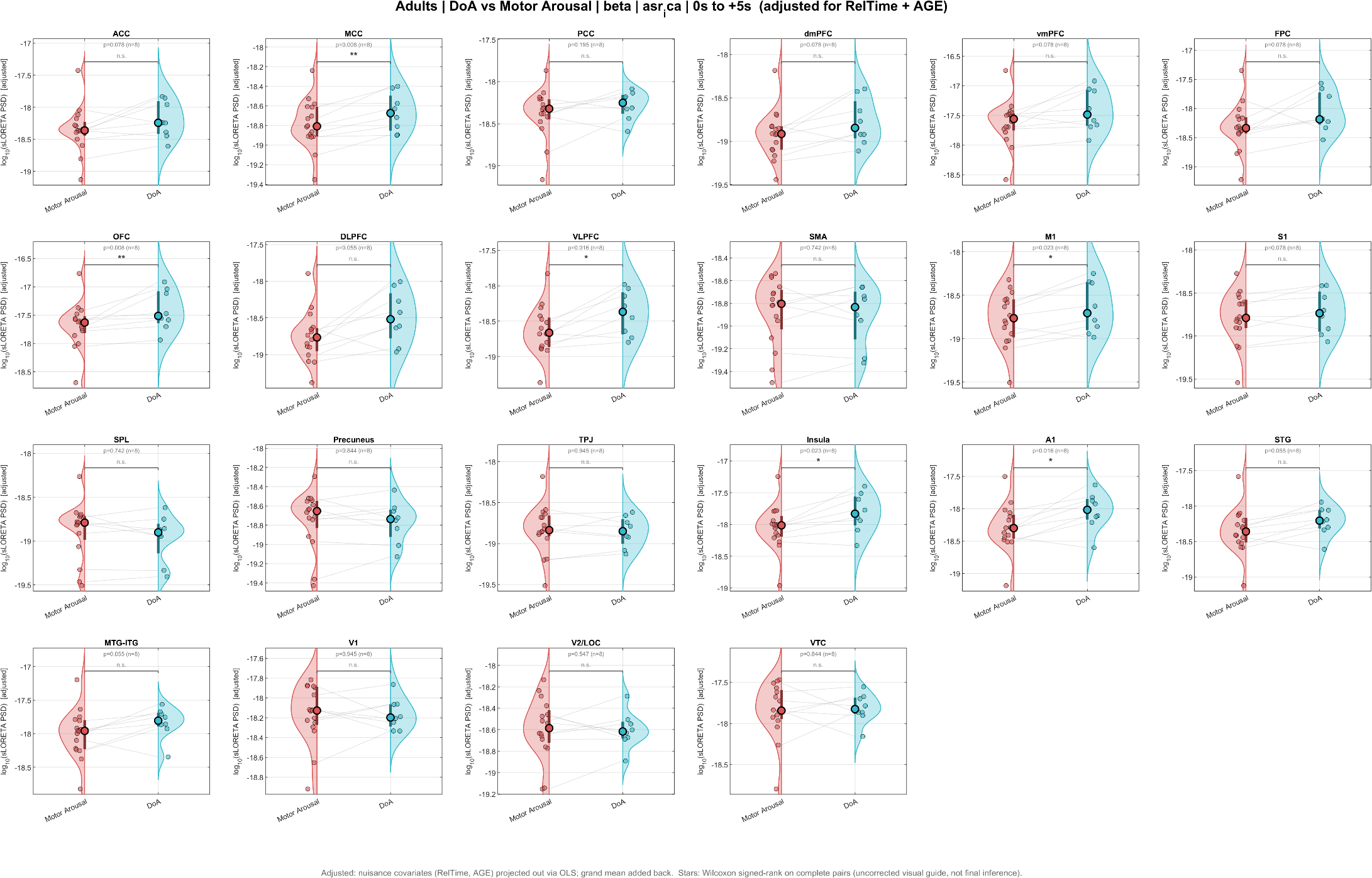


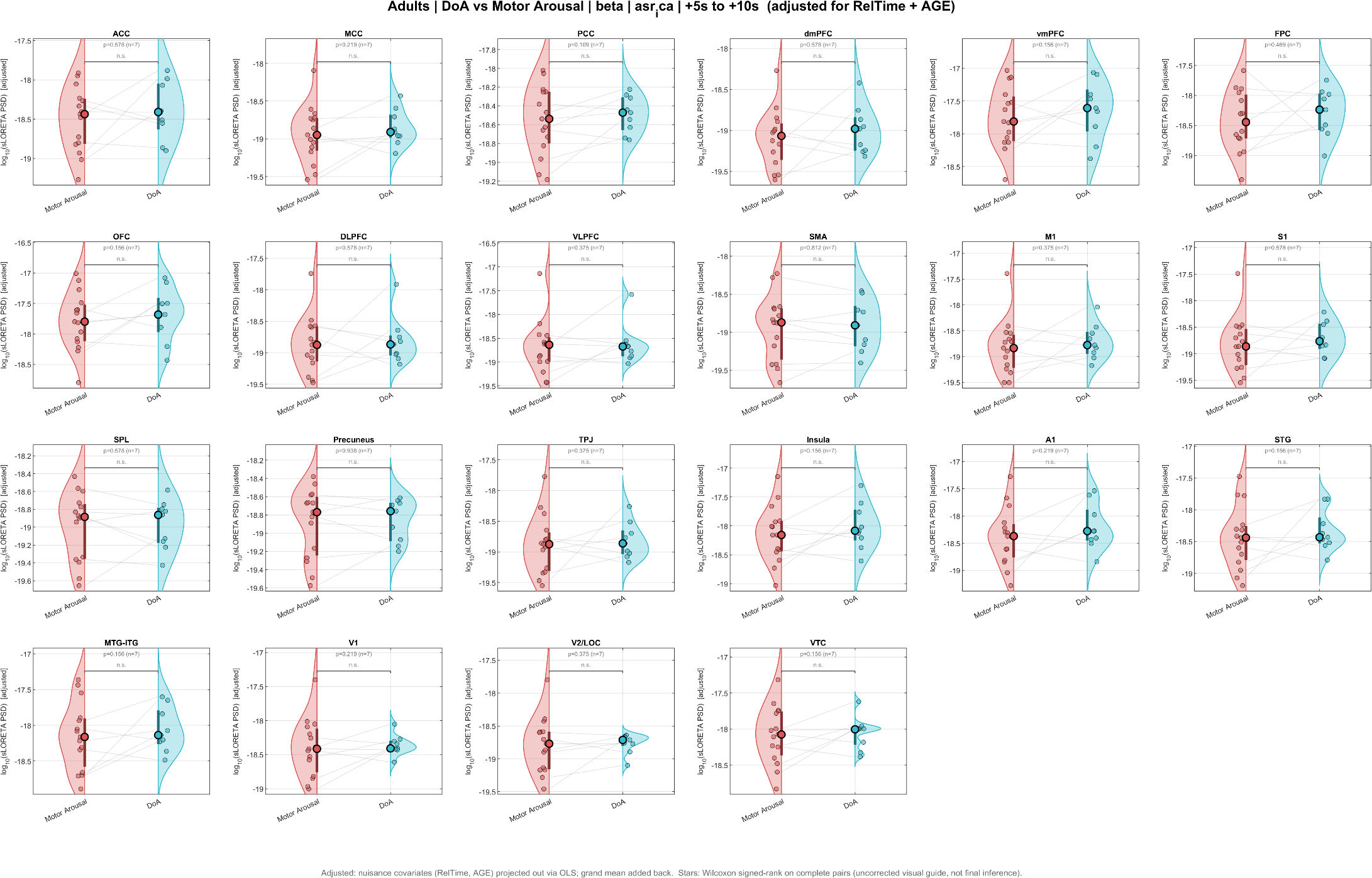


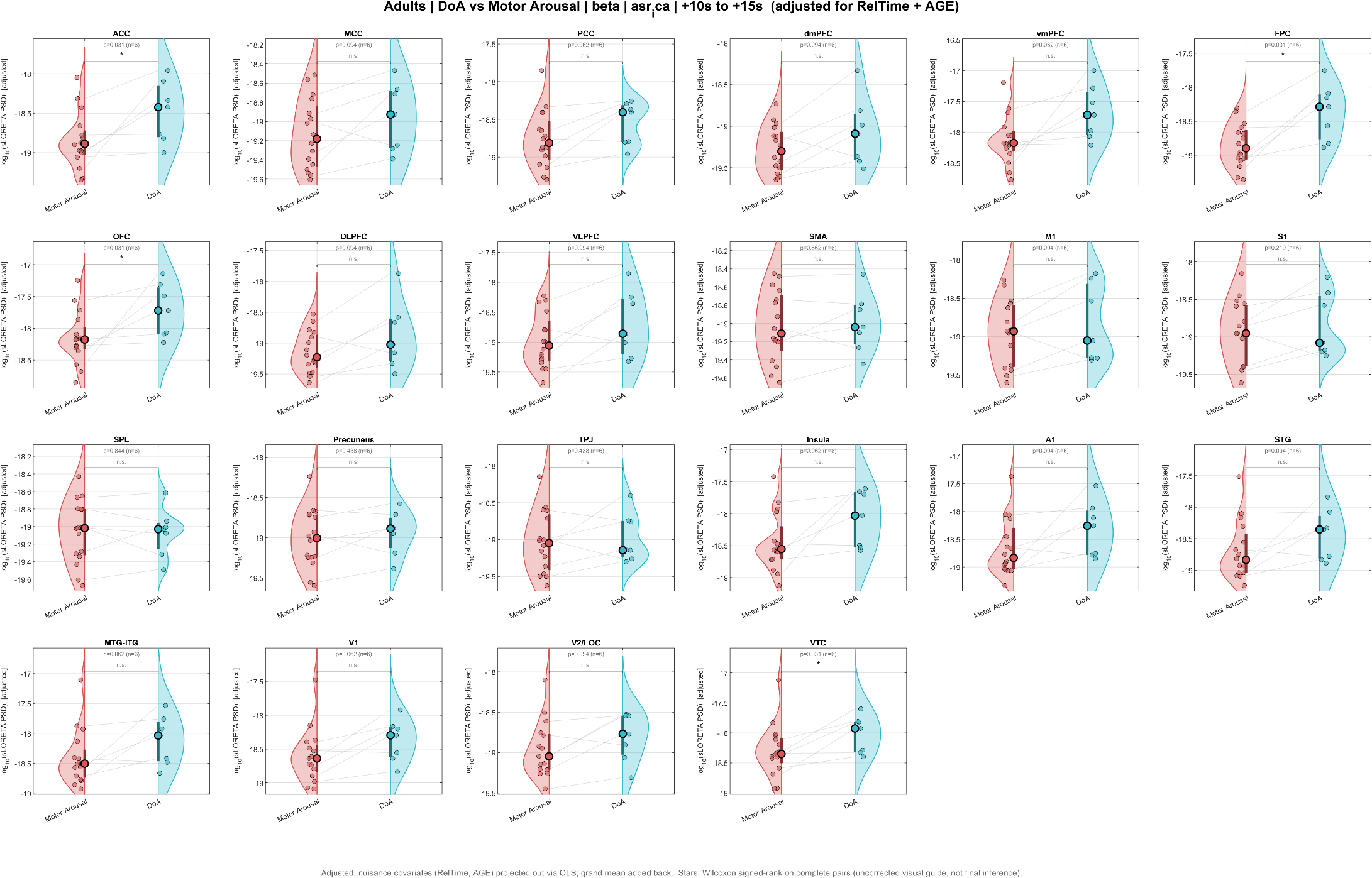


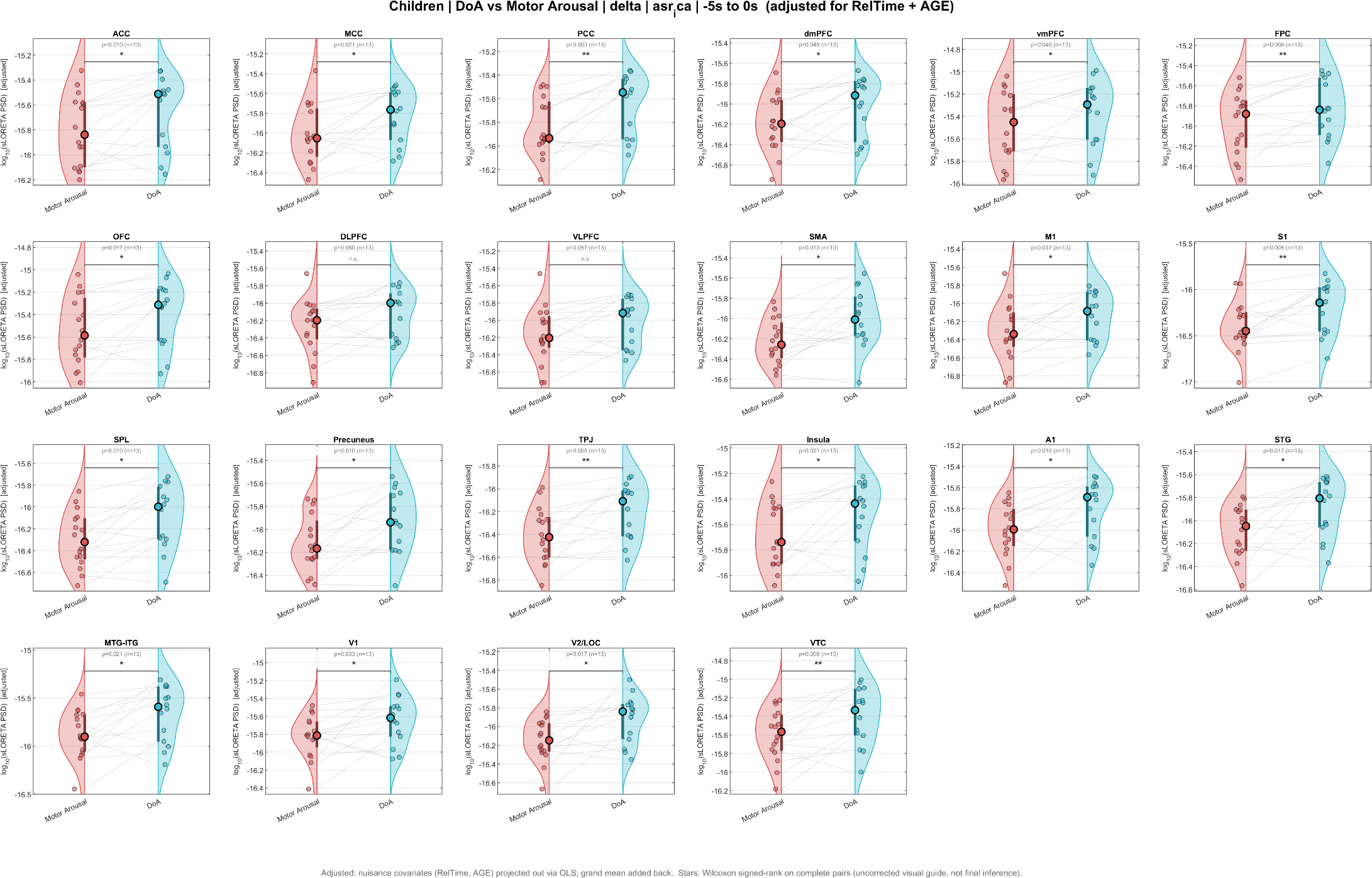


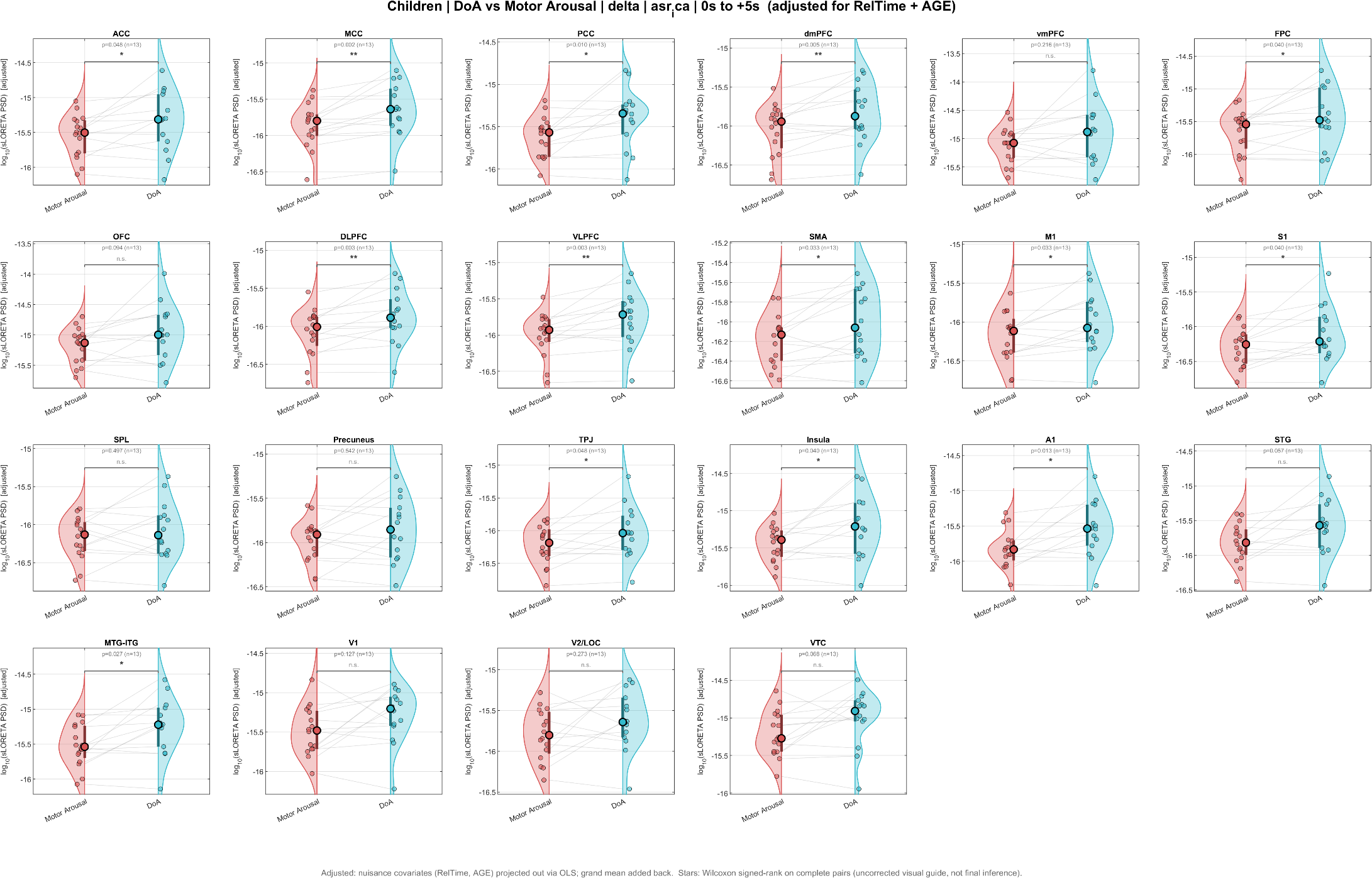


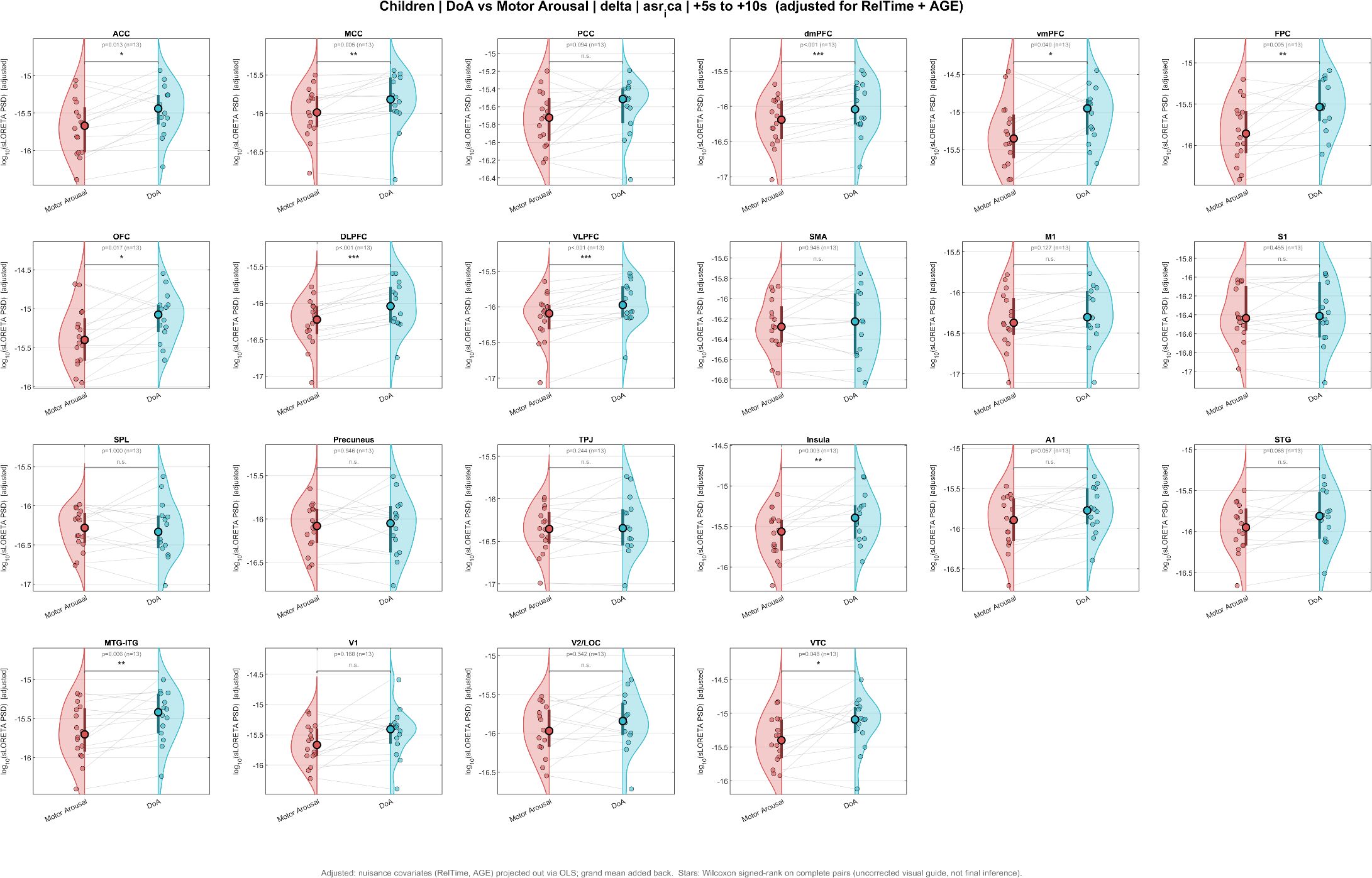


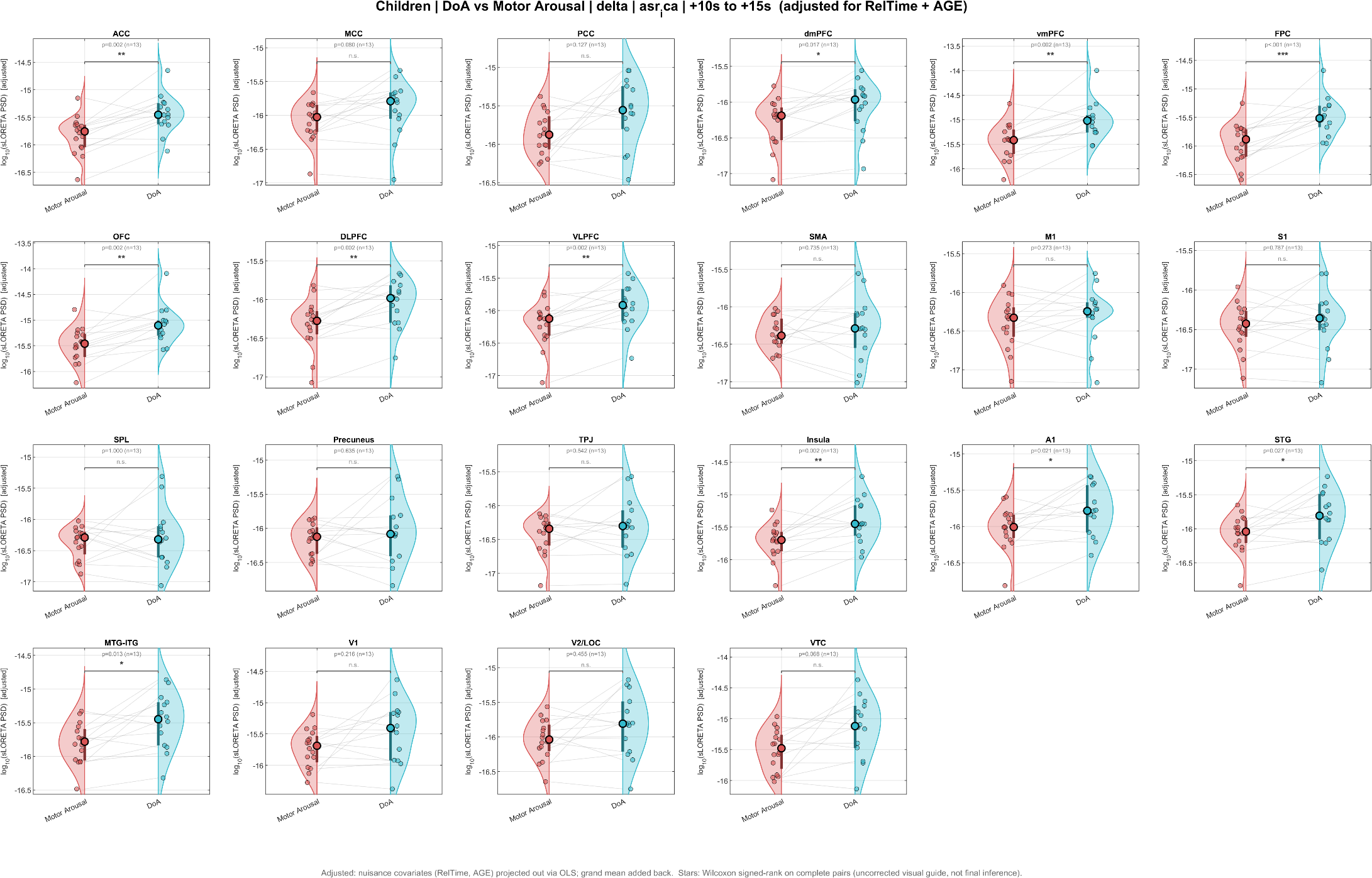


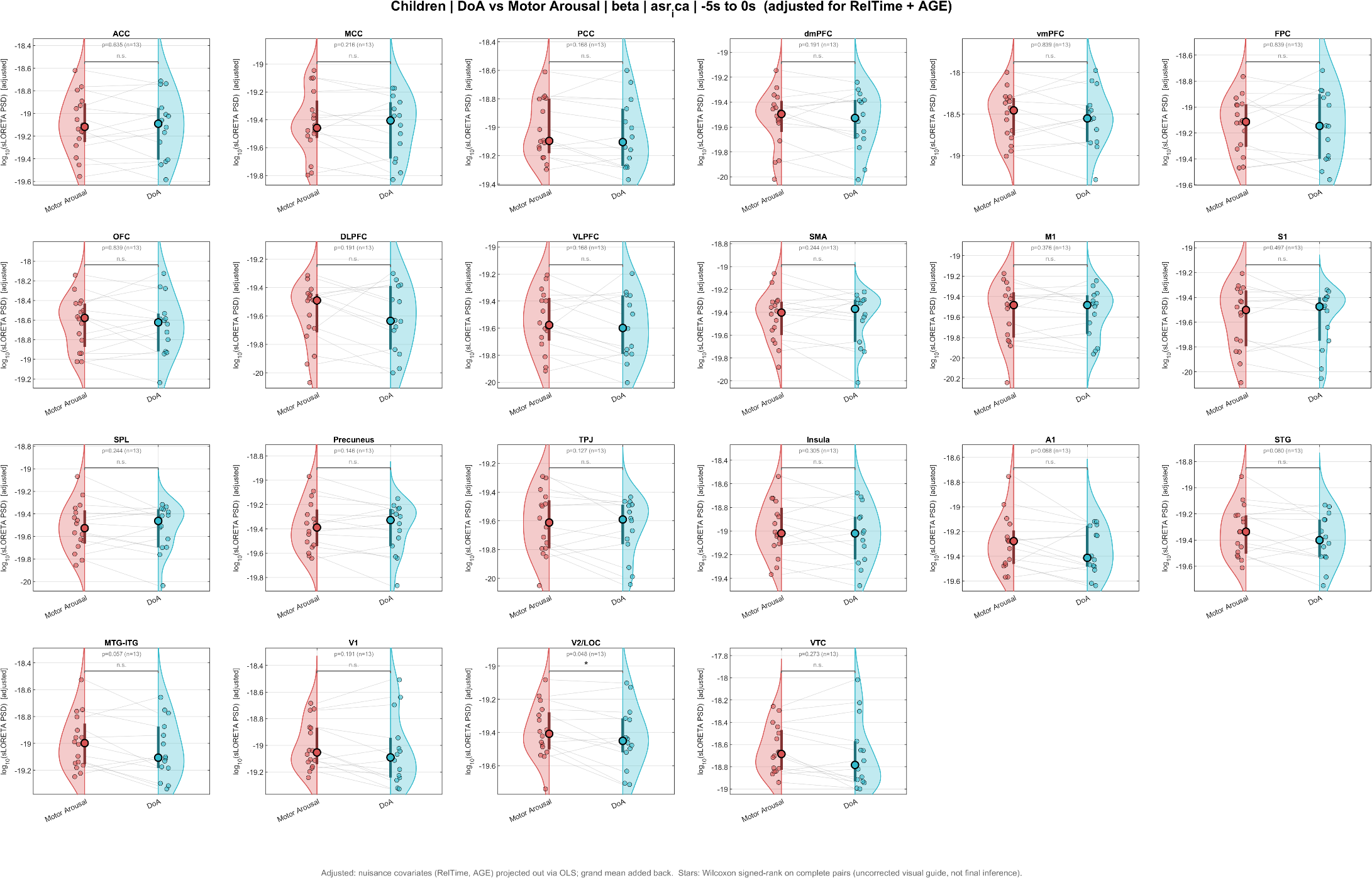


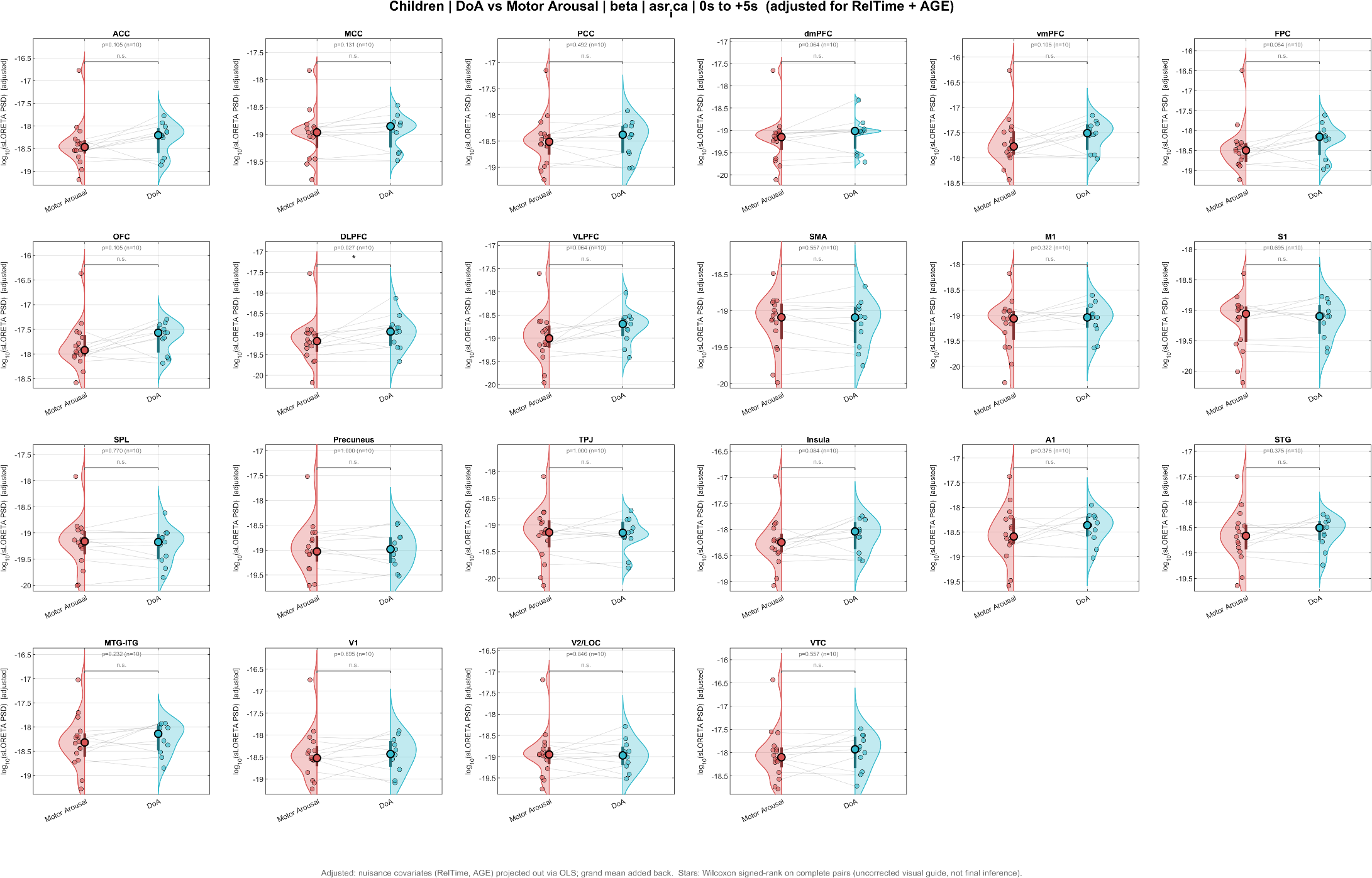


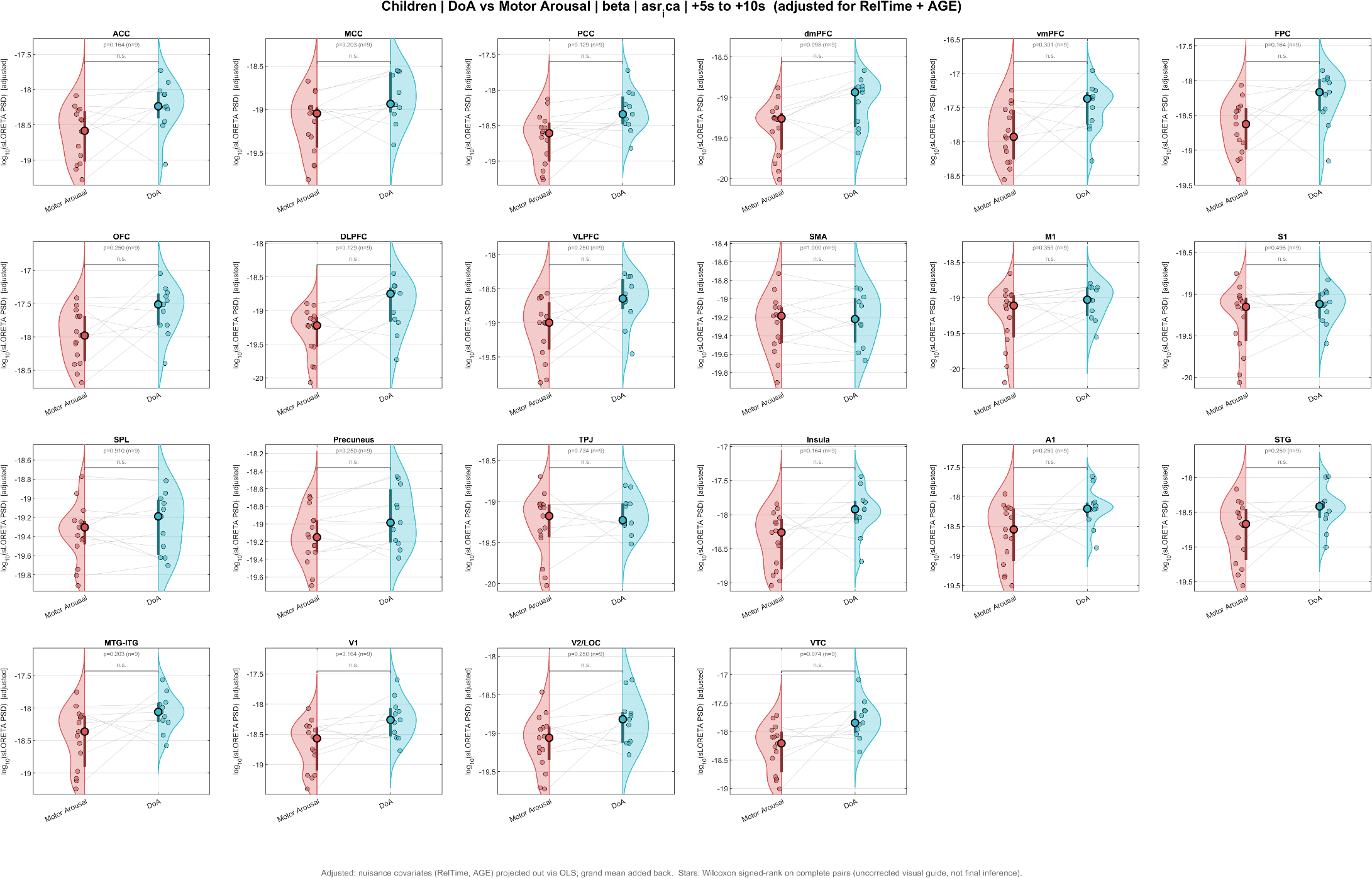


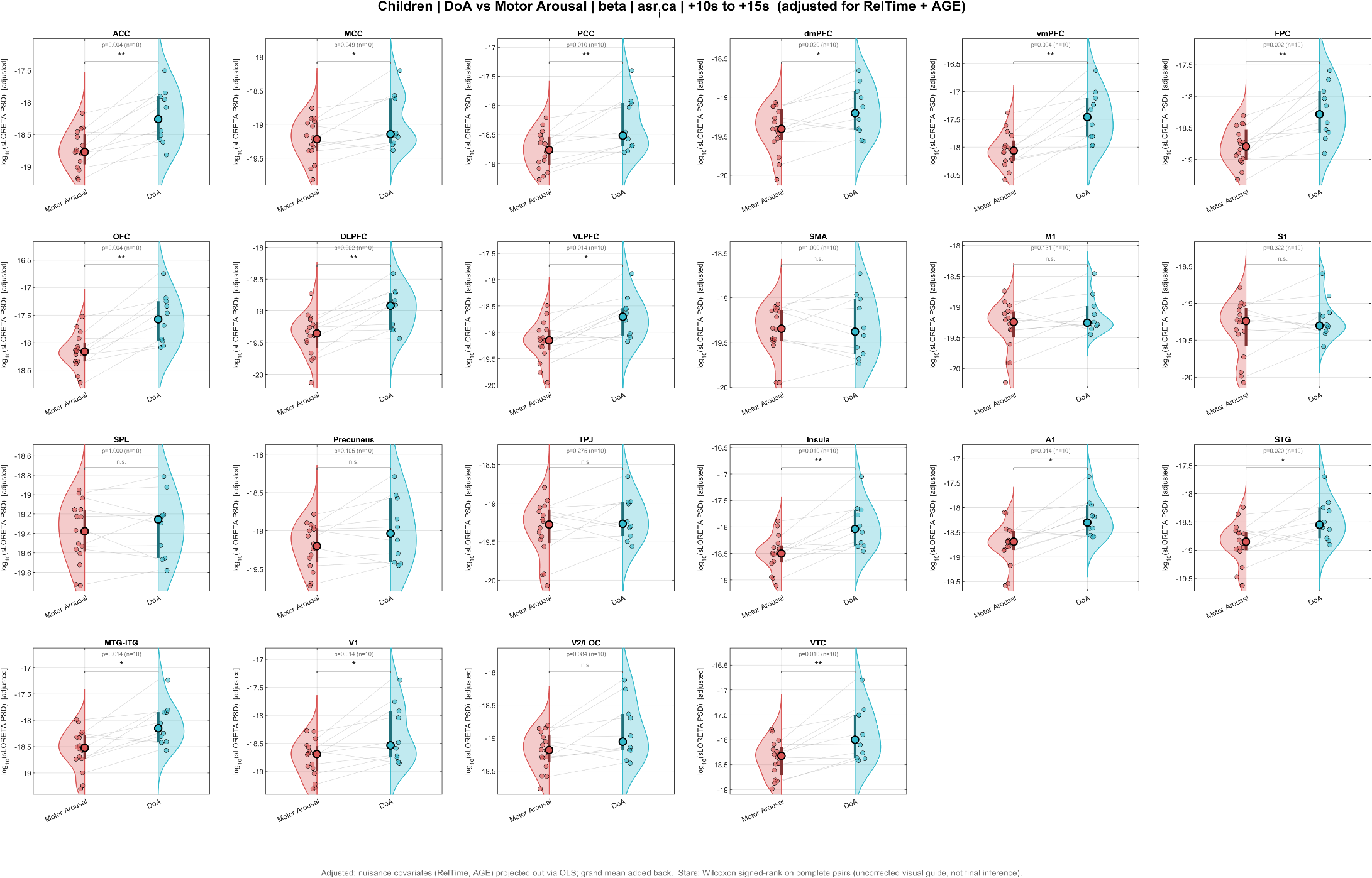

Supplement: supplementary_materials_Z2_zsag123 [file supplementary_materials_z2_zsag123.docx]
